# Supplementary material for: Discovery of Novel Diphenyl Acrylonitrile Derivatives That Promote Adult Rats’ Hippocampal Neurogenesis
Source: Int J Mol Sci. 2024 Jan 19;25(2):1241. doi: 10.3390/ijms25021241 (PMC10816640; doi:10.3390/ijms25021241)
Supplement: Supplementary file 1 [file ijms-25-01241-s001.zip › ijms-2830027-supplementary.pdf]

Supplementary materials for

Discovery of Novel diphenyl acrylonitrile derivatives that Promote Adult Rats' Hippocampal Neurogenesis

Si-Si Liu [1, #], Cong-Xuan Ma [1, #], Zheng-Yang Quan [2],

Jing Ding [1], Liang Yang [2], Si-Meng Liu [1], He-Ao Zhang [2], Hong Qing [*, 2], Jian-Hua Liang [*, 1]

1 Key Laboratory of Medical Molecule Science and Pharmaceutical Engineering, School of Chemistry and Chemical Engineering, Beijing Institute of Technology, Beijing 102488, China

2 School of Life Science, Beijing Institute of Technology, Beijing, 100081, China

[#] Contributed equally to this paper.

* Correspondence: hqing@bit.edu.cn(H.Q.); ljhbit@bit.edu.cn(J.-H.L.)

Contents

1. Table S1. NMR spectra checklist for the target compounds.
2. ^{1}H and ^{13}C NMR Spectra for the representative compounds.
3. HRMS for the representative compounds.
4. Pharmacokinetic experimental conditions of compound **32b**.
5. Figure S1. Results of dose-toxicity experiment for compounds **29b**, **32a**, and **32b**.

Table S1. NMR spectra checklist for the target compounds.

Compounds	^{1}H NMR	^{13}C NMR	HRMS
**8c**	√	√	√
**8d**	√	√	√
**8e**	√	√	√
**8g**	√	√	√
**8h**	√	√	√
**8j**	√	√	√
**8k**	√	√	√
**8l**	√	√	√
**9b**	√	√	√
**12f**	√	√	√
**18b**	√	√	√
**23b**	√	√	√
**23i**	√	√	√
**24b**	√	√	√
**29a**	√	√	√
**29b**	√	√	√
**32a**	√	√	√
**32b**	√	√	√
**36a**	√	√	√
**39b**	√	√	√
**42b**	√	√	√

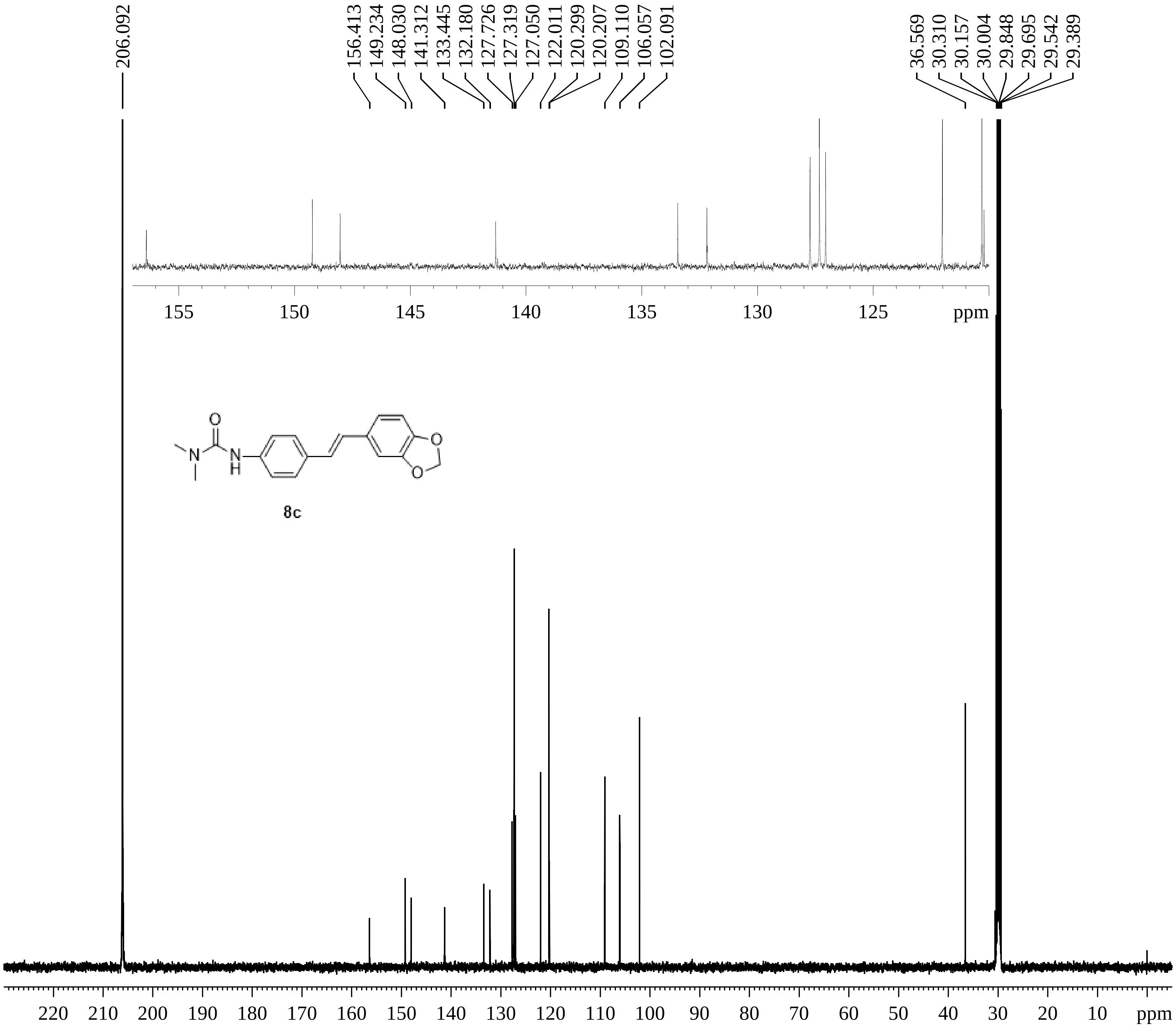

Current Data Parameters
NAME NMR15110917-WS-82
EXPNO 11
PROCNO 1

F2 - Acquisition Parameters
Date_ 20151127
Time 21.42
INSTRUM spect
PROBHD 5 mm PABBO BB-
PULPROG zgpg30
TD 65536
SOLVENT Acetone
NS 1024
DS 4
SWH 34722.223 Hz
FIDRES 0.529819 Hz
AQ 0.9437184 sec
RG 198.55
DW 14.400 usec
DE 6.50 usec
TE 303.1 K
D1 2.00000000 sec
D11 0.03000000 sec
TD0 1

======== CHANNEL f1 =======
SFO1 125.7703637 MHz
NUC1 13C
P1 8.99 usec
PLW1 125.88999939 W

======== CHANNEL f2 =======
SFO2 500.1320005 MHz
NUC2 1H
CPDPRG[2 waltz16
PCPD2 80.00 usec
PLW2 19.95299911 W
PLW12 0.39528000 W
PLW13 0.25297999 W

F2 - Processing parameters
SI 32768
SF 125.7576707 MHz
WDW EM
SSB 0
LB 1.00 Hz
GB 0
PC 1.40

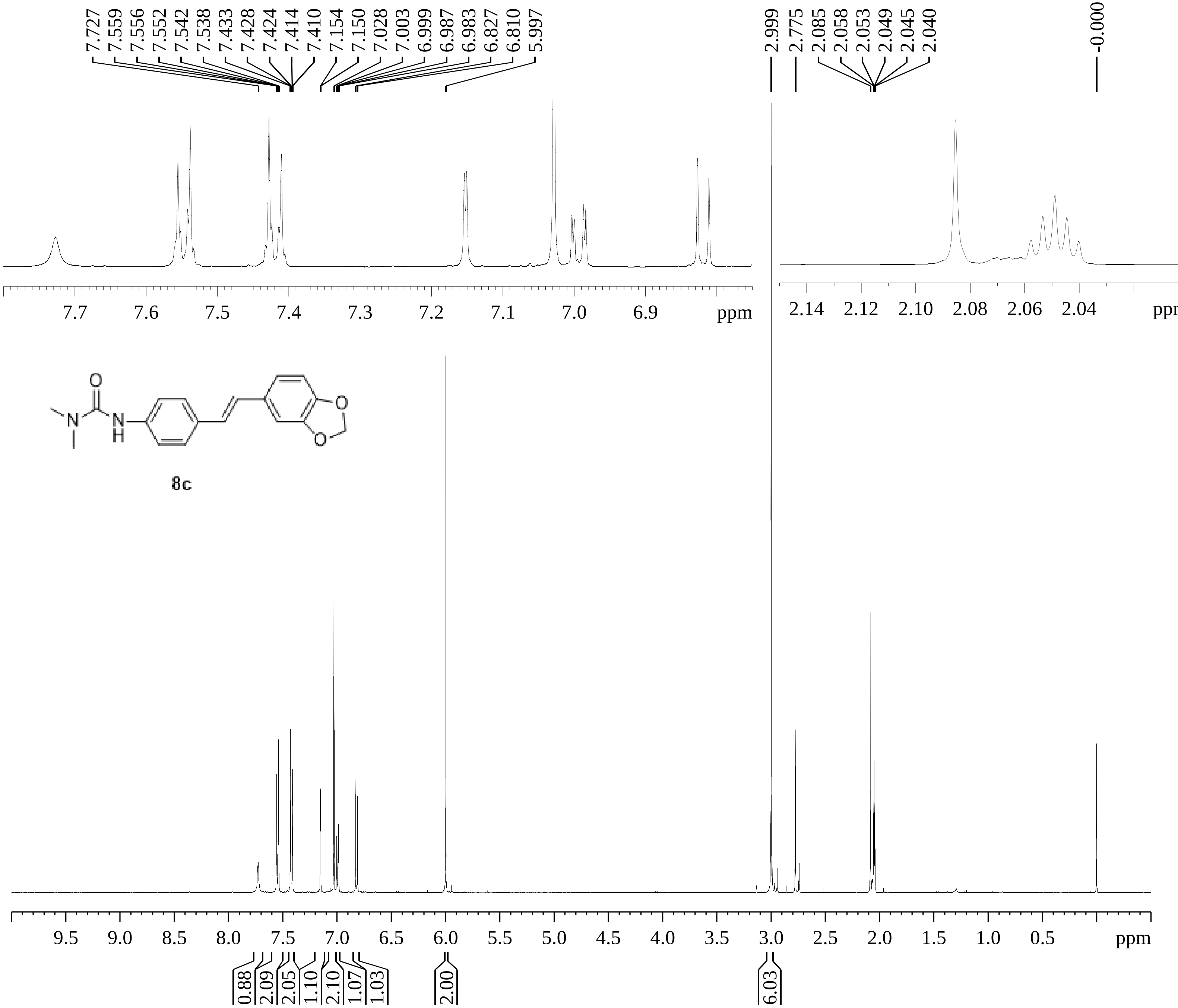

Current Data Parameters
NAME NMR15110917-WS-82
EXPNO 10
PROCNO 1

F2 - Acquisition Parameters
Date_ 20151126
Time 13.00
INSTRUM spect
PROBHD 5 mm PABBO BB-
PULPROG zg30
TD 65536
SOLVENT Acetone
NS 128
DS 2
SWH 10000.000 Hz
FIDRES 0.152588 Hz
AQ 3.2767999 sec
RG 160.92
DW 50.000 usec
DE 6.50 usec
TE 303.2 K
D1 1.00000000 sec
TD0 1

======== CHANNEL f1 =======
SFO1 500.1330885 MHz
NUC1 1H
P1 11.26 usec
PLW1 19.95299911 W

F2 - Processing parameters
SI 65536
SF 500.1300088 MHz
WDW EM
SSB 0
LB 0.30 Hz
GB 0
PC 1.00

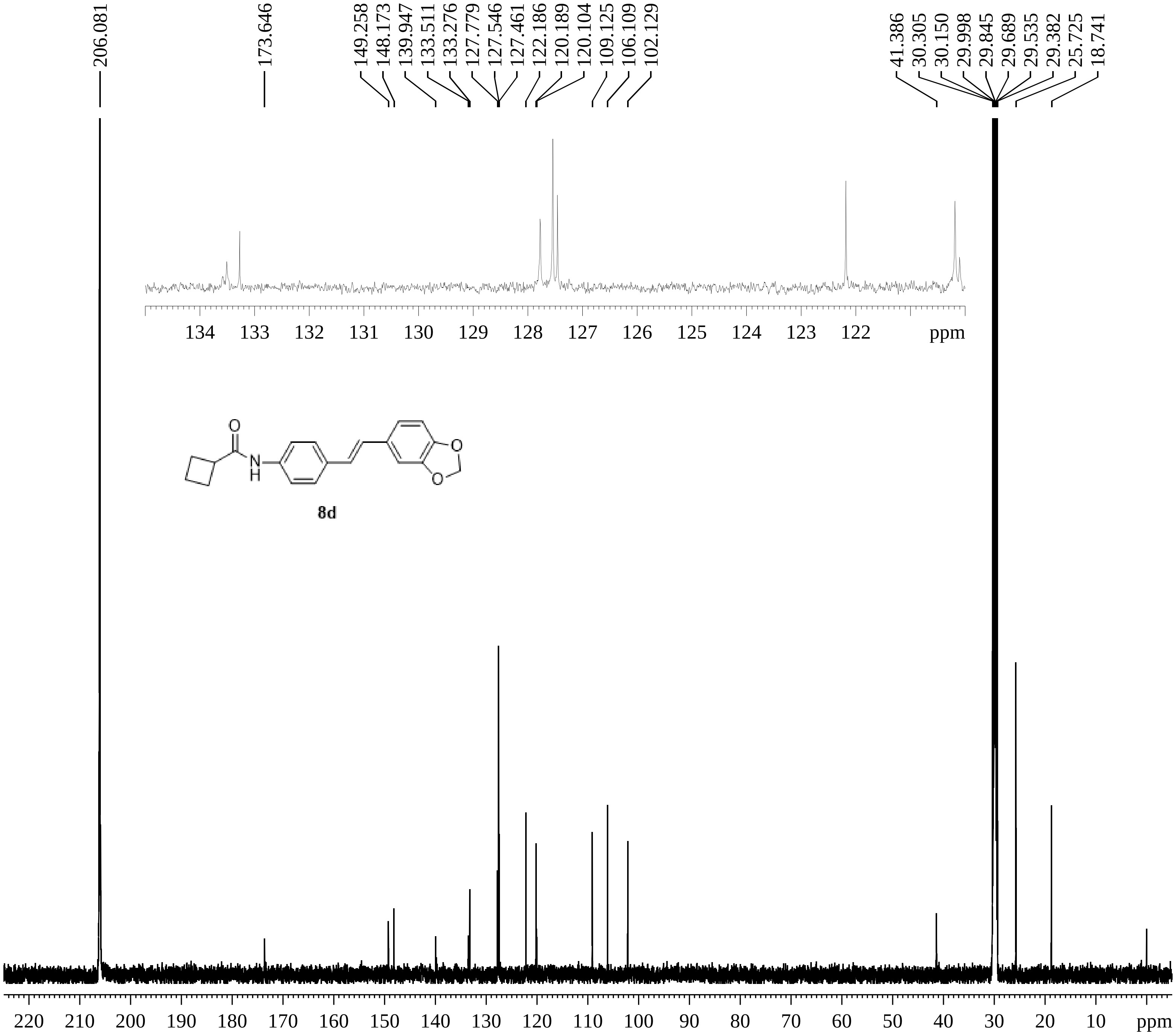

8d

Current Data Parameters
NAME NMR15110918-WS-83
EXPNO 11
PROCNO 1

F2 - Acquisition Parameters
Date_ 20151127
Time 22.45
INSTRUM spect
PROBHD 5 mm PABBO BB-
PULPROG zgpg30
TD 65536
SOLVENT Acetone
NS 1200
DS 4
SWH 34722.223 Hz
FIDRES 0.529819 Hz
AQ 0.9437184 sec
RG 198.55
DW 14.400 usec
DE 6.50 usec
TE 303.2 K
D1 2.00000000 sec
D11 0.03000000 sec
TD0 1

======== CHANNEL f1 =======
SFO1 125.7703637 MHz
NUC1 13C
P1 8.99 usec
PLW1 125.88999939 W

======== CHANNEL f2 =======
SFO2 500.1320005 MHz
NUC2 1H
CPDPRG[2 waltz16
PCPD2 80.00 usec
PLW2 19.95299911 W
PLW12 0.39528000 W
PLW13 0.25297999 W

F2 - Processing parameters
SI 32768
SF 125.7576707 MHz
WDW EM
SSB 0
LB 1.00 Hz
GB 0
PC 1.40

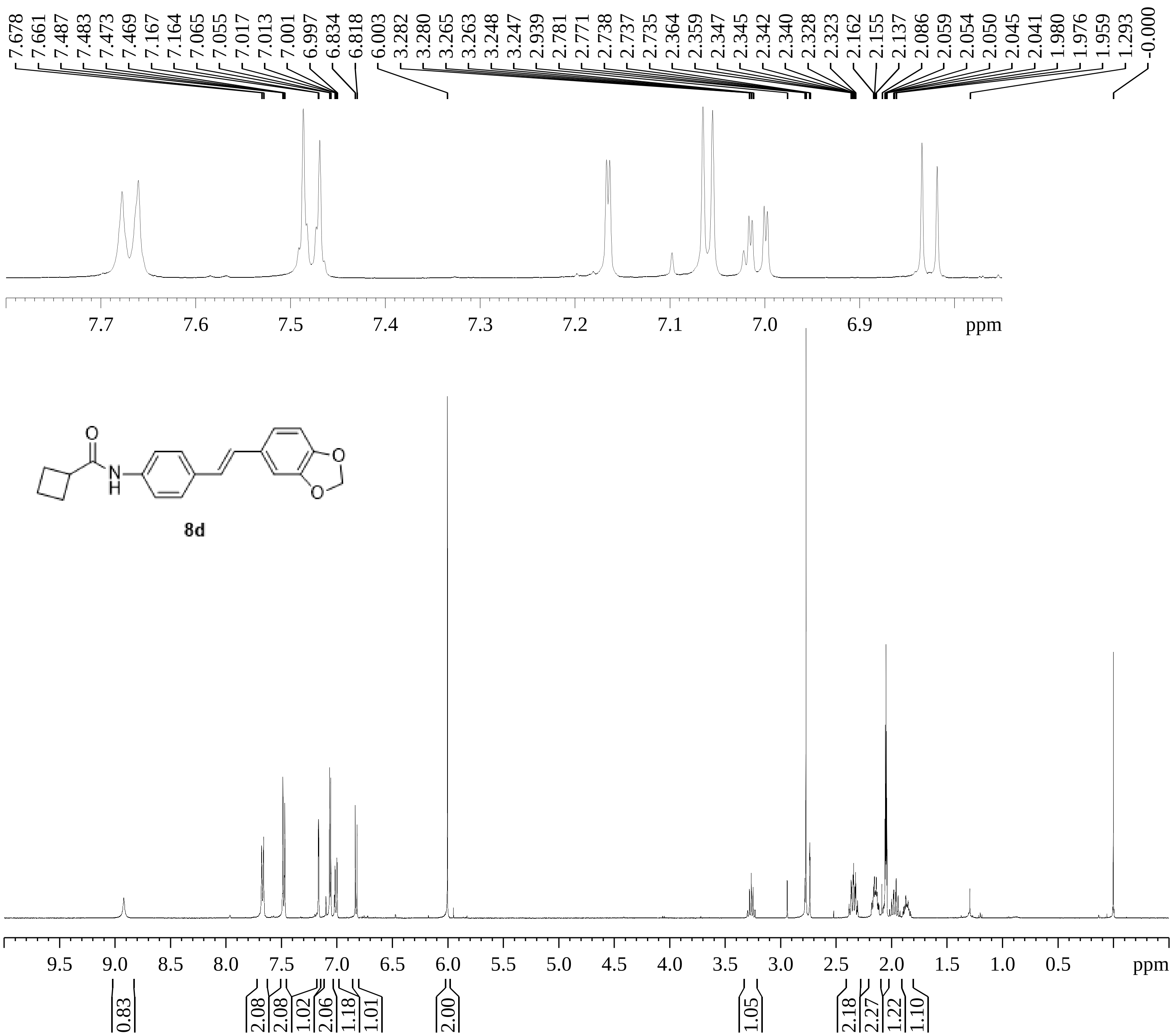

8d

Current Data Parameters
NAME NMR15110918-WS-83
EXPNO 10
PROCNO 1

F2 - Acquisition Parameters
Date_ 20151126
Time 13.12
INSTRUM spect
PROBHD 5 mm PABBO BB-
PULPROG zg30
TD 65536
SOLVENT Acetone
NS 128
DS 2
SWH 10000.000 Hz
FIDRES 0.152588 Hz
AQ 3.2767999 sec
RG 198.55
DW 50.000 usec
DE 6.50 usec
TE 303.2 K
D1 1.00000000 sec
TD0 1

======== CHANNEL f1 =======
SFO1 500.1330885 MHz
NUC1 1H
P1 11.26 usec
PLW1 19.95299911 W

F2 - Processing parameters
SI 65536
SF 500.1300085 MHz
WDW EM
SSB 0
LB 0.30 Hz
GB 0
PC 1.00

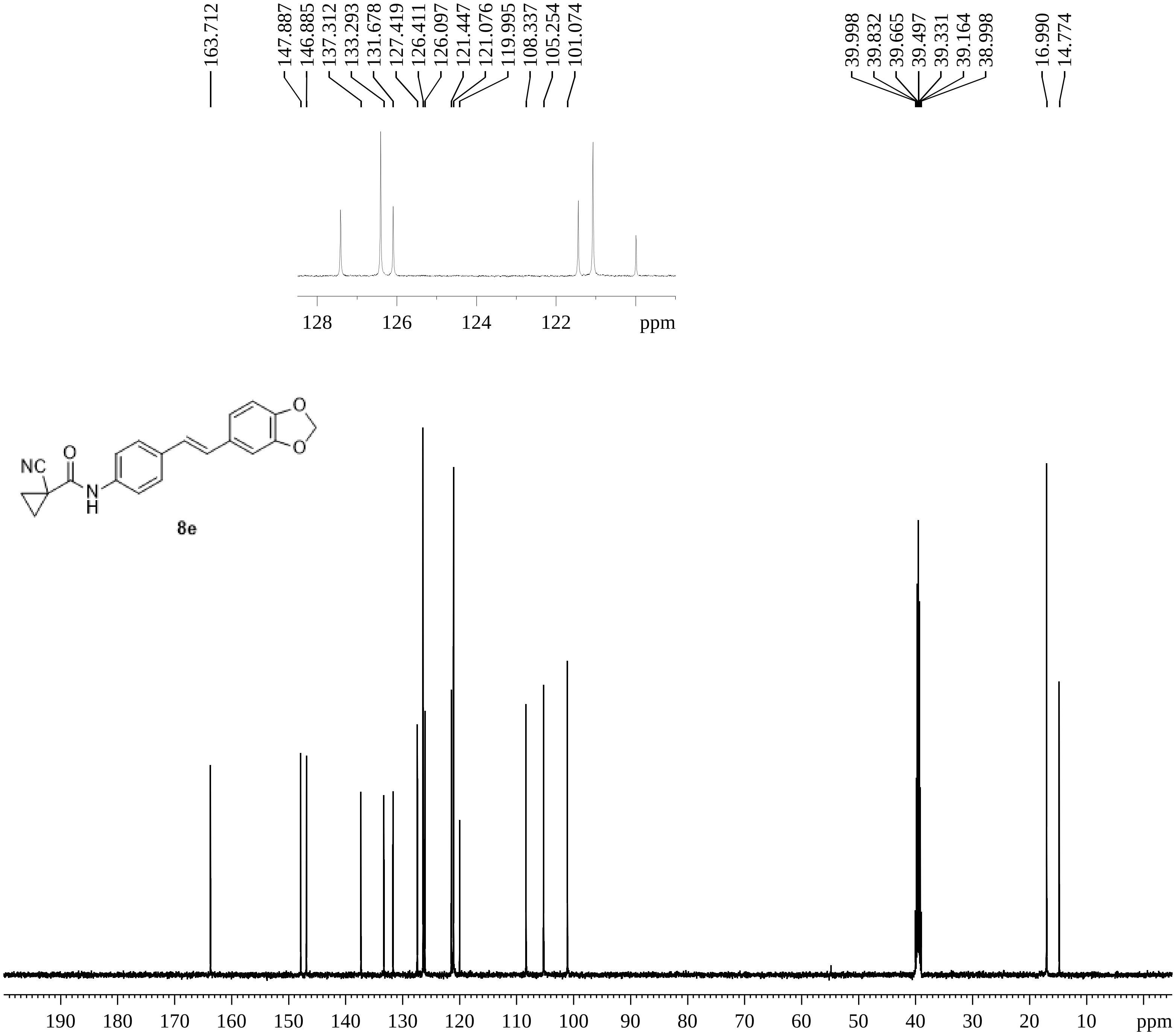

Current Data Parameters
NAME NMR16100058-LSS-50
EXPNO 2
PROCNO 1

F2 - Acquisition Parameters
Date_ 20161017
Time 17.04
INSTRUM spect
PROBHD 5 mm PABBO BB-
PULPROG zgpg30
TD 65536
SOLVENT DMSO
NS 64
DS 4
SWH 34722.223 Hz
FIDRES 0.529819 Hz
AQ 0.9437184 sec
RG 198.55
DW 14.400 usec
DE 6.50 usec
TE 298.3 K
D1 2.00000000 sec
D11 0.03000000 sec
TD0 1

======== CHANNEL f1 =======
SFO1 125.7703637 MHz
NUC1 13C
P1 9.12 usec
PLW1 125.88999939 W

======== CHANNEL f2 =======
SFO2 500.1320005 MHz
NUC2 1H
CPDPRG[2 waltz16
PCPD2 80.00 usec
PLW2 19.95299911 W
PLW12 0.41303000 W
PLW13 0.26434001 W

F2 - Processing parameters
SI 32768
SF 125.7578475 MHz
WDW EM
SSB 0
LB 1.00 Hz
GB 0
PC 1.40

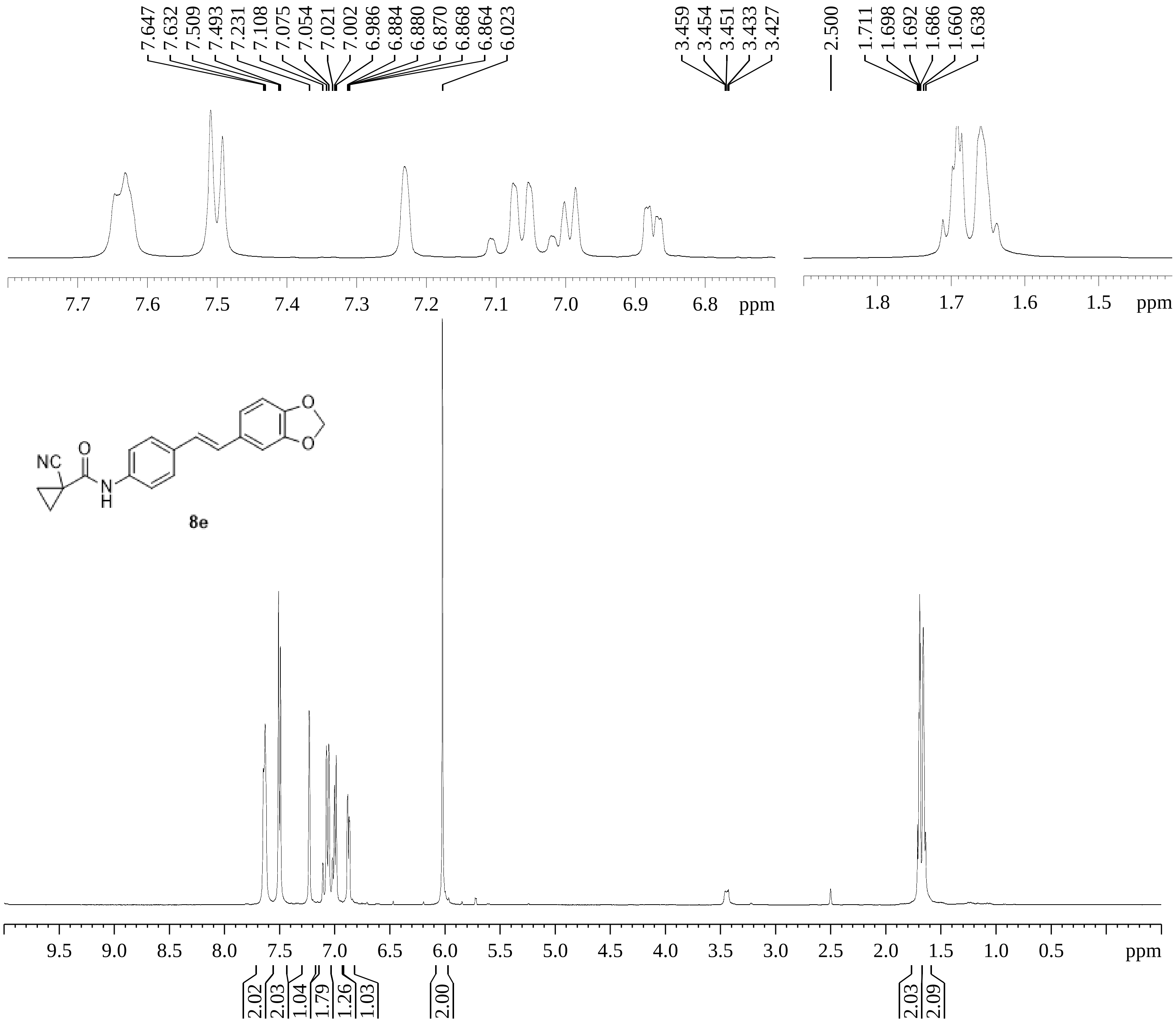

Current Data Parameters
NAME NMR16100058-LSS-50
EXPNO 1
PROCNO 1

F2 - Acquisition Parameters
Date_ 20161017
Time 16.10
INSTRUM spect
PROBHD 5 mm PABBO BB-
PULPROG zg30
TD 65536
SOLVENT DMSO
NS 16
DS 2
SWH 10000.000 Hz
FIDRES 0.152588 Hz
AQ 3.2767999 sec
RG 32.04
DW 50.000 usec
DE 6.50 usec
TE 298.1 K
D1 1.00000000 sec
TD0 1

======== CHANNEL f1 =======
SFO1 500.1330885 MHz
NUC1 1H
P1 11.51 usec
PLW1 19.95299911 W

F2 - Processing parameters
SI 65536
SF 500.1300044 MHz
WDW EM
SSB 0
LB 0.30 Hz
GB 0
PC 1.00

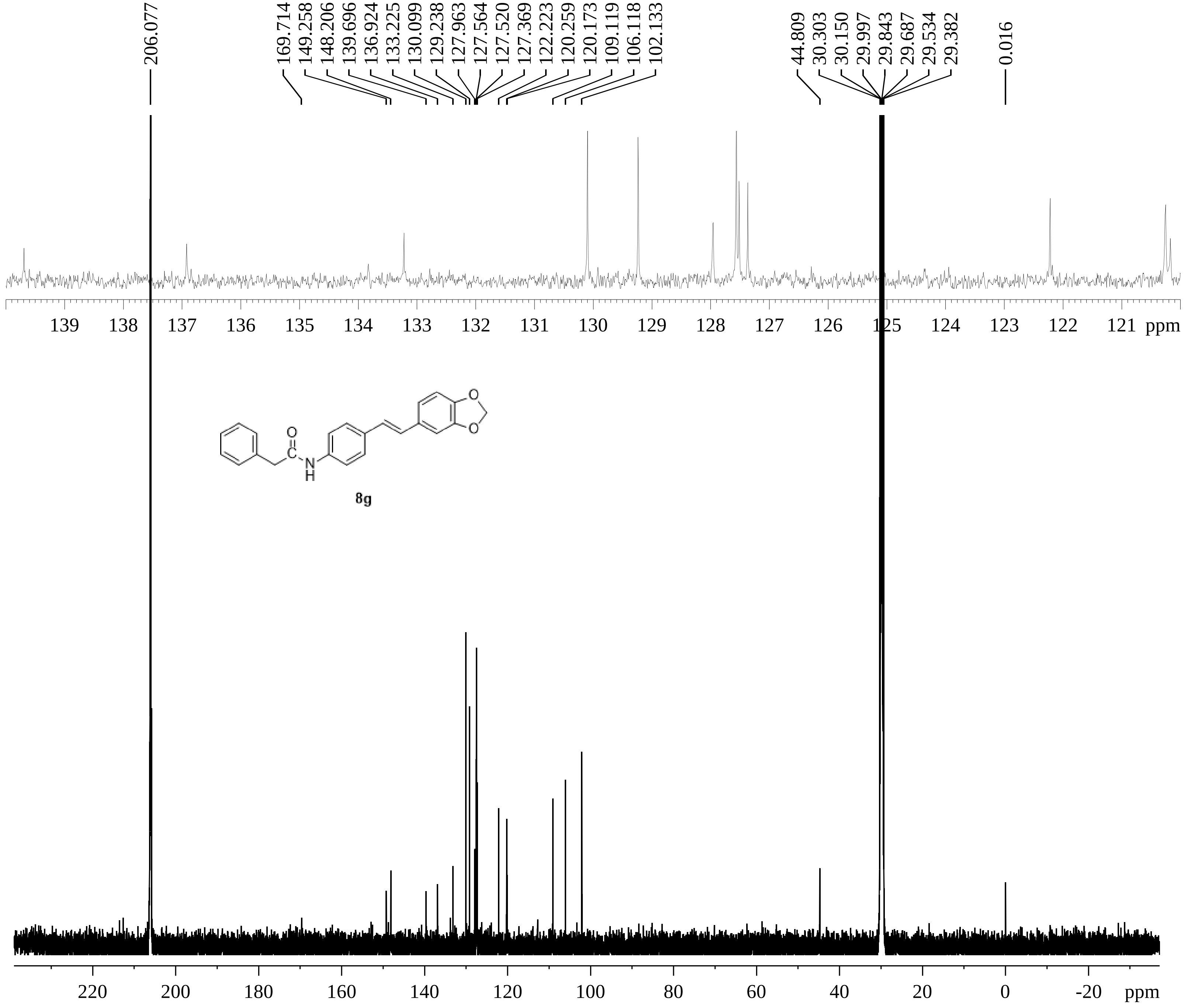

Current Data Parameters
NAME NMR15120053-LSS-16
EXPNO 2
PROCNO 1

F2 - Acquisition Parameters
Date_ 20151204
Time 4.34
INSTRUM spect
PROBHD 5 mm PABBO BB-
PULPROG zgpg30
TD 65536
SOLVENT Acetone
NS 1200
DS 4
SWH 34722.223 Hz
FIDRES 0.529819 Hz
AQ 0.9437184 sec
RG 198.55
DW 14.400 usec
DE 6.50 usec
TE 303.2 K
D1 2.00000000 sec
D11 0.03000000 sec
TD0 1

======== CHANNEL f1 =======
SFO1 125.7703637 MHz
NUC1 13C
P1 8.99 usec
PLW1 125.88999939 W

======== CHANNEL f2 =======
SFO2 500.1320005 MHz
NUC2 1H
CPDPRG[2 waltz16
PCPD2 80.00 usec
PLW2 19.95299911 W
PLW12 0.39528000 W
PLW13 0.25297999 W

F2 - Processing parameters
SI 32768
SF 125.7576707 MHz
WDW EM
SSB 0
LB 1.00 Hz
GB 0
PC 1.40

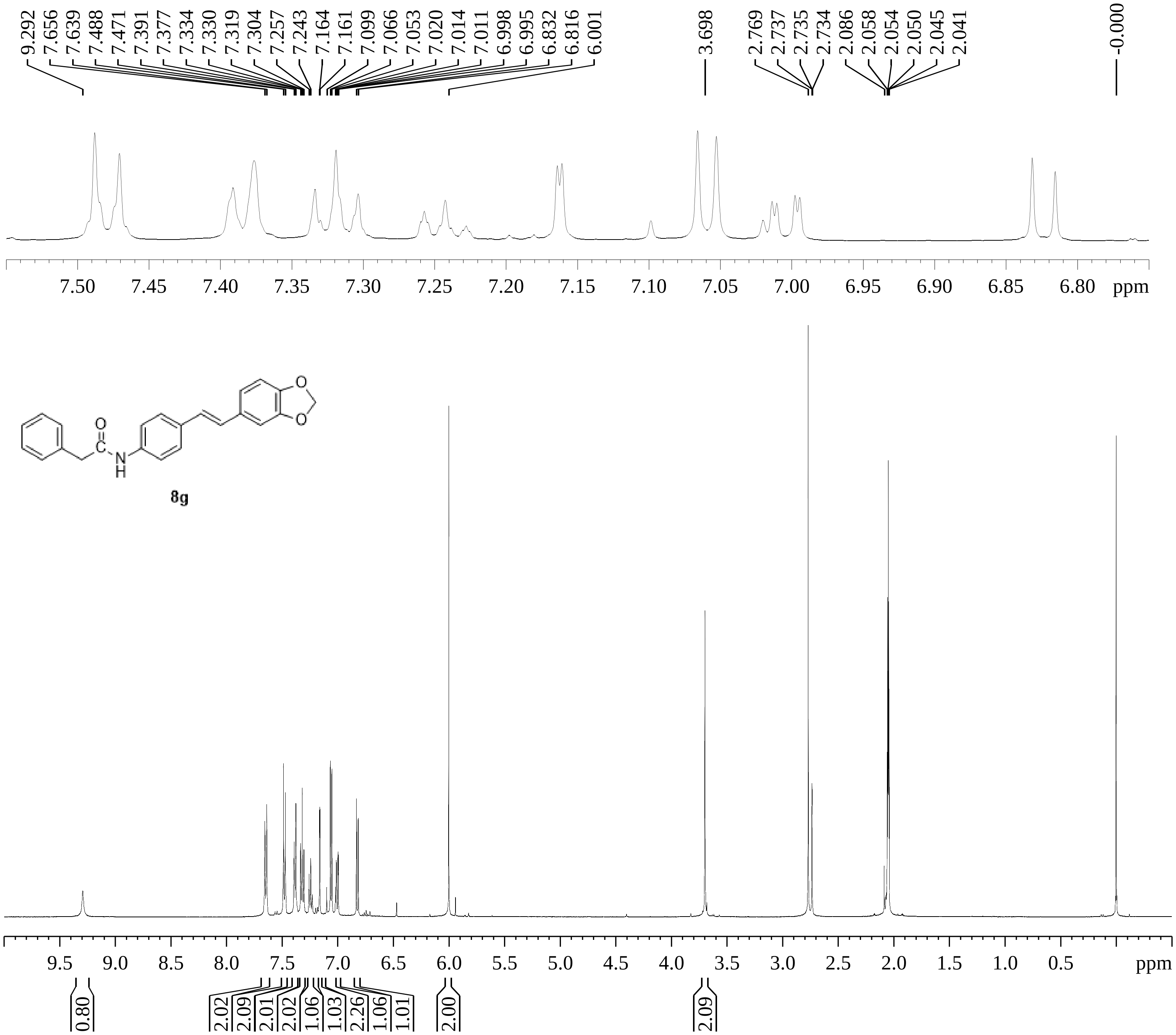

8g

Current Data Parameters
NAME NMR15120053-LSS-16
EXPNO 1
PROCNO 1

F2 - Acquisition Parameters
Date_ 20151202
Time 15.52
INSTRUM spect
PROBHD 5 mm PABBO BB-
PULPROG zg30
TD 65536
SOLVENT Acetone
NS 128
DS 2
SWH 10000.000 Hz
FIDRES 0.152588 Hz
AQ 3.2767999 sec
RG 198.55
DW 50.000 usec
DE 6.50 usec
TE 303.2 K
D1 1.00000000 sec
TD0 1

======== CHANNEL f1 =======
SFO1 500.1330885 MHz
NUC1 1H
P1 11.26 usec
PLW1 19.95299911 W

F2 - Processing parameters
SI 65536
SF 500.1300085 MHz
WDW EM
SSB 0
LB 0.30 Hz
GB 0
PC 1.00

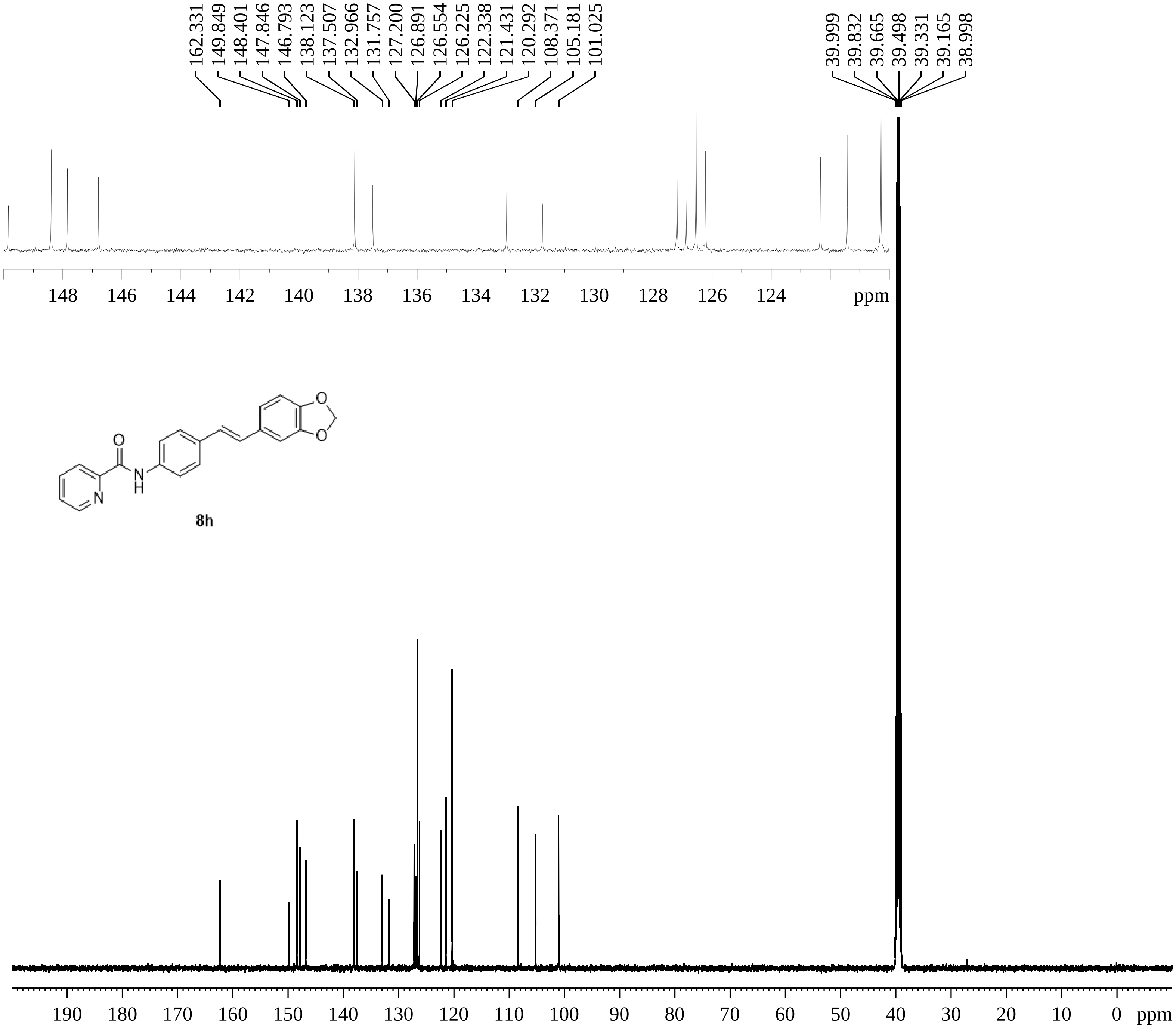

8h

Current Data Parameters
NAME NMR16010803-LSS-20
EXPNO 1
PROCNO 1

F2 - Acquisition Parameters
Date_ 20160130
Time 0.07
INSTRUM spect
PROBHD 5 mm PABBO BB-
PULPROG zgpg30
TD 65536
SOLVENT DMSO
NS 1024
DS 4
SWH 34722.223 Hz
FIDRES 0.529819 Hz
AQ 0.9437184 sec
RG 198.55
DW 14.400 usec
DE 6.50 usec
TE 298.2 K
D1 2.00000000 sec
D11 0.03000000 sec
TD0 1

======== CHANNEL f1 =======
SFO1 125.7703637 MHz
NUC1 13C
P1 8.99 usec
PLW1 125.88999939 W

======== CHANNEL f2 =======
SFO2 500.1320005 MHz
NUC2 1H
CPDPRG[2 waltz16
PCPD2 80.00 usec
PLW2 19.95299911 W
PLW12 0.39528000 W
PLW13 0.25297999 W

F2 - Processing parameters
SI 32768
SF 125.7578517 MHz
WDW EM
SSB 0
LB 1.00 Hz
GB 0
PC 1.40

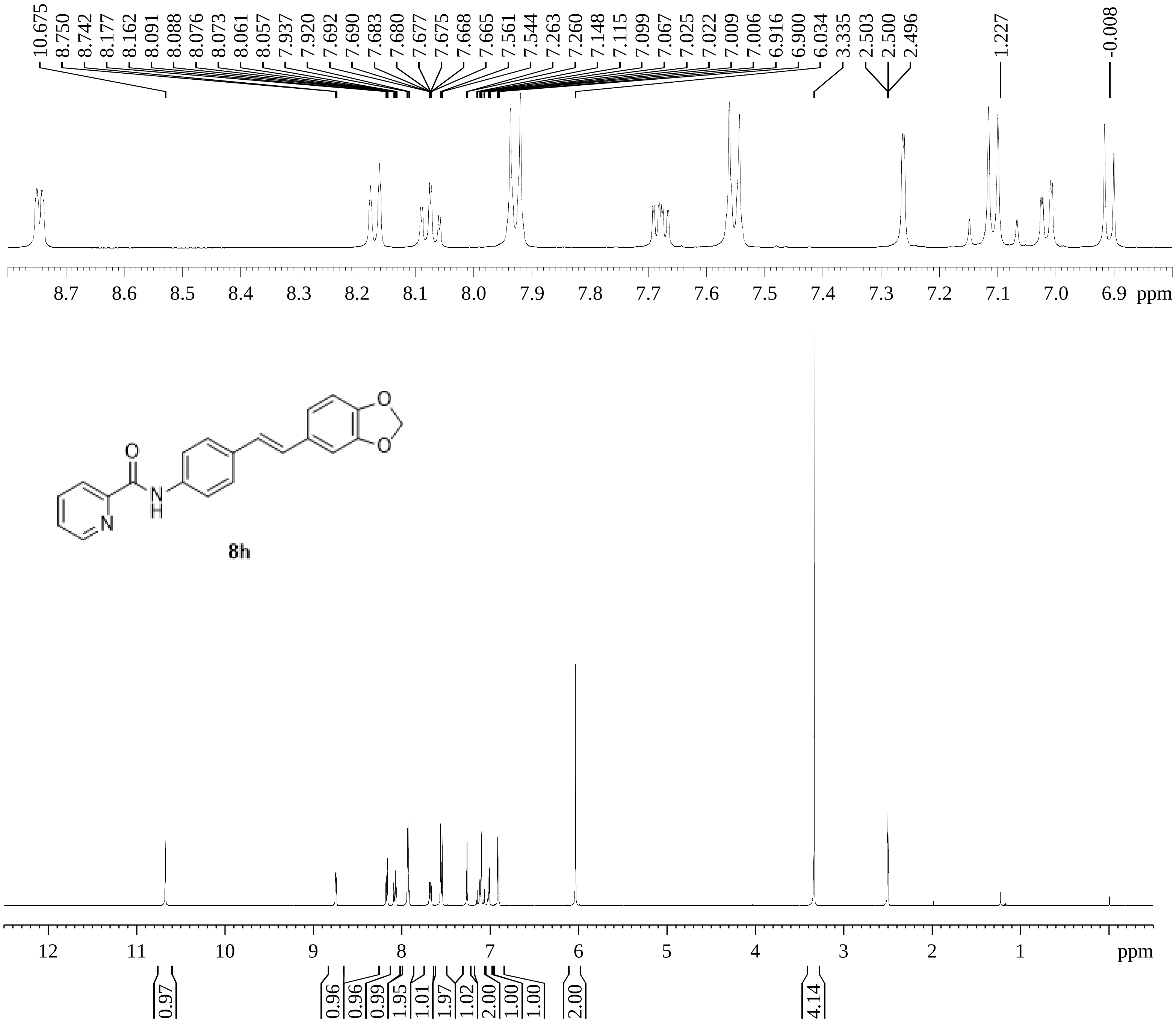

8h

Current Data Parameters
NAME NMR16010314-LSS-20
EXPNO 1
PROCNO 1

F2 - Acquisition Parameters
Date_ 20160121
Time 12.14
INSTRUM spect
PROBHD 5 mm PABBO BB-
PULPROG zg30
TD 65536
SOLVENT DMSO
NS 128
DS 2
SWH 10000.000 Hz
FIDRES 0.152588 Hz
AQ 3.2767999 sec
RG 122.2
DW 50.000 usec
DE 6.50 usec
TE 298.1 K
D1 1.00000000 sec
TD0 1

======== CHANNEL f1 =======
SFO1 500.1330885 MHz
NUC1 1H
P1 11.26 usec
PLW1 19.95299911 W

F2 - Processing parameters
SI 65536
SF 500.1300039 MHz
WDW EM
SSB 0
LB 0.30 Hz
GB 0
PC 1.00

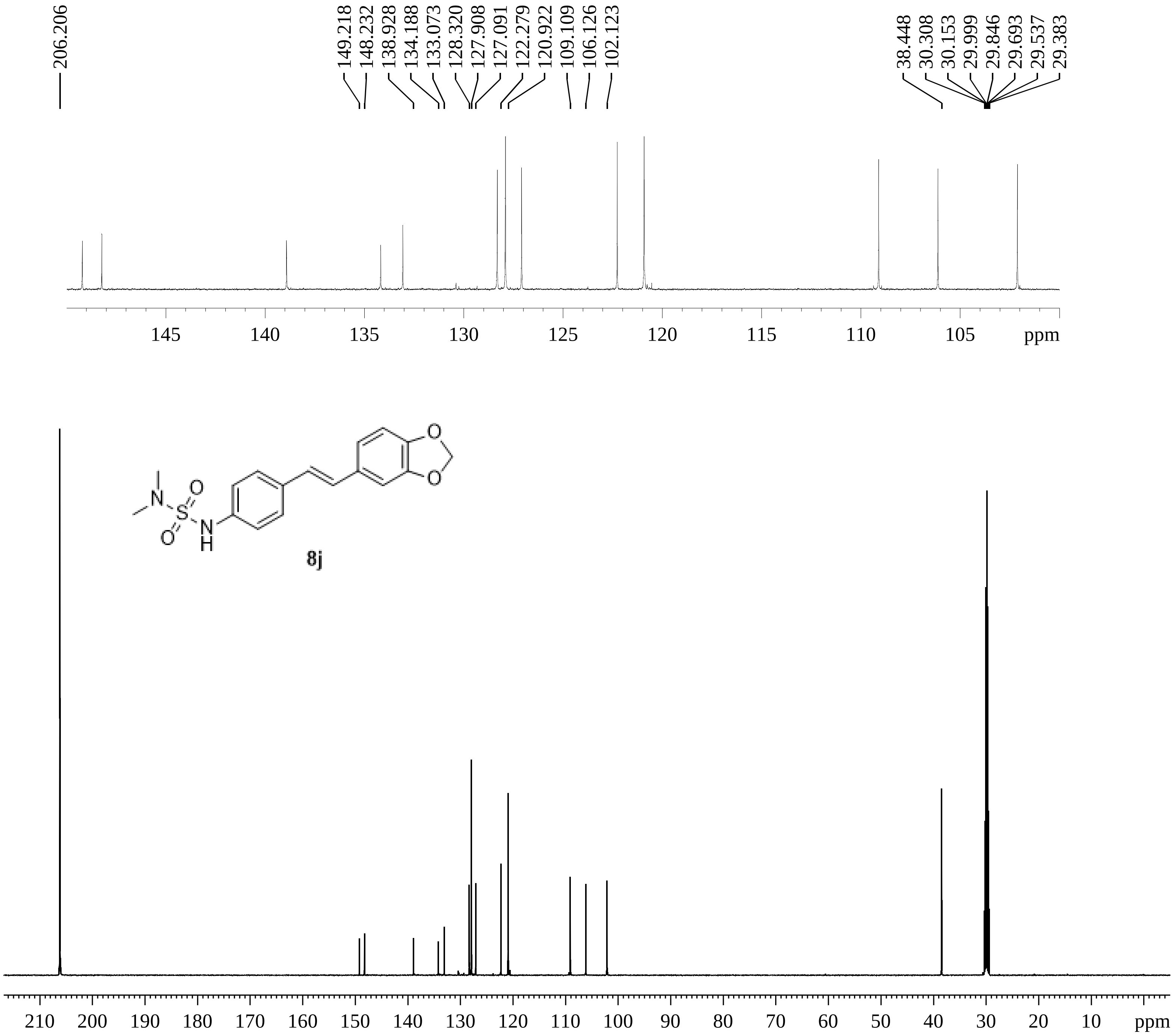

Current Data Parameters
NAME NMR15120810-LSS-17
EXPNO 10
PROCNO 1

F2 - Acquisition Parameters
Date_ 20151223
Time 4.42
INSTRUM spect
PROBHD 5 mm PABBO BB-
PULPROG zgpg30
TD 65536
SOLVENT Acetone
NS 1200
DS 4
SWH 34722.223 Hz
FIDRES 0.529819 Hz
AQ 0.9437184 sec
RG 198.55
DW 14.400 usec
DE 6.50 usec
TE 303.2 K
D1 2.00000000 sec
D11 0.03000000 sec
TD0 1

======== CHANNEL f1 =======
SFO1 125.7703637 MHz
NUC1 13C
P1 8.99 usec
PLW1 125.88999939 W

======== CHANNEL f2 =======
SFO2 500.1320005 MHz
NUC2 1H
CPDPRG[2 waltz16
PCPD2 80.00 usec
PLW2 19.95299911 W
PLW12 0.39528000 W
PLW13 0.25297999 W

F2 - Processing parameters
SI 32768
SF 125.7576771 MHz
WDW EM
SSB 0
LB 1.00 Hz
GB 0
PC 1.40

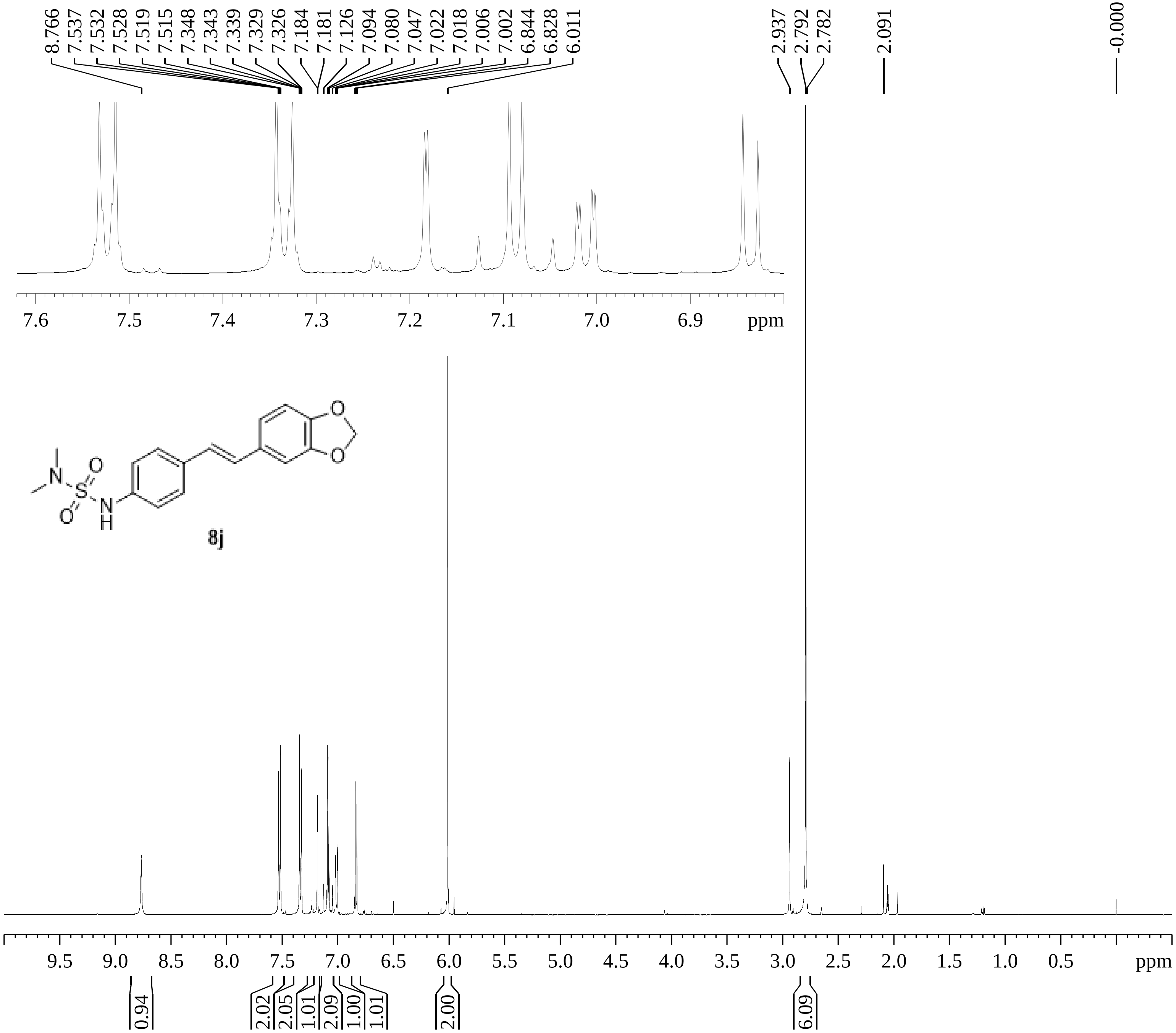

Current Data Parameters
NAME NMR15120810-LSS-17
EXPNO 2
PROCNO 1

F2 - Acquisition Parameters
Date_ 20151222
Time 6.34
INSTRUM spect
PROBHD 5 mm PABBO BB-
PULPROG zg30
TD 65536
SOLVENT Acetone
NS 128
DS 2
SWH 10000.000 Hz
FIDRES 0.152588 Hz
AQ 3.2767999 sec
RG 62.41
DW 50.000 usec
DE 6.50 usec
TE -18.1 K
D1 1.00000000 sec
TD0 1

======== CHANNEL f1 =======
SFO1 500.1330885 MHz
NUC1 1H
P1 11.26 usec
PLW1 19.95299911 W

F2 - Processing parameters
SI 65536
SF 500.1300062 MHz
WDW EM
SSB 0
LB 0.30 Hz
GB 0
PC 1.00

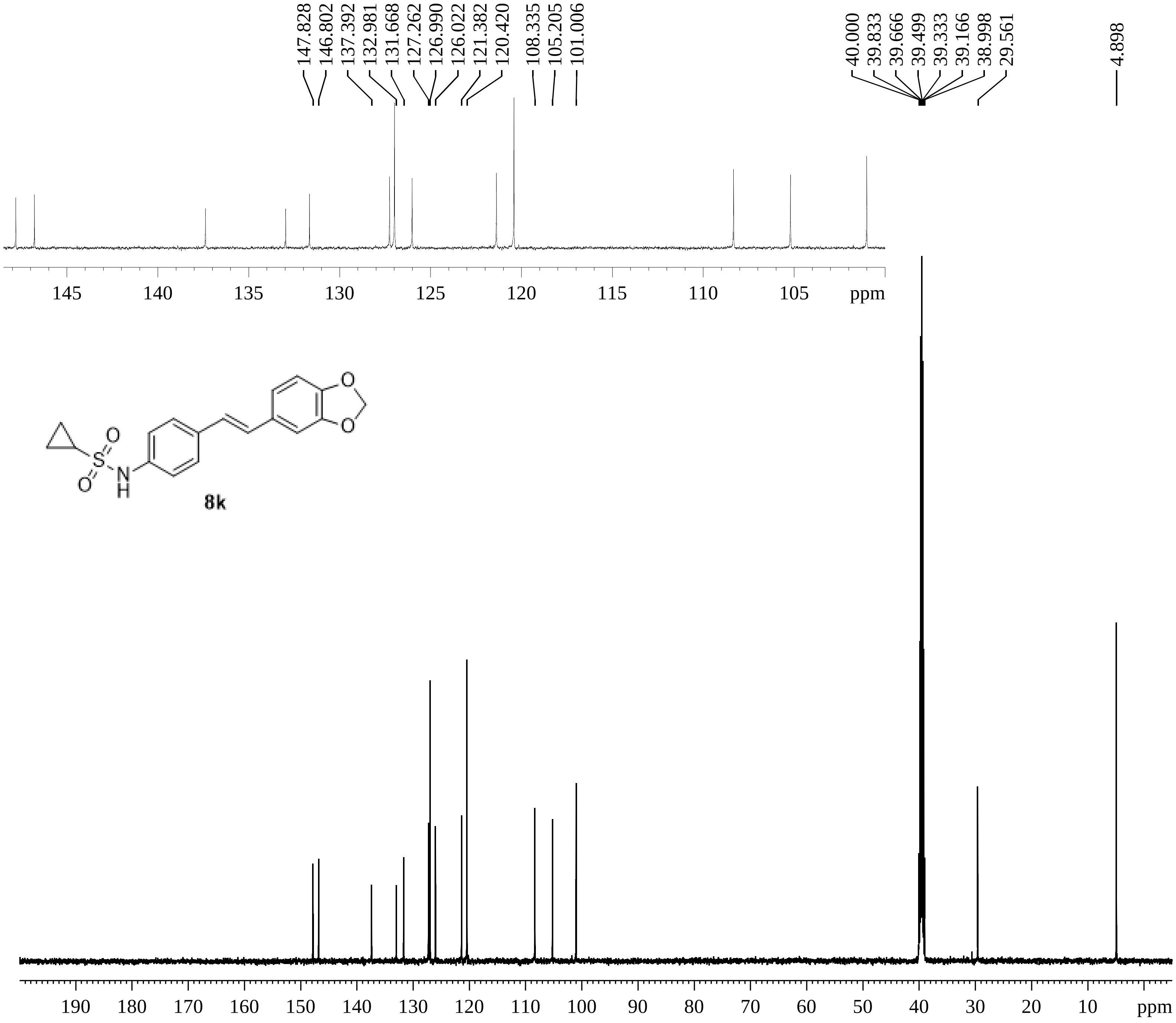

Current Data Parameters
NAME NMR15110717-LSS-13
EXPNO 2
PROCNO 1

F2 - Acquisition Parameters
Date_ 20151123
Time 9.04
INSTRUM spect
PROBHD 5 mm PABBO BB-
PULPROG zgpg30
TD 65536
SOLVENT DMSO
NS 256
DS 4
SWH 34722.223 Hz
FIDRES 0.529819 Hz
AQ 0.9437184 sec
RG 198.55
DW 14.400 usec
DE 6.50 usec
TE 303.2 K
D1 2.00000000 sec
D11 0.03000000 sec
TD0 1

======== CHANNEL f1 =======
SFO1 125.7703637 MHz
NUC1 13C
P1 8.99 usec
PLW1 125.88999939 W

======== CHANNEL f2 =======
SFO2 500.1320005 MHz
NUC2 1H
CPDPRG[2 waltz16
PCPD2 80.00 usec
PLW2 19.95299911 W
PLW12 0.39528000 W
PLW13 0.25297999 W

F2 - Processing parameters
SI 32768
SF 125.7578538 MHz
WDW EM
SSB 0
LB 1.00 Hz
GB 0
PC 1.40

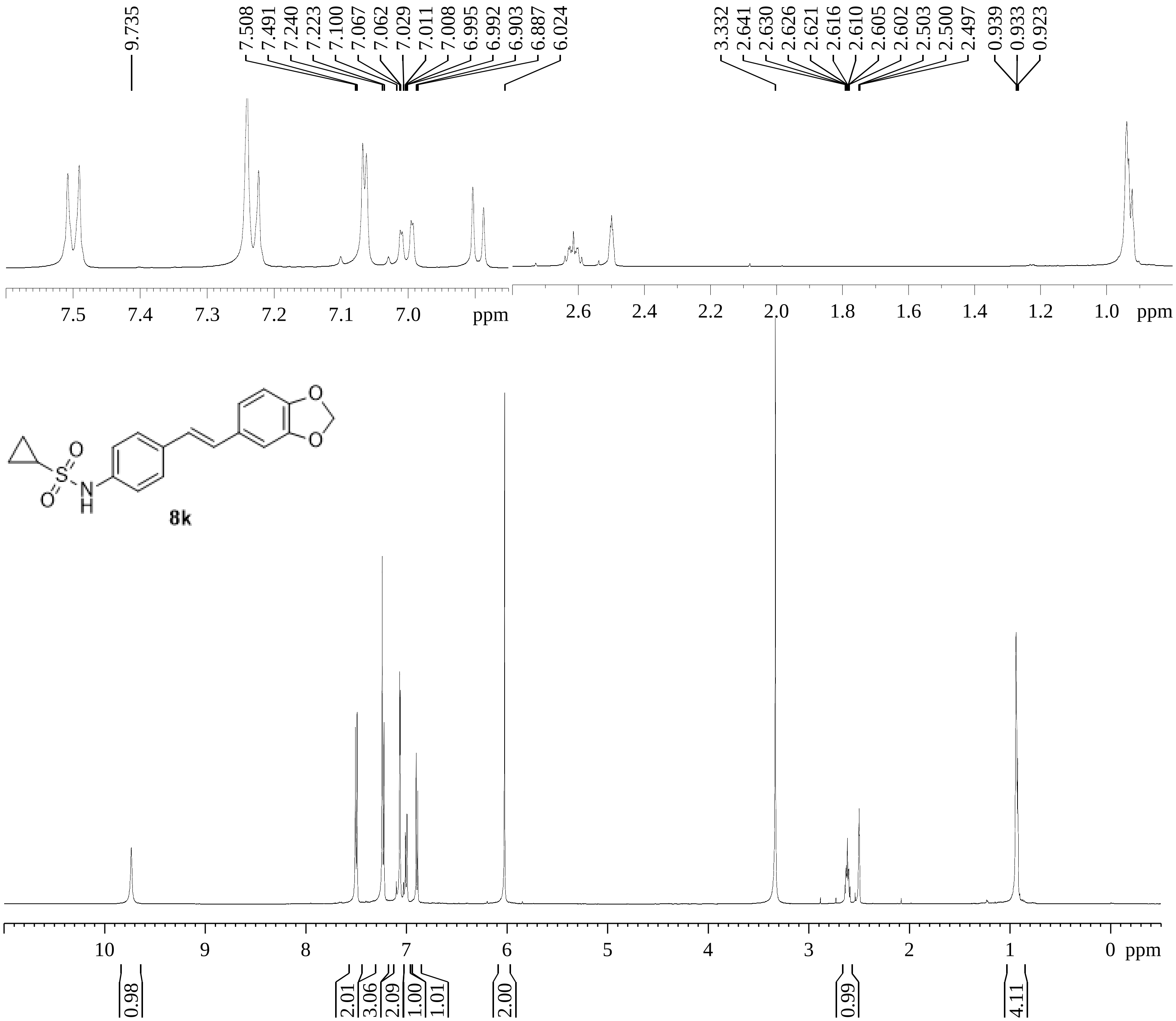

8k

Current Data Parameters
NAME NMR15110717-LSS-13
EXPNO 1
PROCNO 1

F2 - Acquisition Parameters
Date_ 20151120
Time 12.09
INSTRUM spect
PROBHD 5 mm PABBO BB-
PULPROG zg30
TD 65536
SOLVENT DMSO
NS 128
DS 2
SWH 10000.000 Hz
FIDRES 0.152588 Hz
AQ 3.2767999 sec
RG 80.16
DW 50.000 usec
DE 6.50 usec
TE 303.2 K
D1 1.00000000 sec
TD0 1

======== CHANNEL f1 =======
SFO1 500.1330885 MHz
NUC1 1H
P1 11.26 usec
PLW1 19.95299911 W

F2 - Processing parameters
SI 65536
SF 500.1300044 MHz
WDW EM
SSB 0
LB 0.30 Hz
GB 0
PC 1.00

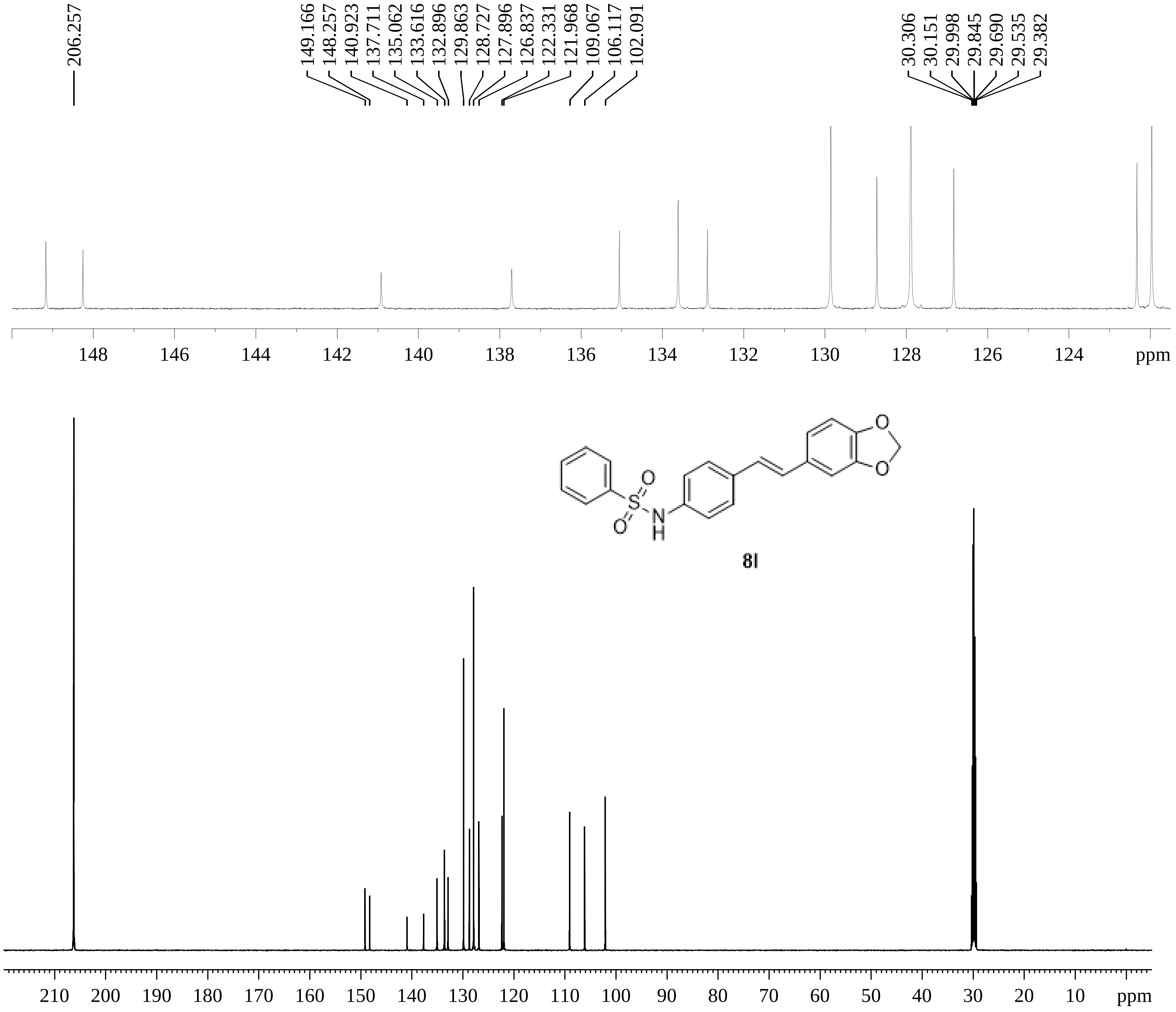

Current Data Parameters
NAME NMR15110068-lss-12
EXPNO 2
PROCNO 1

F2 - Acquisition Parameters
Date_ 20151110
Time 1.19
INSTRUM spect
PROBHD 5 mm PABBO BB-
PULPROG zgpg30
TD 65536
SOLVENT Acetone
NS 1200
DS 4
SWH 34722.223 Hz
FIDRES 0.529819 Hz
AQ 0.9437184 sec
RG 198.55
DW 14.400 usec
DE 6.50 usec
TE 303.1 K
D1 2.00000000 sec
D11 0.03000000 sec
TD0 1

======== CHANNEL f1 =======
SFO1 125.7703637 MHz
NUC1 13C
P1 8.99 usec
PLW1 125.88999939 W

======== CHANNEL f2 =======
SFO2 500.1320005 MHz
NUC2 1H
CPDPRG[2 waltz16
PCPD2 80.00 usec
PLW2 19.95299911 W
PLW12 0.39528000 W
PLW13 0.25297999 W

F2 - Processing parameters
SI 32768
SF 125.7576824 MHz
WDW EM
SSB 0
LB 1.00 Hz
GB 0
PC 1.40

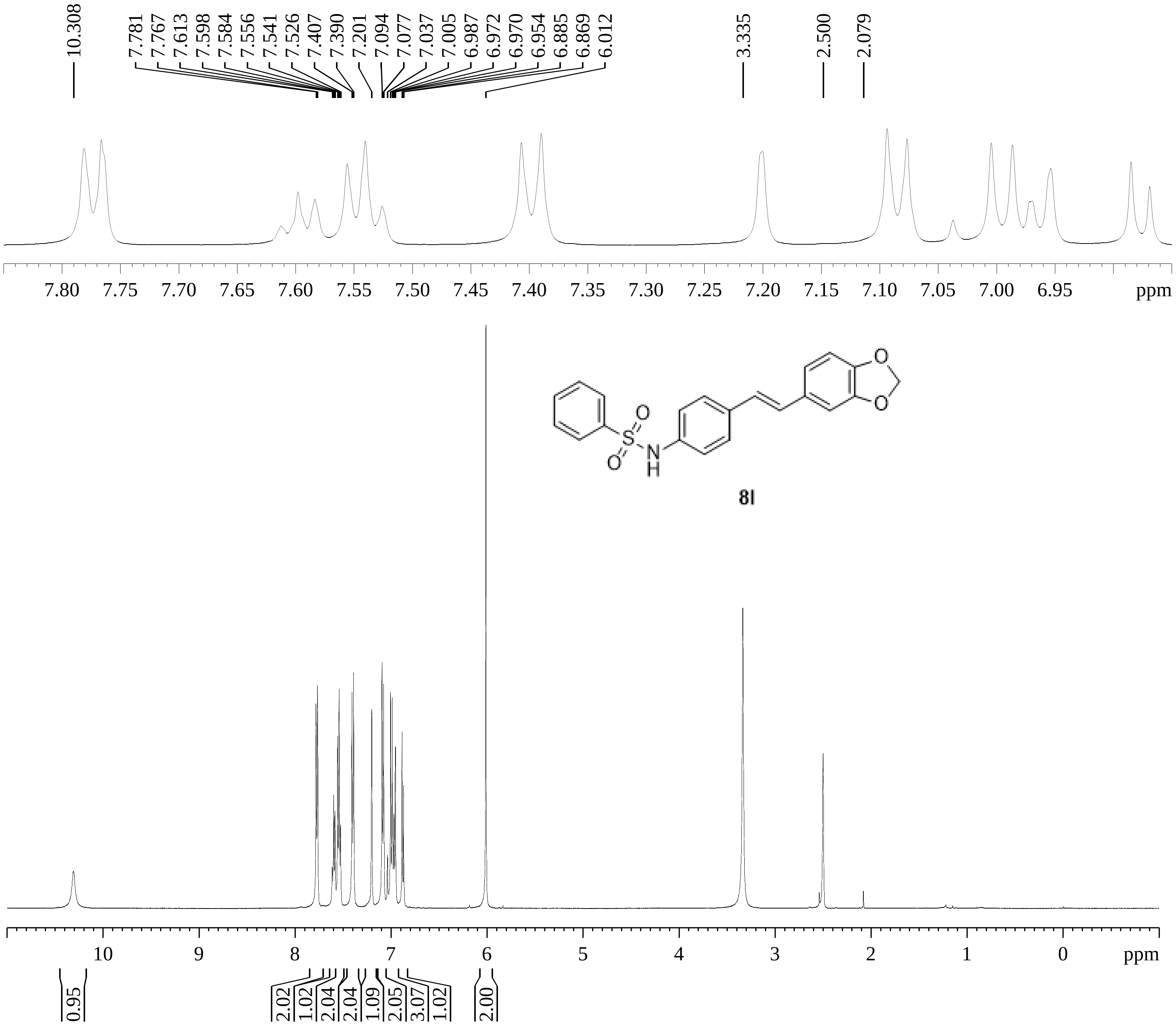

Current Data Parameters
NAME NMR15110068-lss-12
EXPNO 10
PROCNO 1

F2 - Acquisition Parameters
Date_ 20151116
Time 10.25
INSTRUM spect
PROBHD 5 mm PABBO BB-
PULPROG zg30
TD 65536
SOLVENT DMSO
NS 64
DS 2
SWH 10000.000 Hz
FIDRES 0.152588 Hz
AQ 3.2767999 sec
RG 90.46
DW 50.000 usec
DE 6.50 usec
TE 303.1 K
D1 1.00000000 sec
TD0 1

======== CHANNEL f1 =======
SFO1 500.1330885 MHz
NUC1 1H
P1 11.26 usec
PLW1 19.95299911 W

F2 - Processing parameters
SI 65536
SF 500.1300047 MHz
WDW EM
SSB 0
LB 0.10 Hz
GB 0
PC 1.00

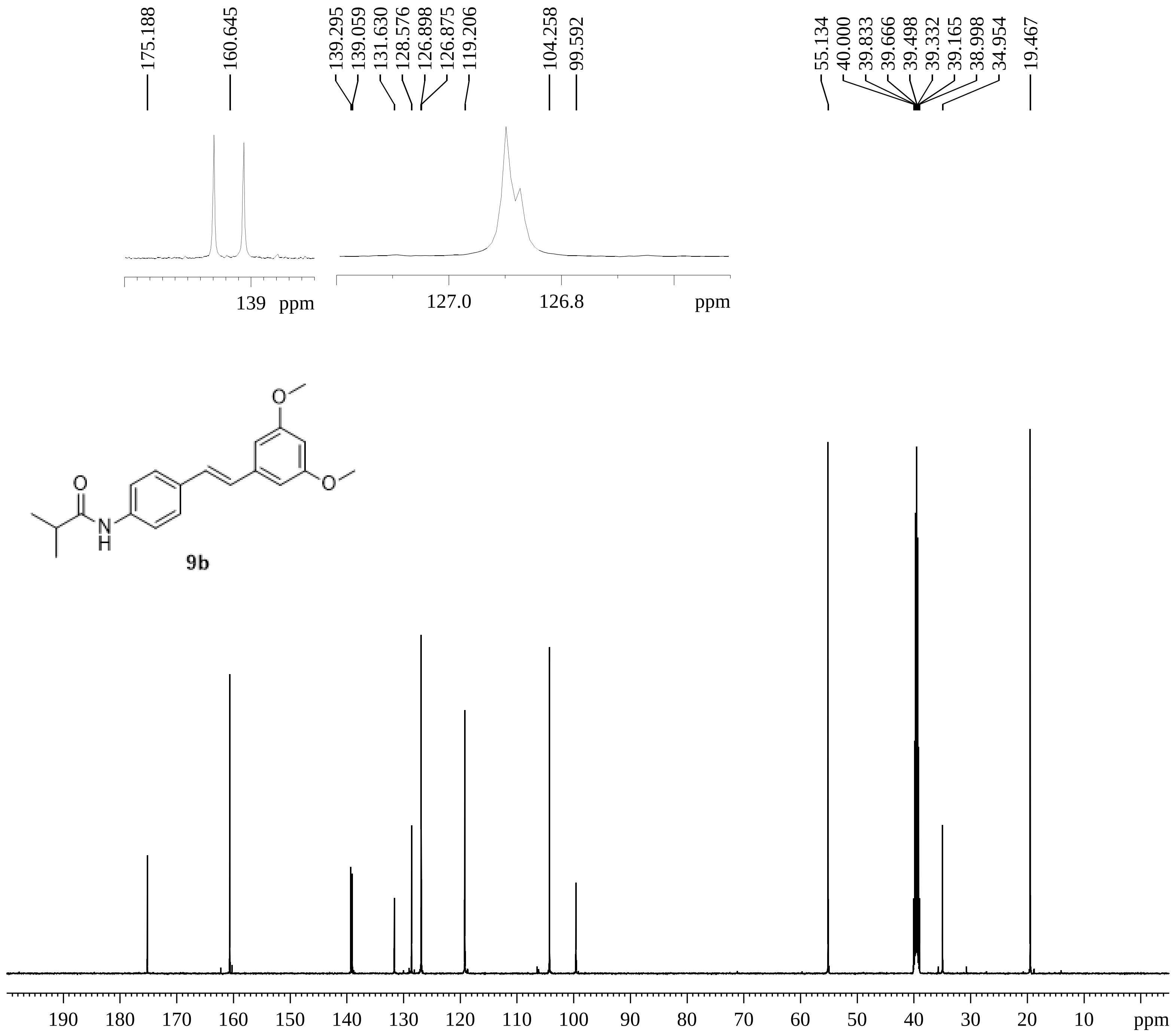

Current Data Parameters
NAME NMR16020154-LSS-21
EXPNO 20
PROCNO 1

F2 - Acquisition Parameters
Date_ 20160227
Time 4.31
INSTRUM spect
PROBHD 5 mm PABBO BB-
PULPROG zgpg30
TD 65536
SOLVENT DMSO
NS 1024
DS 4
SWH 34722.223 Hz
FIDRES 0.529819 Hz
AQ 0.9437184 sec
RG 198.55
DW 14.400 usec
DE 6.50 usec
TE 298.1 K
D1 2.00000000 sec
D11 0.03000000 sec
TD0 1

======== CHANNEL f1 =======
SFO1 125.7703637 MHz
NUC1 13C
P1 8.99 usec
PLW1 125.88999939 W

======== CHANNEL f2 =======
SFO2 500.1320005 MHz
NUC2 1H
CPDPRG[2 waltz16
PCPD2 80.00 usec
PLW2 19.95299911 W
PLW12 0.39528000 W
PLW13 0.25297999 W

F2 - Processing parameters
SI 32768
SF 125.7578506 MHz
WDW EM
SSB 0
LB 1.00 Hz
GB 0
PC 1.40

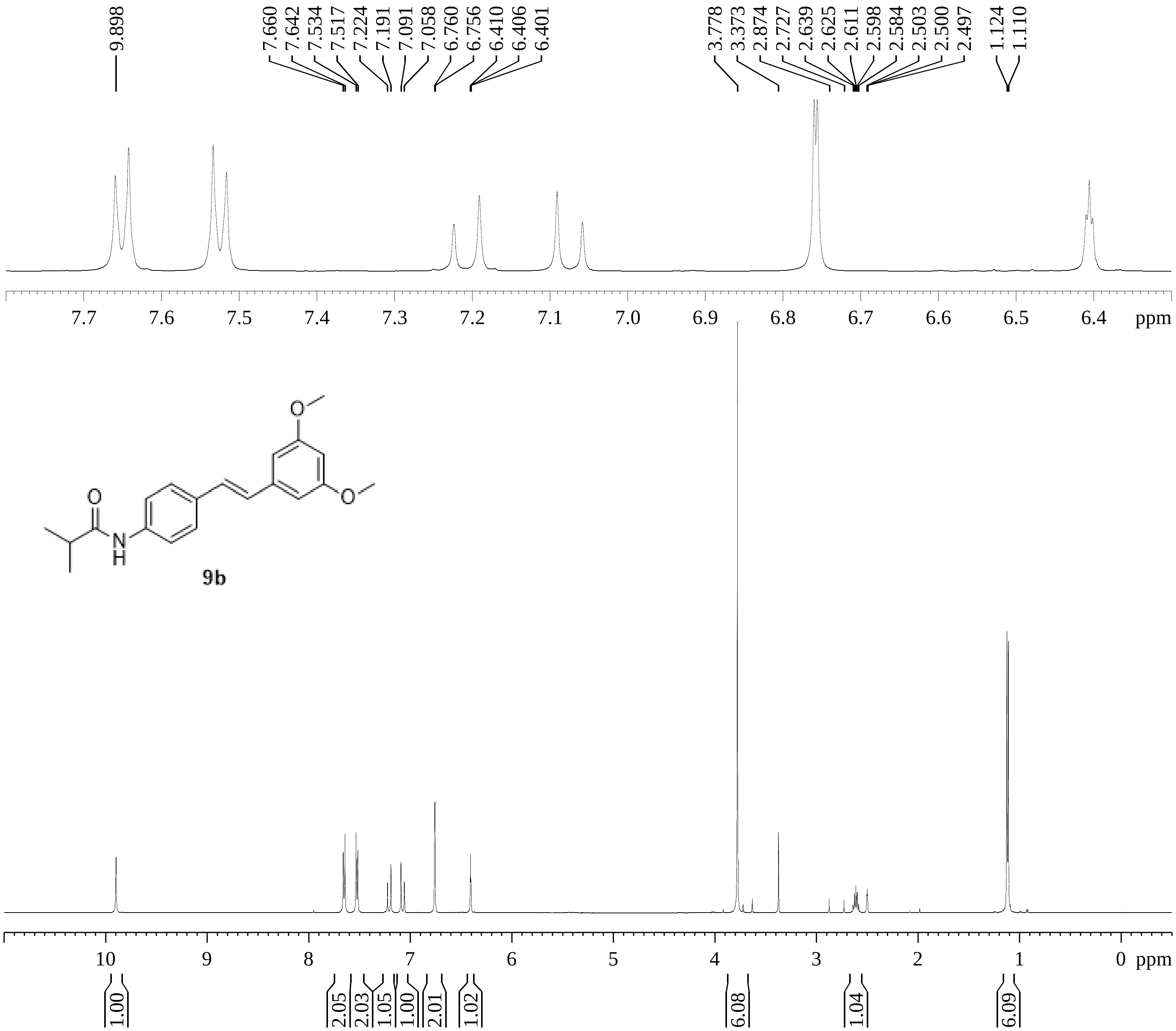

9b

Current Data Parameters
NAME NMR16020154-LSS-21
EXPNO 10
PROCNO 1

F2 - Acquisition Parameters
Date_ 20160225
Time 11.51
INSTRUM spect
PROBHD 5 mm PABBO BB-
PULPROG zg30
TD 65536
SOLVENT DMSO
NS 128
DS 2
SWH 10000.000 Hz
FIDRES 0.152588 Hz
AQ 3.2767999 sec
RG 32.04
DW 50.000 usec
DE 6.50 usec
TE 298.1 K
D1 1.00000000 sec
TD0 1

======== CHANNEL f1 =======
SFO1 500.1330885 MHz
NUC1 1H
P1 11.26 usec
PLW1 19.95299911 W

F2 - Processing parameters
SI 65536
SF 500.1300038 MHz
WDW EM
SSB 0
LB 0.30 Hz
GB 0
PC 1.00

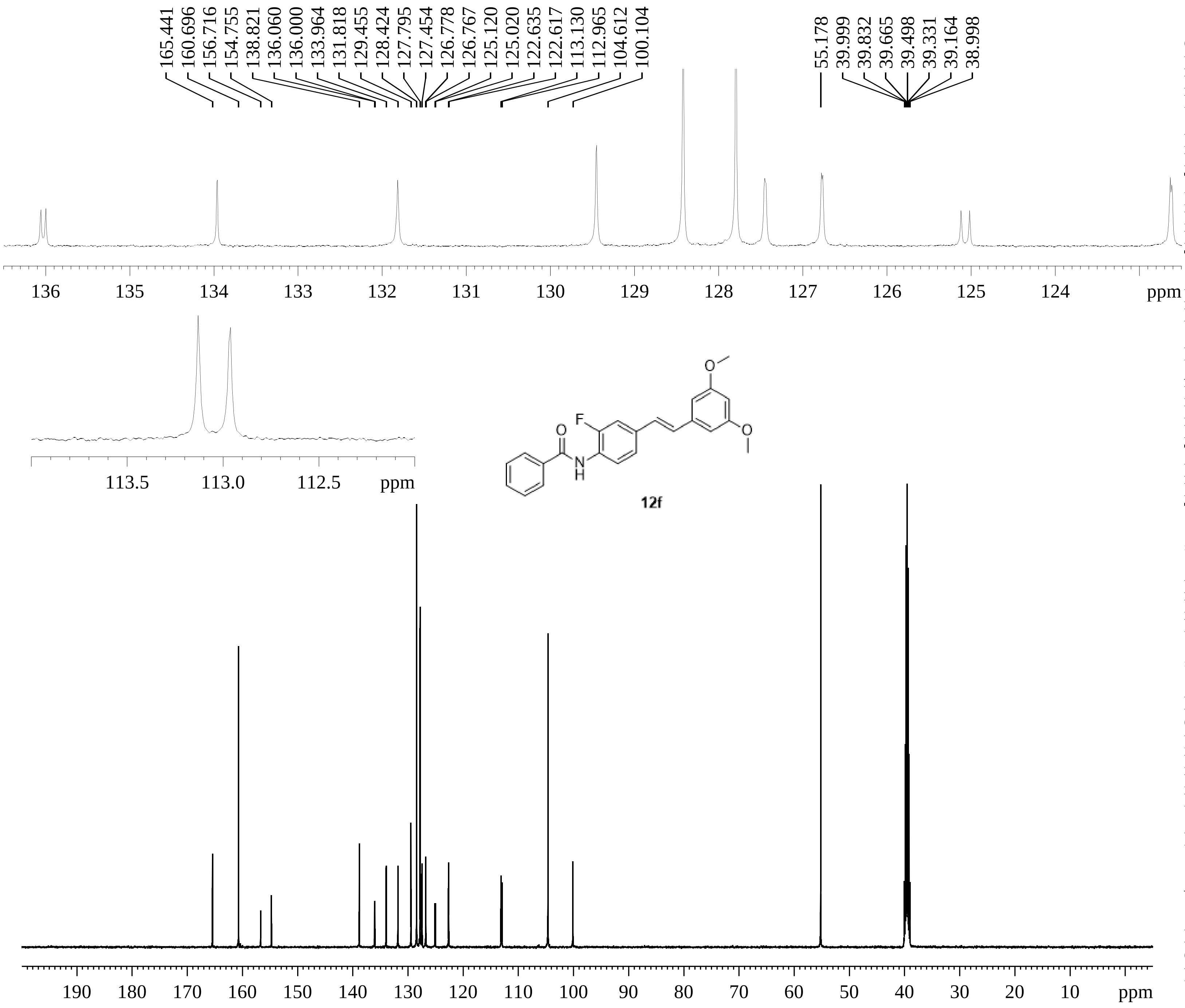

12f

Current Data Parameters
NAME NMR16031134-LSS-31
EXPNO 2
PROCNO 1

F2 - Acquisition Parameters
Date_ 20160330
Time 8.15
INSTRUM spect
PROBHD 5 mm PABBO BB-
PULPROG zgpg30
TD 65536
SOLVENT DMSO
NS 600
DS 4
SWH 34722.223 Hz
FIDRES 0.529819 Hz
AQ 0.9437184 sec
RG 198.55
DW 14.400 usec
DE 6.50 usec
TE 298.2 K
D1 2.00000000 sec
D11 0.03000000 sec
TD0 1

======== CHANNEL f1 =======
SFO1 125.7703637 MHz
NUC1 13C
P1 8.99 usec
PLW1 125.88999939 W

======== CHANNEL f2 =======
SFO2 500.1320005 MHz
NUC2 1H
CPDPRG[2 waltz16
PCPD2 80.00 usec
PLW2 19.95299911 W
PLW12 0.39528000 W
PLW13 0.25297999 W

F2 - Processing parameters
SI 32768
SF 125.7578496 MHz
WDW EM
SSB 0
LB 1.00 Hz
GB 0
PC 1.40

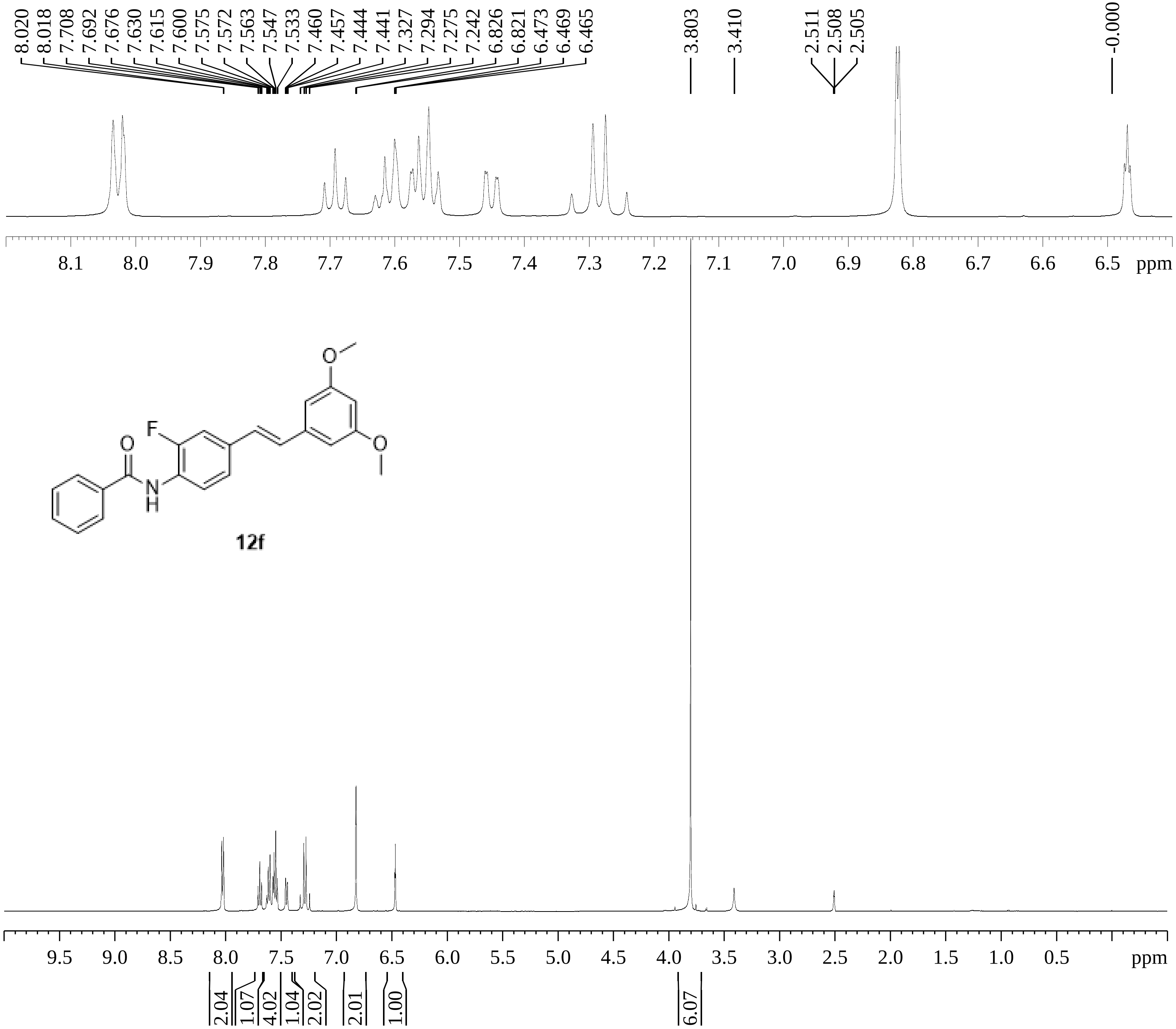

12f

Current Data Parameters
NAME NMR16031134-LSS-31
EXPNO 1
PROCNO 1

F2 - Acquisition Parameters
Date_ 20160328
Time 11.47
INSTRUM spect
PROBHD 5 mm PABBO BB-
PULPROG zg30
TD 65536
SOLVENT DMSO
NS 64
DS 2
SWH 10000.000 Hz
FIDRES 0.152588 Hz
AQ 3.2767999 sec
RG 32.04
DW 50.000 usec
DE 6.50 usec
TE 298.1 K
D1 1.00000000 sec
TD0 1

======== CHANNEL f1 =======
SFO1 500.1330885 MHz
NUC1 1H
P1 11.26 usec
PLW1 19.95299911 W

F2 - Processing parameters
SI 65536
SF 500.1300004 MHz
WDW EM
SSB 0
LB 0.30 Hz
GB 0
PC 1.00

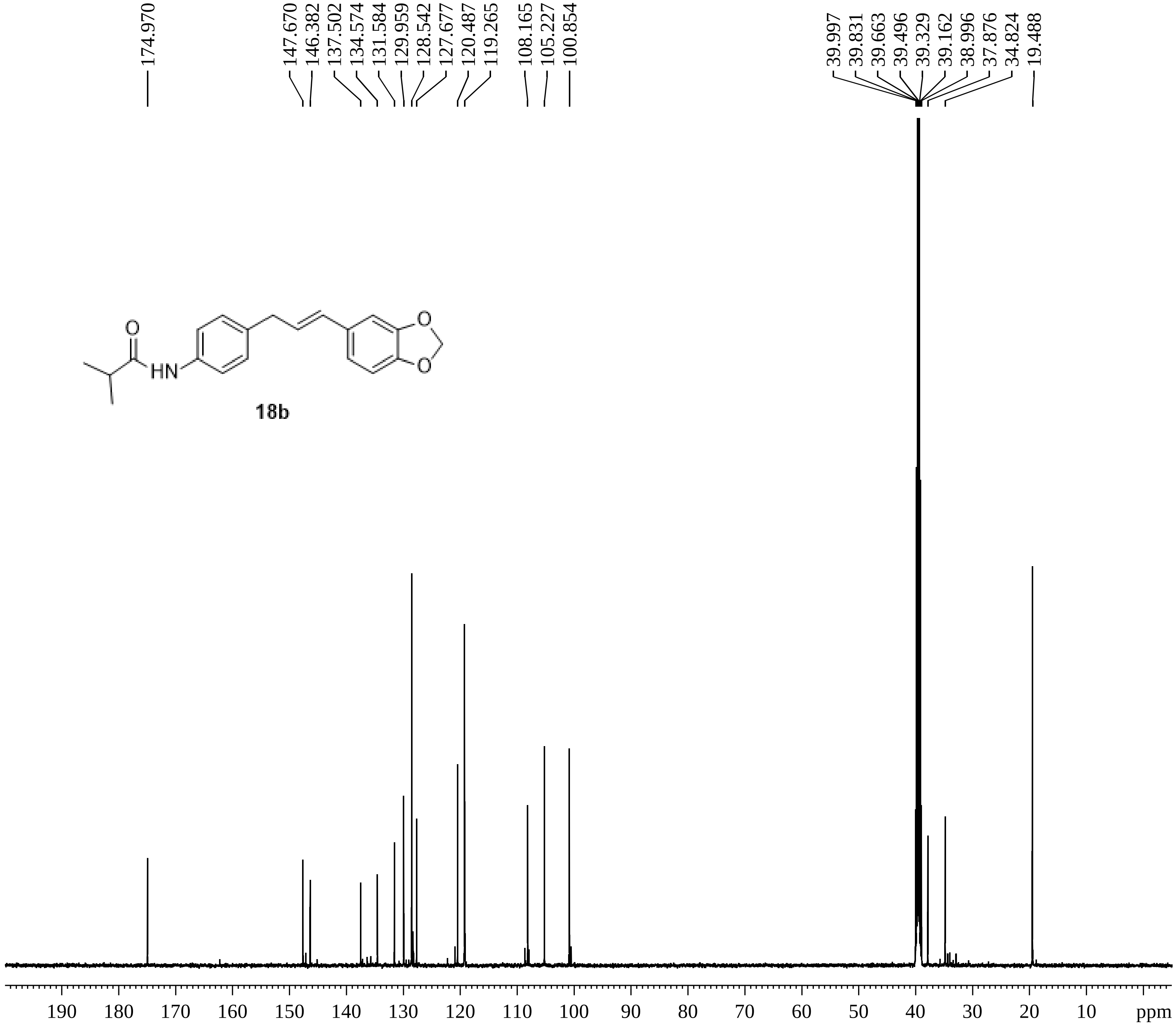

18b

Current Data Parameters
NAME NMR16020155-LSS-22
EXPNO 20
PROCNO 1

F2 - Acquisition Parameters
Date_ 20160227
Time 5.26
INSTRUM spect
PROBHD 5 mm PABBO BB-
PULPROG zgpg30
TD 65536
SOLVENT DMSO
NS 1024
DS 4
SWH 34722.223 Hz
FIDRES 0.529819 Hz
AQ 0.9437184 sec
RG 198.55
DW 14.400 usec
DE 6.50 usec
TE 298.1 K
D1 2.00000000 sec
D11 0.03000000 sec
TD0 1

======== CHANNEL f1 =======
SFO1 125.7703637 MHz
NUC1 13C
P1 8.99 usec
PLW1 125.88999939 W

======== CHANNEL f2 =======
SFO2 500.1320005 MHz
NUC2 1H
CPDPRG[2 waltz16
PCPD2 80.00 usec
PLW2 19.95299911 W
PLW12 0.39528000 W
PLW13 0.25297999 W

F2 - Processing parameters
SI 32768
SF 125.7578517 MHz
WDW EM
SSB 0
LB 1.00 Hz
GB 0
PC 1.40

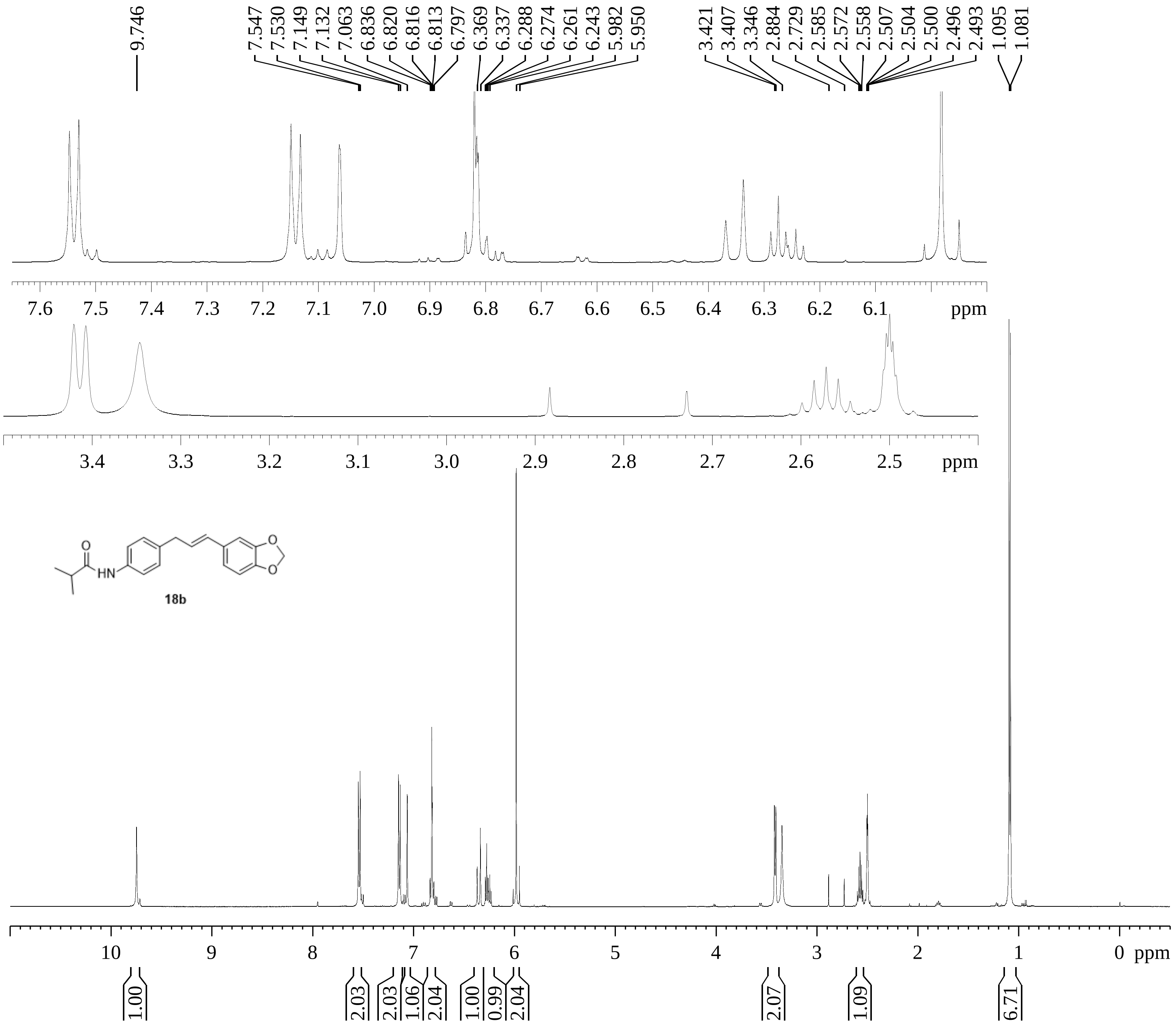

18b

Current Data Parameters
NAME NMR16020155-LSS-22
EXPNO 10
PROCNO 1

F2 - Acquisition Parameters
Date_ 20160225
Time 12.03
INSTRUM spect
PROBHD 5 mm PABBO BB-
PULPROG zg30
TD 65536
SOLVENT DMSO
NS 128
DS 2
SWH 10000.000 Hz
FIDRES 0.152588 Hz
AQ 3.2767999 sec
RG 80.16
DW 50.000 usec
DE 6.50 usec
TE 298.1 K
D1 1.00000000 sec
TD0 1

======== CHANNEL f1 =======
SFO1 500.1330885 MHz
NUC1 1H
P1 11.26 usec
PLW1 19.95299911 W

F2 - Processing parameters
SI 65536
SF 500.1300039 MHz
WDW EM
SSB 0
LB 0.30 Hz
GB 0
PC 1.00

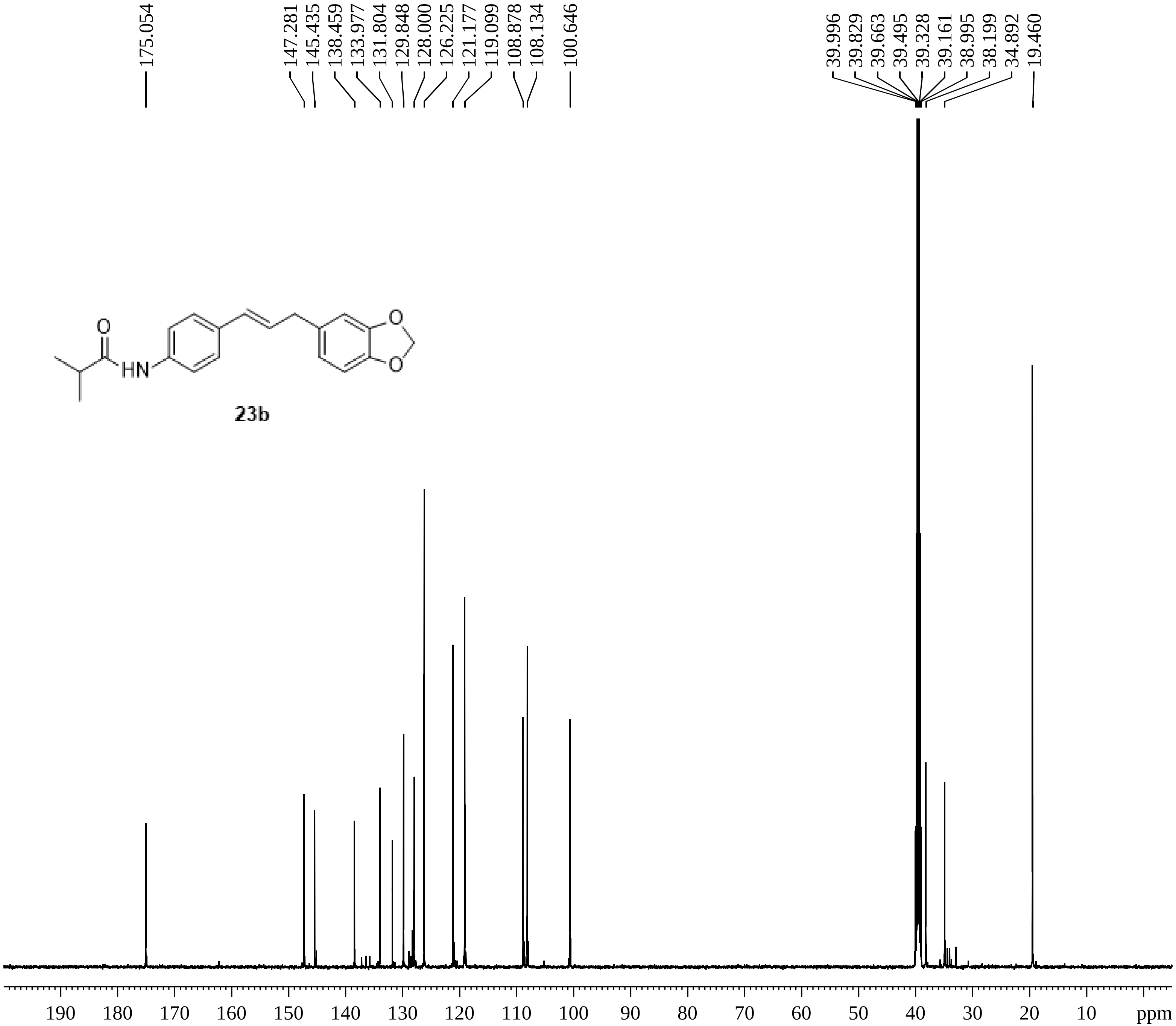

23b

Current Data Parameters
NAME NMR16020156-LSS-23
EXPNO 10
PROCNO 1

F2 - Acquisition Parameters
Date_ 20160227
Time 6.20
INSTRUM spect
PROBHD 5 mm PABBO BB-
PULPROG zgpg30
TD 65536
SOLVENT DMSO
NS 1024
DS 4
SWH 34722.223 Hz
FIDRES 0.529819 Hz
AQ 0.9437184 sec
RG 198.55
DW 14.400 usec
DE 6.50 usec
TE 298.2 K
D1 2.00000000 sec
D11 0.03000000 sec
TD0 1

======== CHANNEL f1 =======
SFO1 125.7703637 MHz
NUC1 13C
P1 8.99 usec
PLW1 125.88999939 W

======== CHANNEL f2 =======
SFO2 500.1320005 MHz
NUC2 1H
CPDPRG[2 waltz16
PCPD2 80.00 usec
PLW2 19.95299911 W
PLW12 0.39528000 W
PLW13 0.25297999 W

F2 - Processing parameters
SI 32768
SF 125.7578517 MHz
WDW EM
SSB 0
LB 1.00 Hz
GB 0
PC 1.40

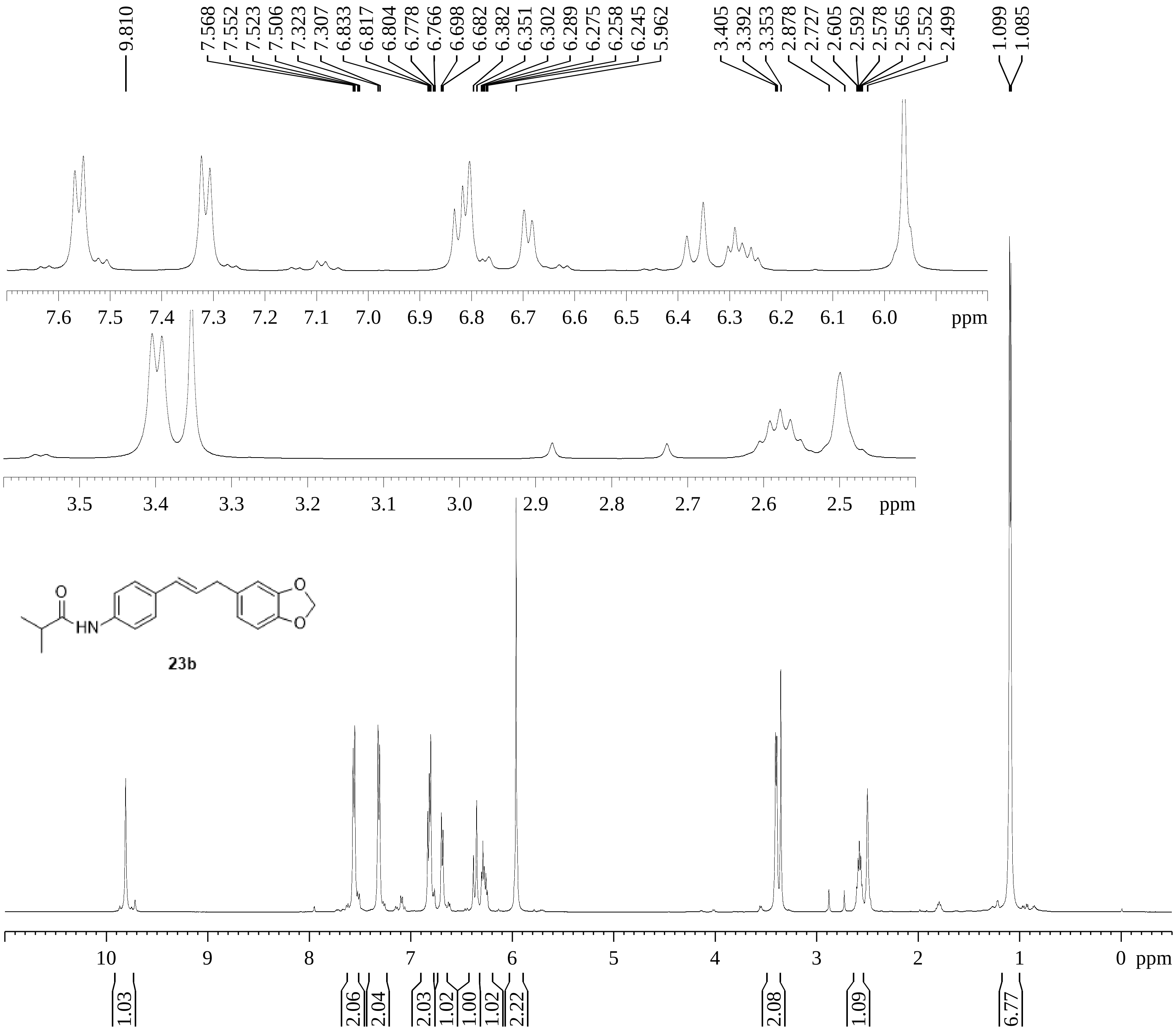

Current Data Parameters
NAME NMR16020156-LSS-23
EXPNO 20
PROCNO 1

F2 - Acquisition Parameters
Date_ 20160225
Time 12.15
INSTRUM spect
PROBHD 5 mm PABBO BB-
PULPROG zg30
TD 65536
SOLVENT DMSO
NS 128
DS 2
SWH 10000.000 Hz
FIDRES 0.152588 Hz
AQ 3.2767999 sec
RG 56.83
DW 50.000 usec
DE 6.50 usec
TE 298.1 K
D1 1.00000000 sec
TD0 1

======== CHANNEL f1 =======
SFO1 500.1330885 MHz
NUC1 1H
P1 11.26 usec
PLW1 19.95299911 W

F2 - Processing parameters
SI 65536
SF 500.1300043 MHz
WDW EM
SSB 0
LB 0.30 Hz
GB 0
PC 1.00

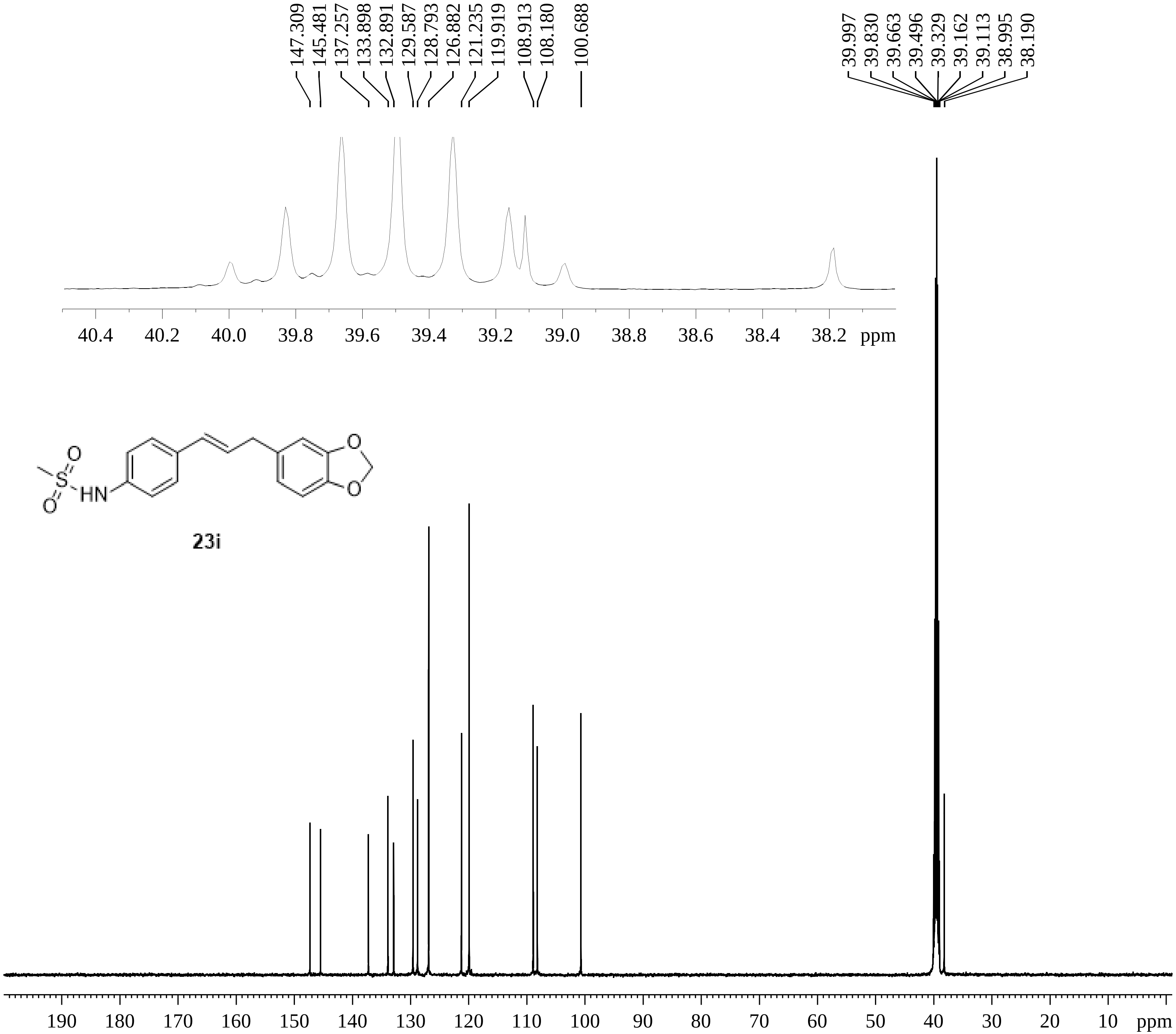

23i

Current Data Parameters
NAME NMR16040130-LSS-32-C
EXPNO 1
PROCNO 1

F2 - Acquisition Parameters
Date_ 20160407
Time 5.16
INSTRUM spect
PROBHD 5 mm PABBO BB-
PULPROG zgpg30
TD 65536
SOLVENT DMSO
NS 1024
DS 4
SWH 32894.738 Hz
FIDRES 0.501934 Hz
AQ 0.9961472 sec
RG 198.55
DW 15.200 usec
DE 6.50 usec
TE 298.1 K
D1 2.00000000 sec
D11 0.03000000 sec
TD0 1

======== CHANNEL f1 =======
SFO1 125.7703637 MHz
NUC1 13C
P1 8.99 usec
PLW1 125.88999939 W

======== CHANNEL f2 =======
SFO2 500.1320005 MHz
NUC2 1H
CPDPRG[2 waltz16
PCPD2 80.00 usec
PLW2 19.95299911 W
PLW12 0.39528000 W
PLW13 0.25297999 W

F2 - Processing parameters
SI 32768
SF 125.7578482 MHz
WDW EM
SSB 0
LB 1.00 Hz
GB 0
PC 1.40

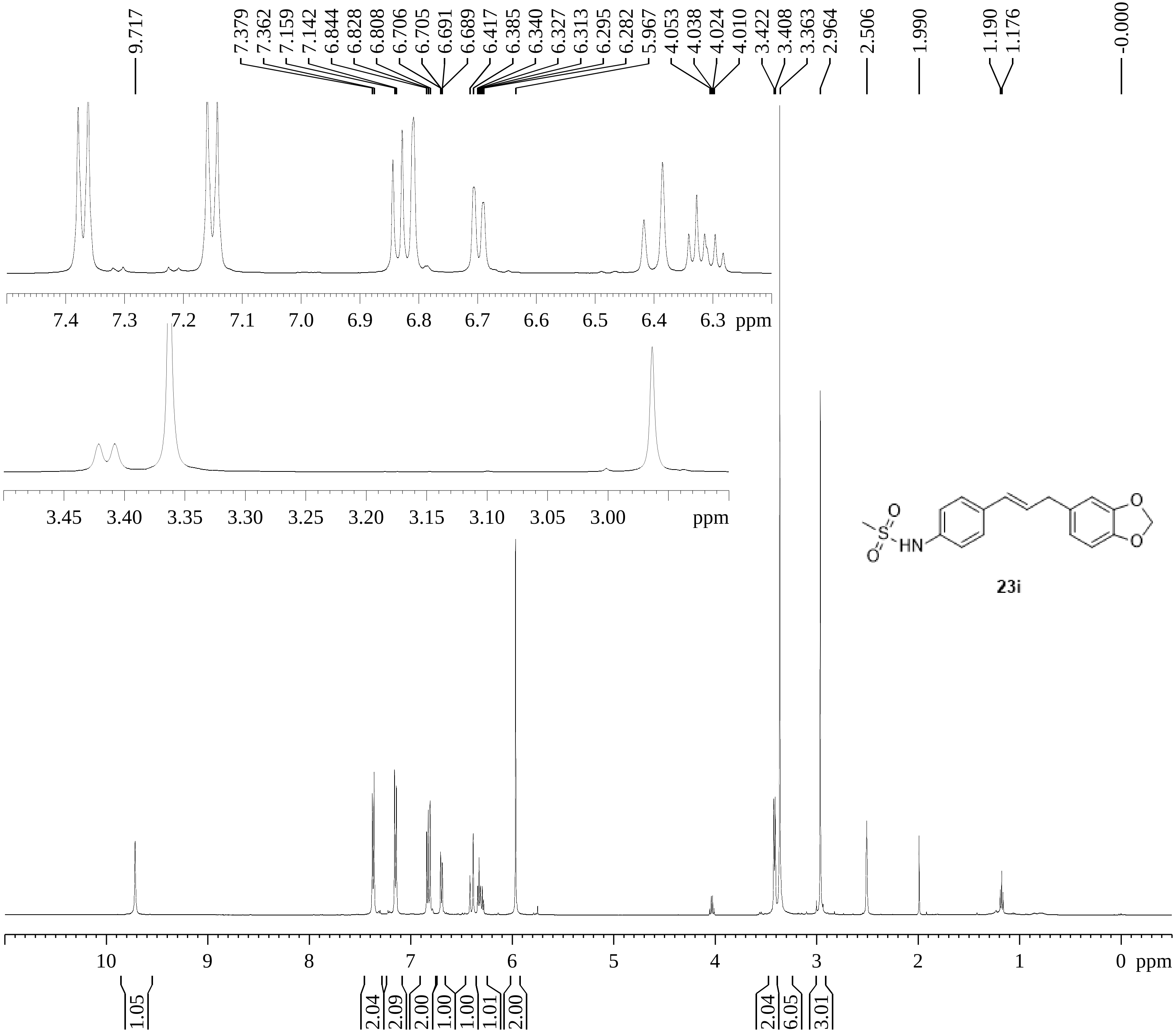

23i

Current Data Parameters
NAME NMR16031257-LSS-32
EXPNO 1
PROCNO 1

F2 - Acquisition Parameters
Date_ 20160330
Time 16.39
INSTRUM spect
PROBHD 5 mm PABBO BB-
PULPROG zg30
TD 65536
SOLVENT DMSO
NS 32
DS 2
SWH 10000.000 Hz
FIDRES 0.152588 Hz
AQ 3.2767999 sec
RG 72.71
DW 50.000 usec
DE 6.50 usec
TE 298.2 K
D1 1.00000000 sec
TD0 1

======== CHANNEL f1 ========
SFO1 500.1330885 MHz
NUC1 1H
P1 11.26 usec
PLW1 19.95299911 W

F2 - Processing parameters
SI 65536
SF 500.1300013 MHz
WDW EM
SSB 0
LB 0.30 Hz
GB 0
PC 1.00

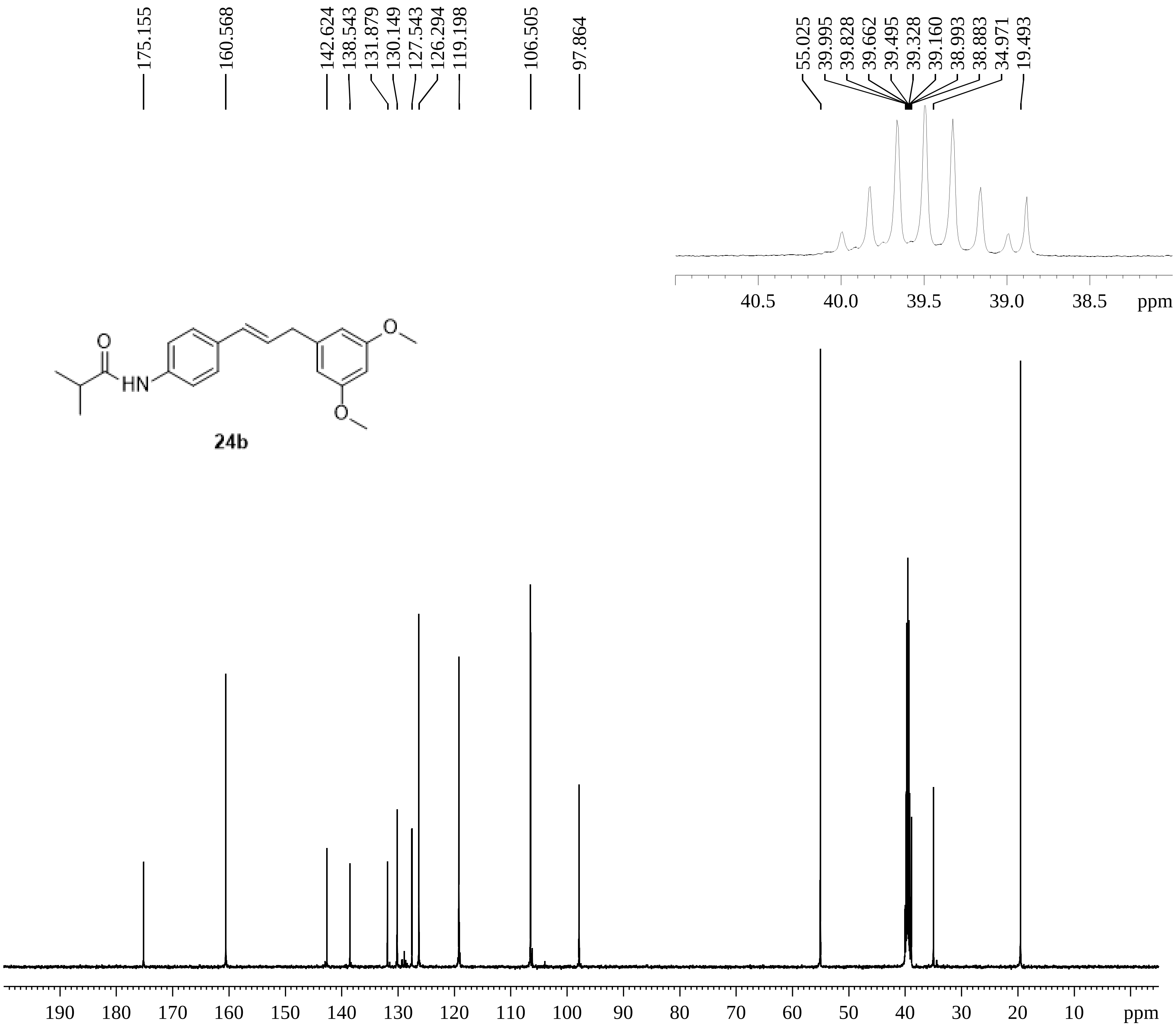

24b

Current Data Parameters
NAME NMR16070013-LSS-40
EXPNO 2
PROCNO 1

F2 - Acquisition Parameters
Date_ 20160706
Time 10.11
INSTRUM spect
PROBHD 5 mm PABBO BB-
PULPROG zgpg30
TD 65536
SOLVENT DMSO
NS 256
DS 4
SWH 34722.223 Hz
FIDRES 0.529819 Hz
AQ 0.9437184 sec
RG 198.55
DW 14.400 usec
DE 6.50 usec
TE 298.2 K
D1 2.00000000 sec
D11 0.03000000 sec
TD0 1

======== CHANNEL f1 =======
SFO1 125.7703637 MHz
NUC1 13C
P1 9.12 usec
PLW1 125.88999939 W

======== CHANNEL f2 =======
SFO2 500.1320005 MHz
NUC2 1H
CPDPRG[2 waltz16
PCPD2 80.00 usec
PLW2 19.95299911 W
PLW12 0.41303000 W
PLW13 0.26434001 W

F2 - Processing parameters
SI 32768
SF 125.7578453 MHz
WDW EM
SSB 0
LB 1.00 Hz
GB 0
PC 1.40

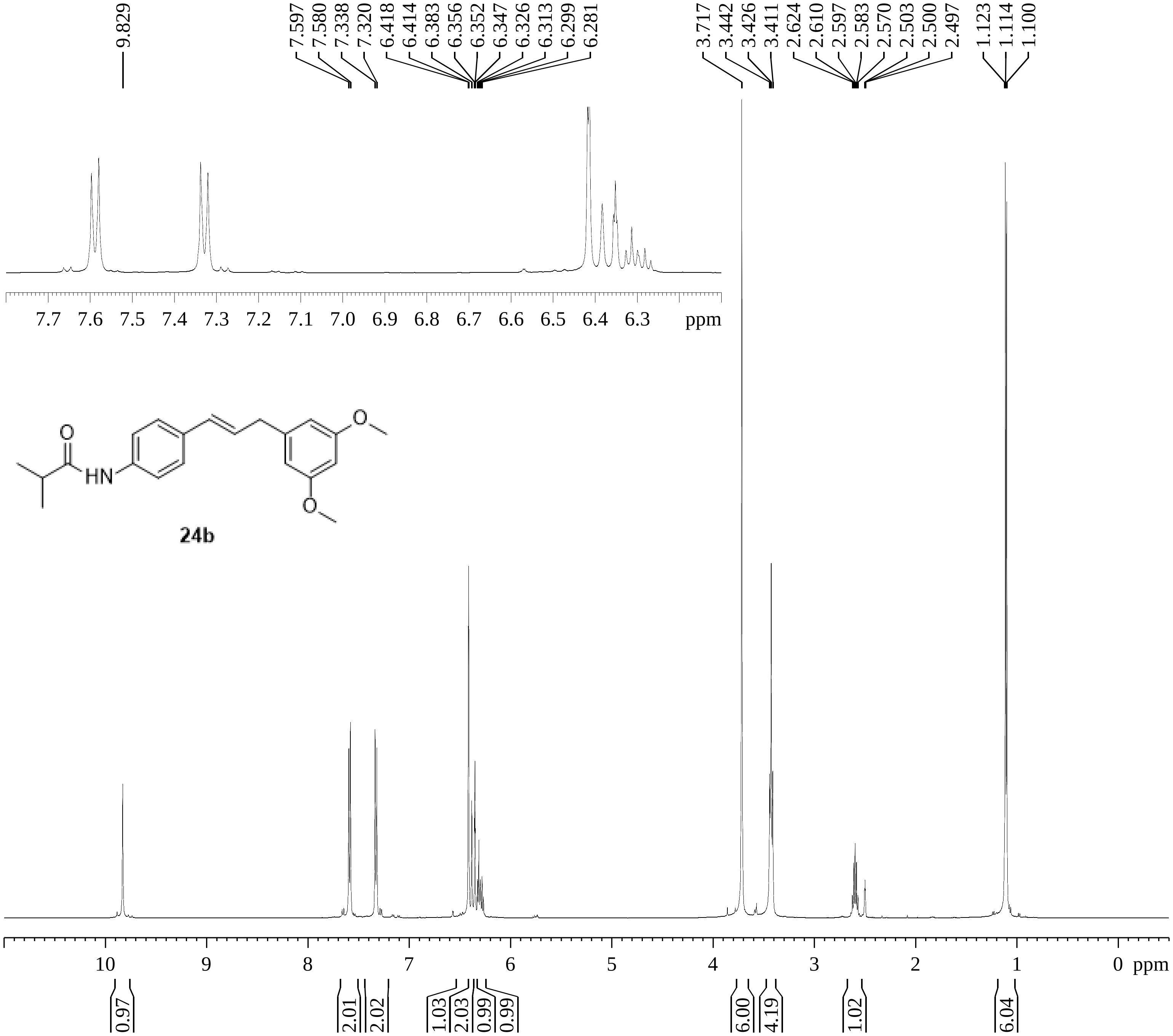

Current Data Parameters
NAME NMR16070013-LSS-40
EXPNO 1
PROCNO 1

F2 - Acquisition Parameters
Date_ 20160705
Time 11.16
INSTRUM spect
PROBHD 5 mm PABBO BB-
PULPROG zg30
TD 65536
SOLVENT DMSO
NS 32
DS 2
SWH 10000.000 Hz
FIDRES 0.152588 Hz
AQ 3.2767999 sec
RG 32.04
DW 50.000 usec
DE 6.50 usec
TE 299.1 K
D1 1.00000000 sec
TD0 1

======== CHANNEL f1 =======
SFO1 500.1325007 MHz
NUC1 1H
P1 11.51 usec
PLW1 19.95299911 W

F2 - Processing parameters
SI 65536
SF 500.1300044 MHz
WDW EM
SSB 0
LB 0.30 Hz
GB 0
PC 1.00

MCX

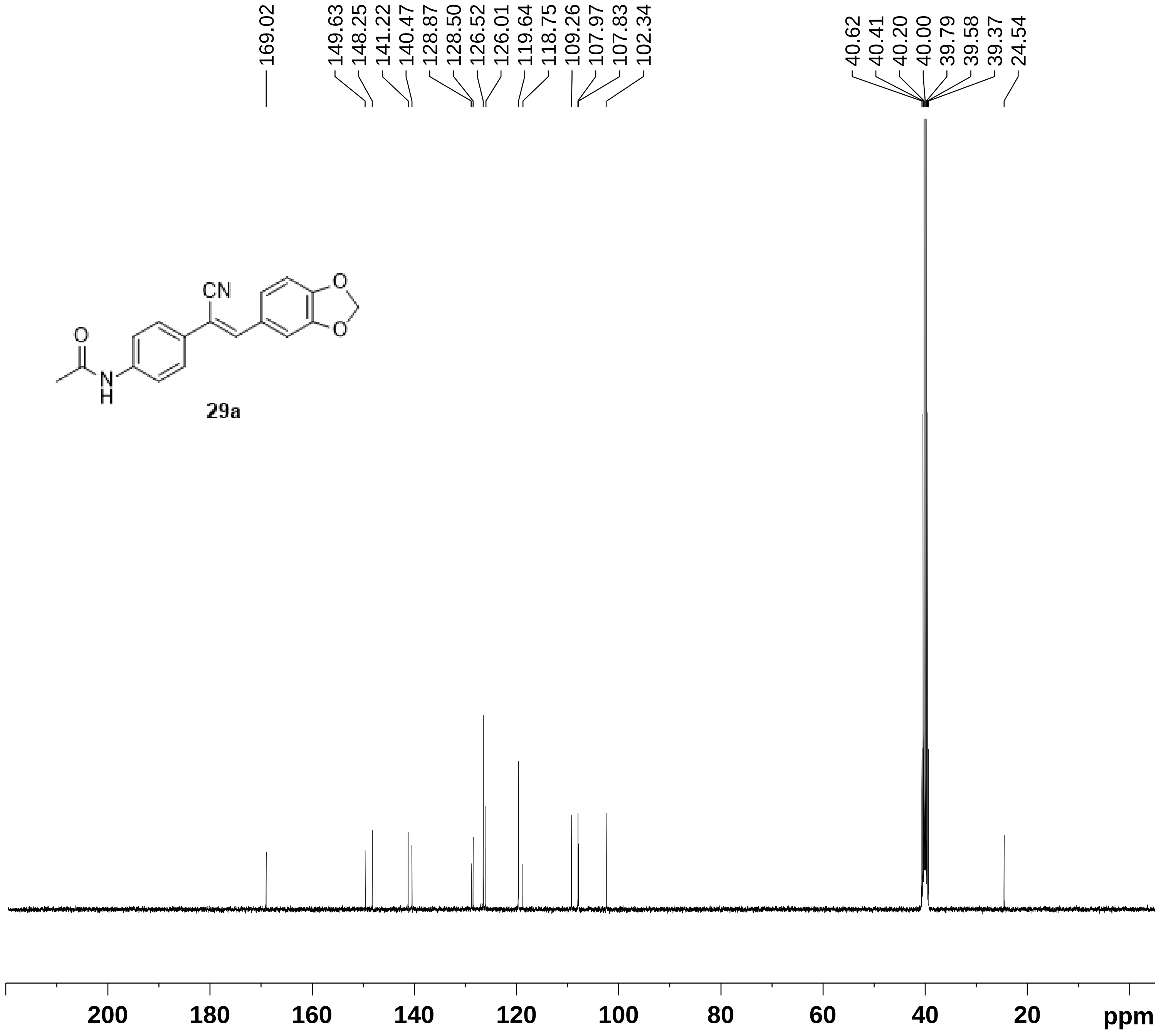

29a

Current Data Parameters
NAME MCX-70
EXPNO 2
PROCNO 1

F2 - Acquisition Parameters
Date_ 20190225
Time 15.00 h
INSTRUM spect
PROBHD Z116098_0436 (
PULPROG zgpg30
TD 65536
SOLVENT DMSO
NS 1024
DS 4
SWH 24038.461 Hz
FIDRES 0.733596 Hz
AQ 1.3631488 sec
RG 202.1
DW 20.800 usec
DE 6.50 usec
TE 298.0 K
D1 2.00000000 sec
D11 0.03000000 sec
TD0 1
SFO1 100.6278593 MHz
NUC1 13C
P1 10.00 usec
PLW1 68.03199768 W
SFO2 400.1516006 MHz
NUC2 1H
CPDPRG[2 waltz16
PCPD2 80.00 usec
PLW2 15.00300026 W
PLW12 0.20275000 W
PLW13 0.10182000 W

F2 - Processing parameters
SI 32768
SF 100.6177975 MHz
WDW EM
SSB 0
LB 1.00 Hz
GB 0
PC 1.40

MCX

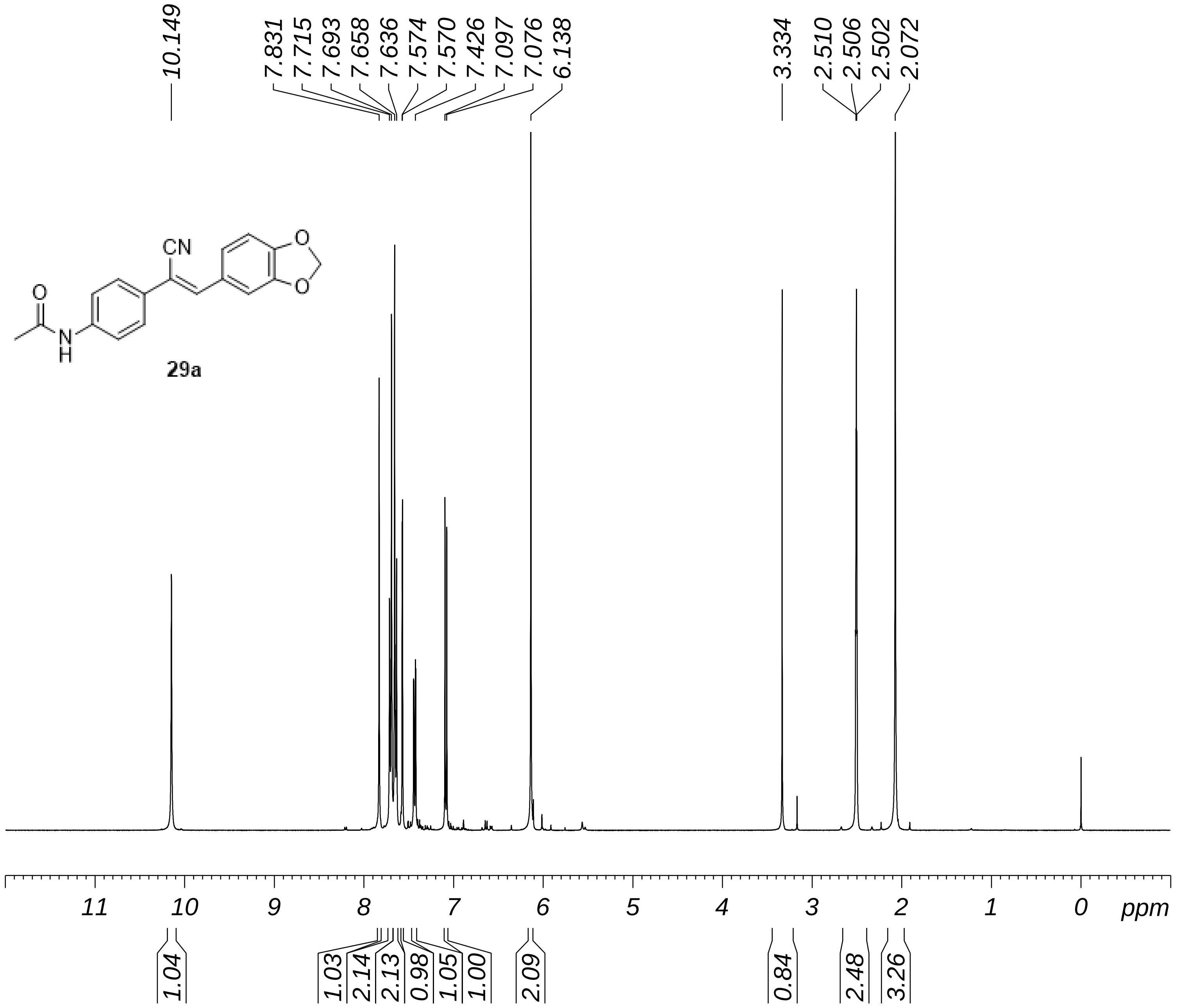

Current Data Parameters
NAME MCX-70
EXPNO 1
PROCNO 1

F2 - Acquisition Parameters
Date_ 20190221
Time 18.44 h
INSTRUM spect
PROBHD Z116098_0436 (
PULPROG zg30
TD 65536
SOLVENT DMSO
NS 16
DS 2
SWH 8012.820 Hz
FIDRES 0.244532 Hz
AQ 4.0894465 sec
RG 111.98
DW 62.400 usec
DE 6.50 usec
TE 298.0 K
D1 1.00000000 sec
TD0 1
SFO1 400.1524709 MHz
NUC1 1H
P1 8.00 usec
PLW1 15.00300026 W

F2 - Processing parameters
SI 65536
SF 400.1500008 MHz
WDW EM
SSB 0
LB 0.30 Hz
GB 0
PC 1.00

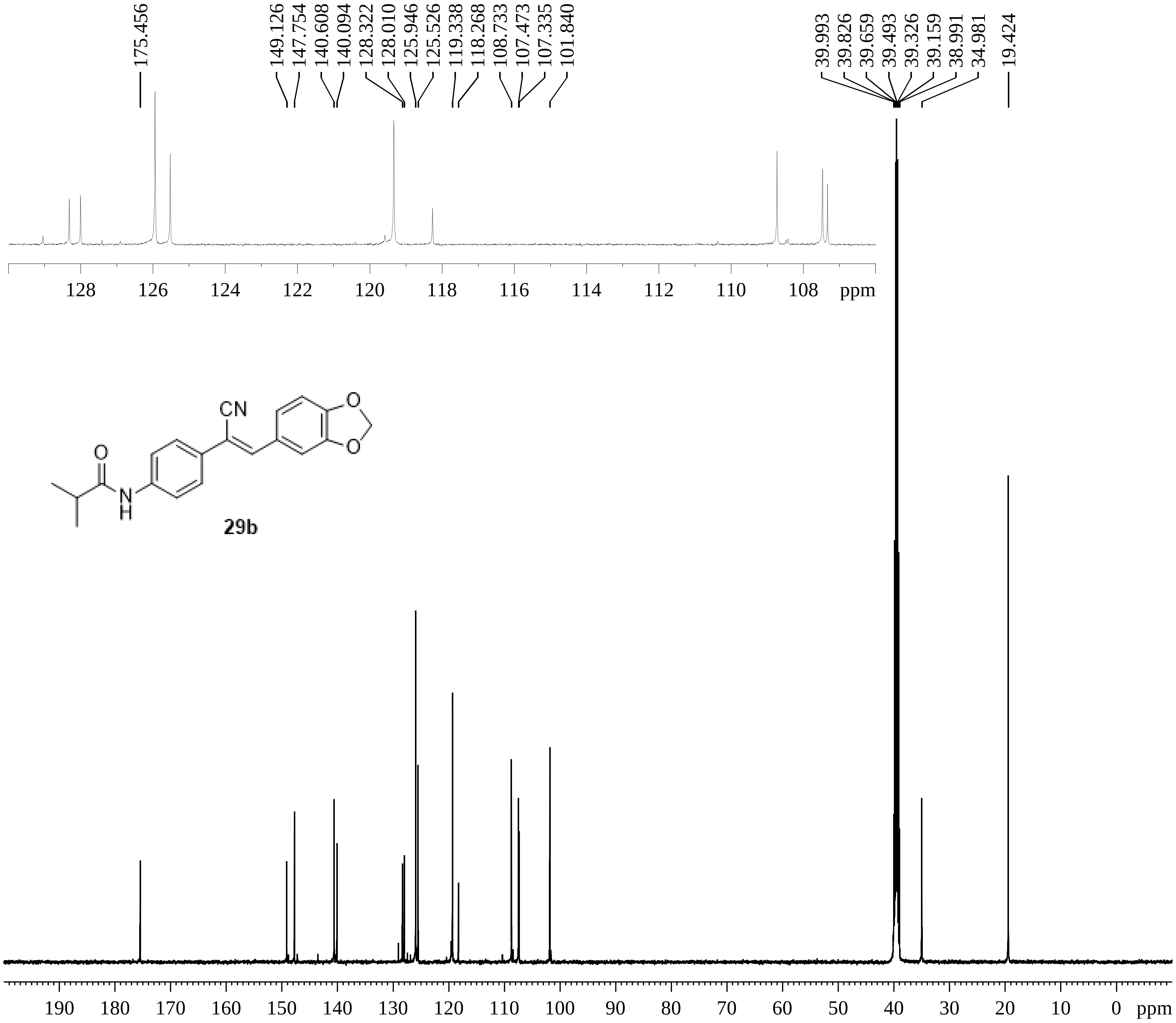

Current Data Parameters
NAME NMR16090172-LSS-35-C
EXPNO 1
PROCNO 1

F2 - Acquisition Parameters
Date_ 20160930
Time 12.59
INSTRUM spect
PROBHD 5 mm PABBO BB-
PULPROG zgpg30
TD 65536
SOLVENT DMSO
NS 1024
DS 4
SWH 34722.223 Hz
FIDRES 0.529819 Hz
AQ 0.9437184 sec
RG 198.55
DW 14.400 usec
DE 6.50 usec
TE 298.2 K
D1 2.00000000 sec
D11 0.03000000 sec
TD0 1

======== CHANNEL f1 =======
SFO1 125.7703637 MHz
NUC1 13C
P1 9.12 usec
PLW1 125.88999939 W

======== CHANNEL f2 =======
SFO2 500.1320005 MHz
NUC2 1H
CPDPRG[2 waltz16
PCPD2 80.00 usec
PLW2 19.95299911 W
PLW12 0.41303000 W
PLW13 0.26434001 W

F2 - Processing parameters
SI 32768
SF 125.7578495 MHz
WDW EM
SSB 0
LB 1.00 Hz
GB 0
PC 1.40

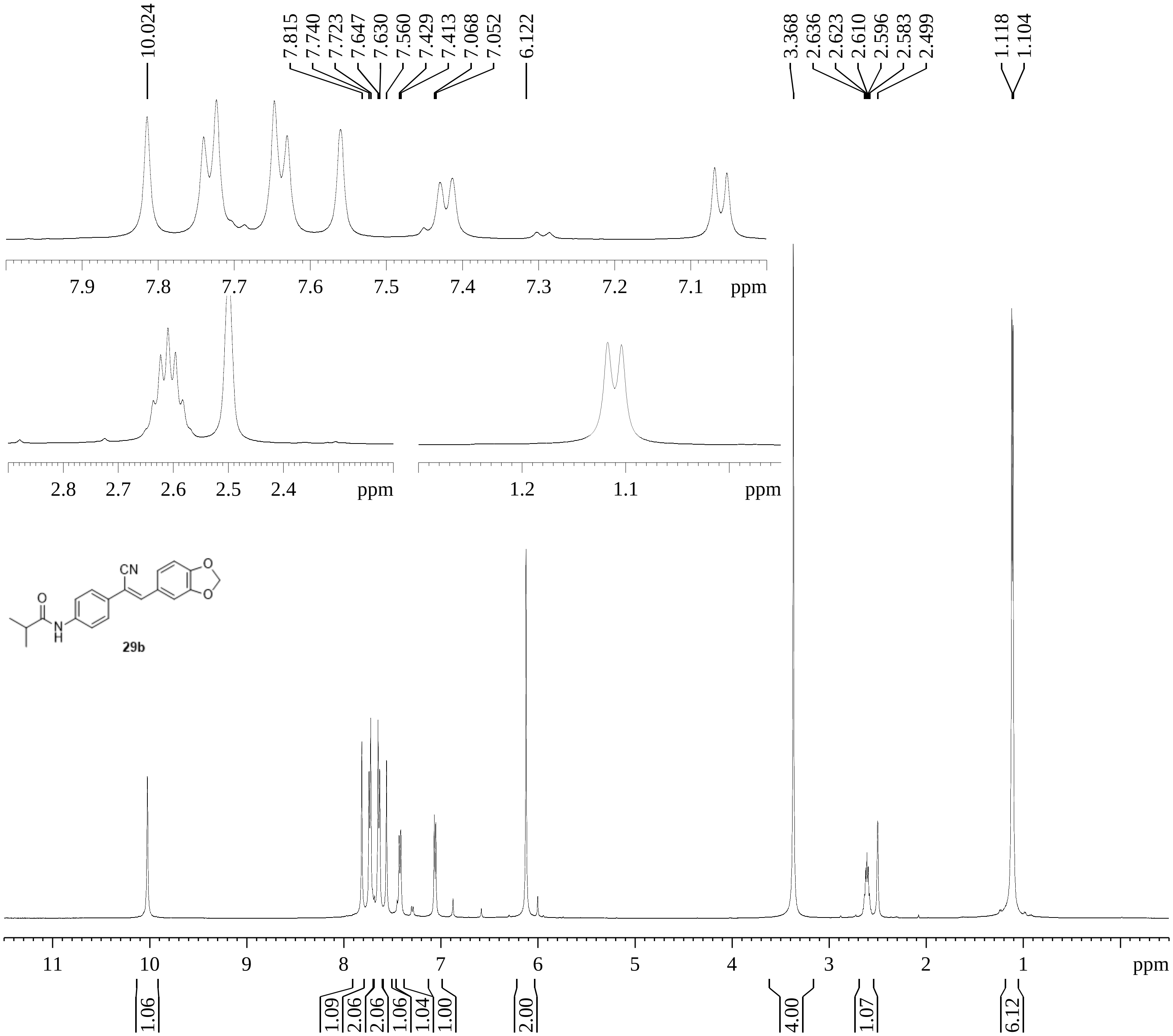

Current Data Parameters
NAME NMR16090160-LSS-35
EXPNO 1
PROCNO 1

F2 - Acquisition Parameters
Date_ 20160928
Time 13.23
INSTRUM spect
PROBHD 5 mm PABBO BB-
PULPROG zg30
TD 65536
SOLVENT DMSO
NS 128
DS 2
SWH 10000.000 Hz
FIDRES 0.152588 Hz
AQ 3.2767999 sec
RG 62.41
DW 50.000 usec
DE 6.50 usec
TE 298.2 K
D1 1.00000000 sec
TD0 1

======== CHANNEL f1 =======
SFO1 500.1330885 MHz
NUC1 1H
P1 11.51 usec
PLW1 19.95299911 W

F2 - Processing parameters
SI 65536
SF 500.1300050 MHz
WDW EM
SSB 0
LB 0.30 Hz
GB 0
PC 1.00

MCX

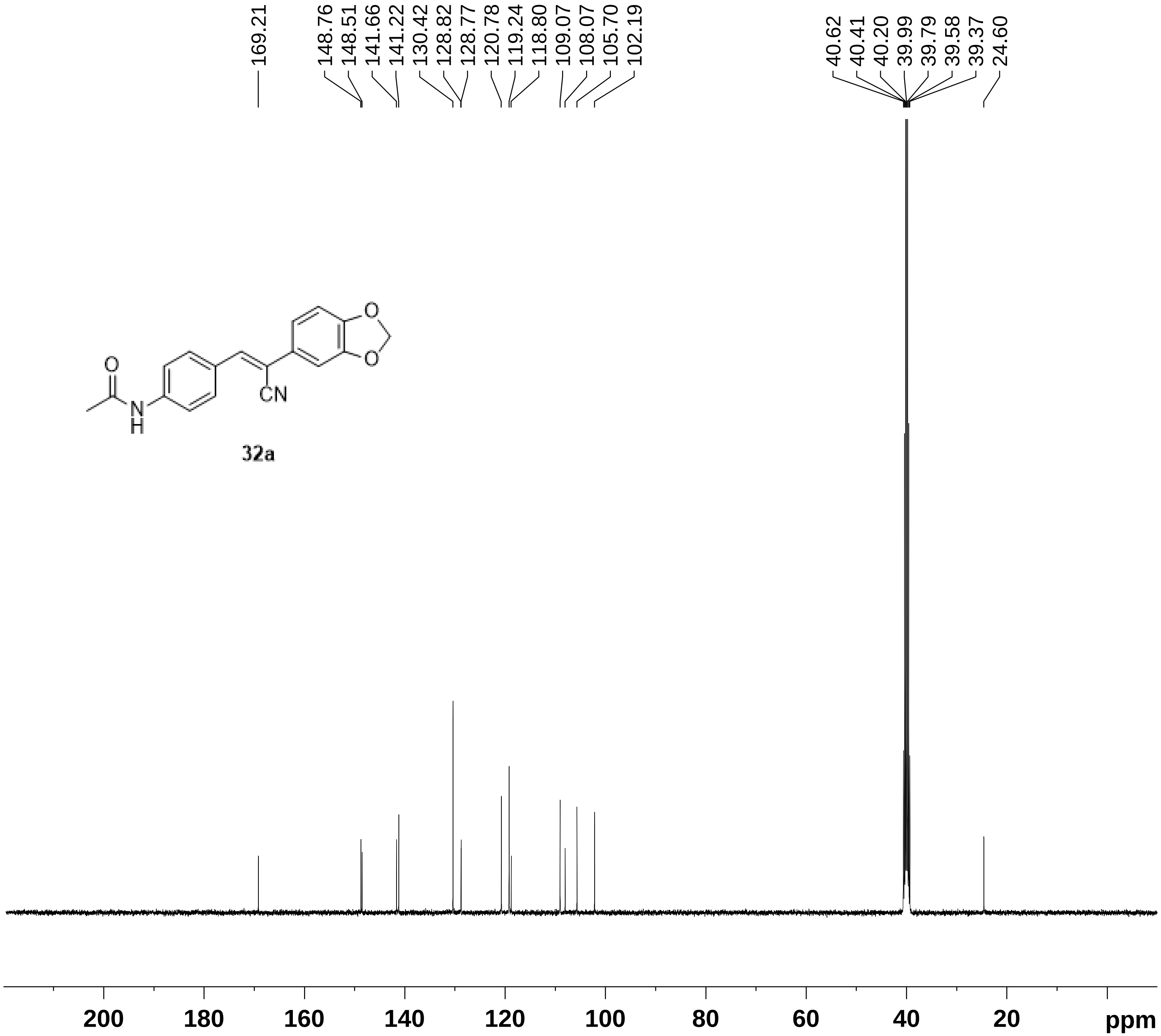

32a

Current Data Parameters
NAME MCX-71
EXPNO 2
PROCNO 1

F2 - Acquisition Parameters
Date_ 20190225
Time 13.51 h
INSTRUM spect
PROBHD Z116098_0436 (
PULPROG zgpg30
TD 65536
SOLVENT DMSO
NS 1024
DS 4
SWH 24038.461 Hz
FIDRES 0.733596 Hz
AQ 1.3631488 sec
RG 202.1
DW 20.800 usec
DE 6.50 usec
TE 298.0 K
D1 2.00000000 sec
D11 0.03000000 sec
TD0 1
SFO1 100.6278593 MHz
NUC1 13C
P1 10.00 usec
PLW1 68.03199768 W
SFO2 400.1516006 MHz
NUC2 1H
CPDPRG[2 waltz16
PCPD2 80.00 usec
PLW2 15.00300026 W
PLW12 0.20275000 W
PLW13 0.10182000 W

F2 - Processing parameters
SI 32768
SF 100.6177975 MHz
WDW EM
SSB 0
LB 1.00 Hz
GB 0
PC 1.40

MCX

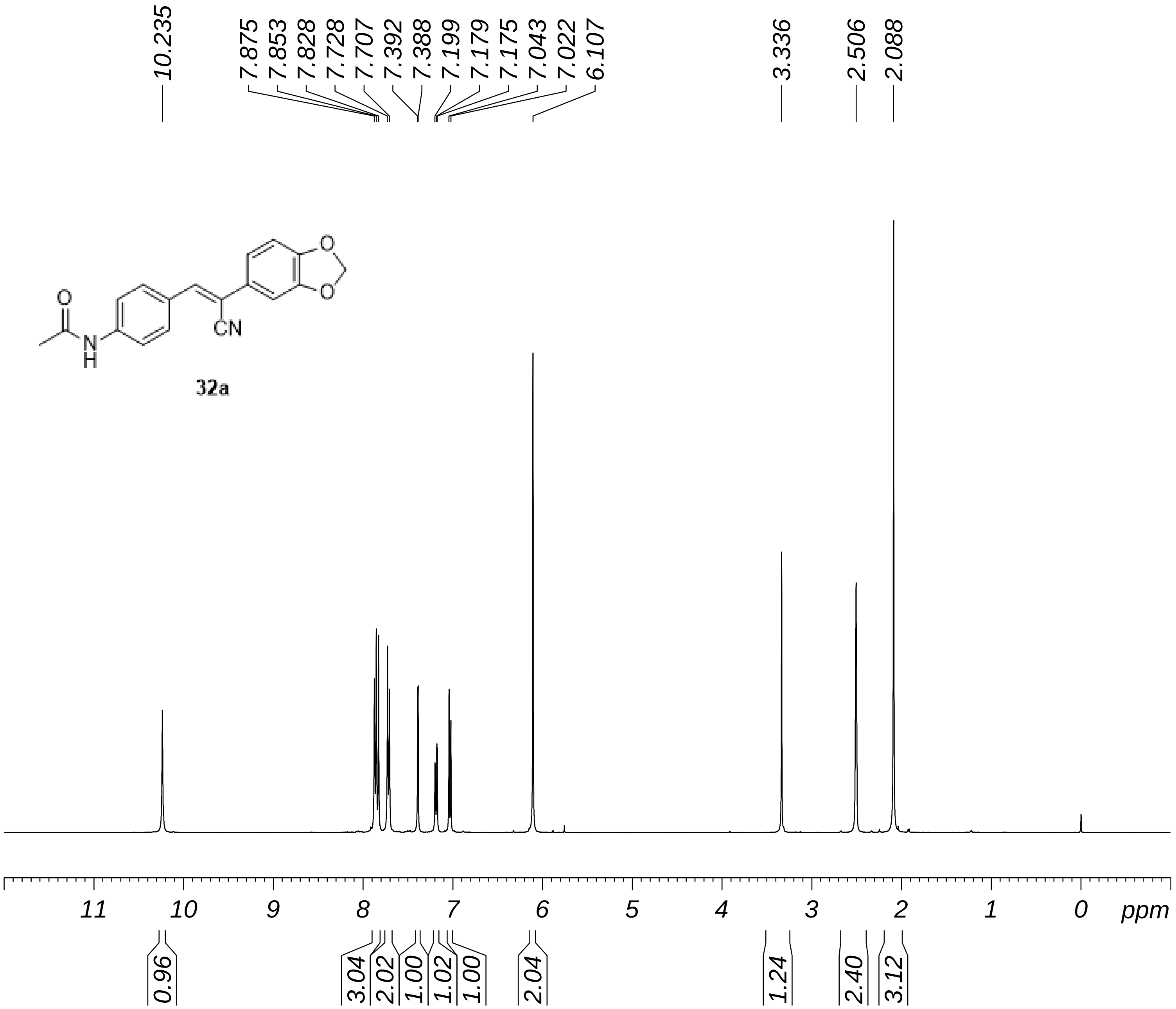

Current Data Parameters
NAME MCX-71
EXPNO 1
PROCNO 1

F2 - Acquisition Parameters
Date_ 20190221
Time 18.33 h
INSTRUM spect
PROBHD Z116098_0436 (
PULPROG zg30
TD 65536
SOLVENT DMSO
NS 16
DS 2
SWH 8012.820 Hz
FIDRES 0.244532 Hz
AQ 4.0894465 sec
RG 111.98
DW 62.400 usec
DE 6.50 usec
TE 298.0 K
D1 1.00000000 sec
TD0 1
SFO1 400.1524709 MHz
NUC1 1H
P1 8.00 usec
PLW1 15.00300026 W

F2 - Processing parameters
SI 65536
SF 400.1500006 MHz
WDW EM
SSB 0
LB 0.30 Hz
GB 0
PC 1.00

MCX

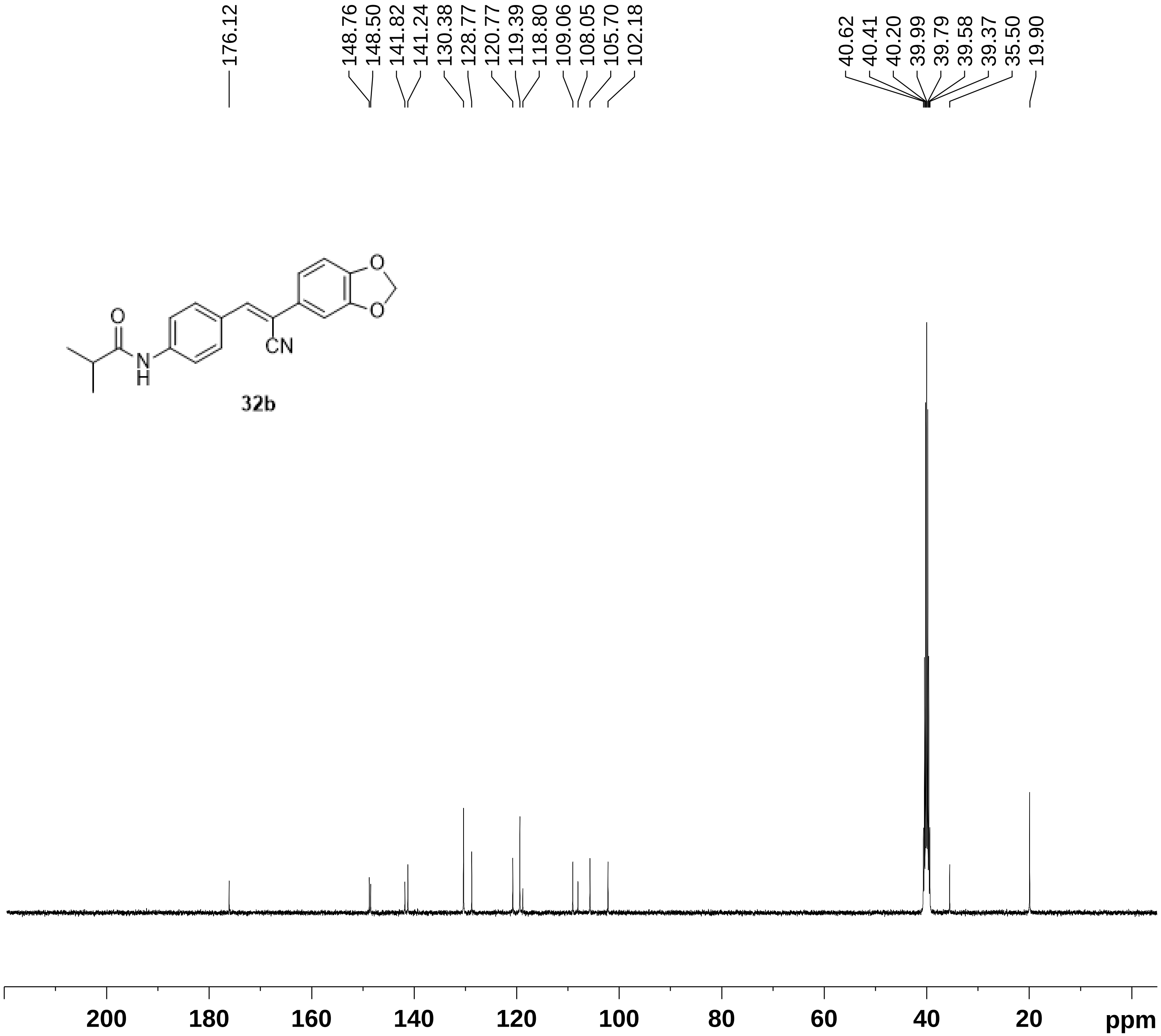

Current Data Parameters
NAME MCX-67-C
EXPNO 1
PROCNO 1

F2 - Acquisition Parameters
Date_ 20190111
Time 16.35 h
INSTRUM spect
PROBHD Z116098_0436 (
PULPROG zgpg30
TD 65536
SOLVENT DMSO
NS 835
DS 4
SWH 24038.461 Hz
FIDRES 0.733596 Hz
AQ 1.3631488 sec
RG 202.1
DW 20.800 usec
DE 6.50 usec
TE 298.0 K
D1 2.00000000 sec
D11 0.03000000 sec
TD0 1
SFO1 100.6278593 MHz
NUC1 13C
P1 10.00 usec
PLW1 68.03199768 W
SFO2 400.1516006 MHz
NUC2 1H
CPDPRG[2 waltz16
PCPD2 80.00 usec
PLW2 15.00300026 W
PLW12 0.15003000 W
PLW13 0.07534400 W

F2 - Processing parameters
SI 32768
SF 100.6177975 MHz
WDW EM
SSB 0
LB 1.00 Hz
GB 0
PC 1.40

MCX

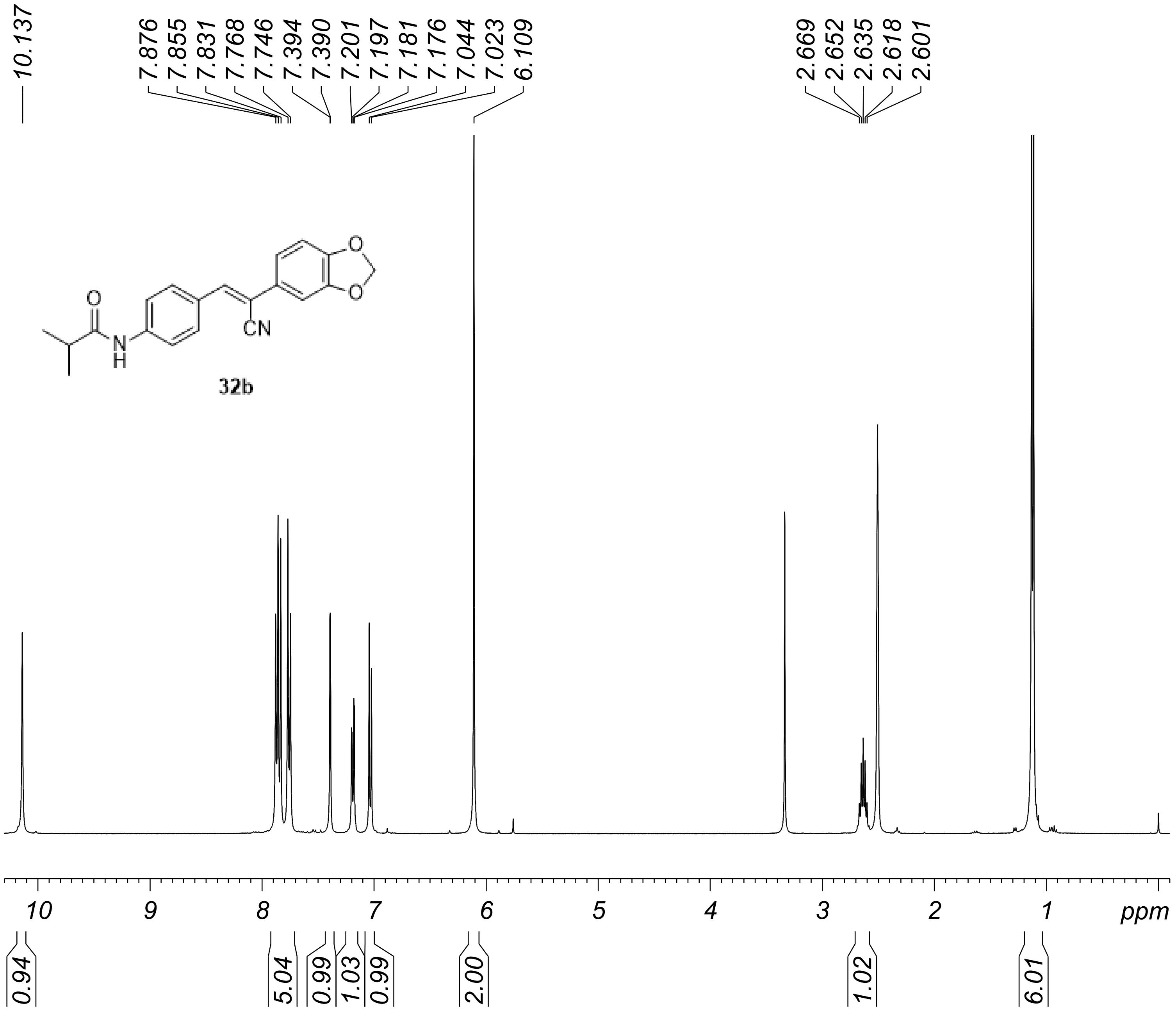

Current Data Parameters
NAME MCX-67-H
EXPNO 2
PROCNO 1

F2 - Acquisition Parameters
Date_ 20190221
Time 18.48 h
INSTRUM spect
PROBHD Z116098_0436 (
PULPROG zg30
TD 65536
SOLVENT DMSO
NS 16
DS 2
SWH 8012.820 Hz
FIDRES 0.244532 Hz
AQ 4.0894465 sec
RG 98.25
DW 62.400 usec
DE 6.50 usec
TE 298.0 K
D1 1.00000000 sec
TD0 1
SFO1 400.1524709 MHz
NUC1 1H
P1 8.00 usec
PLW1 15.00300026 W

F2 - Processing parameters
SI 65536
SF 400.1500005 MHz
WDW EM
SSB 0
LB 0.30 Hz
GB 0
PC 1.00

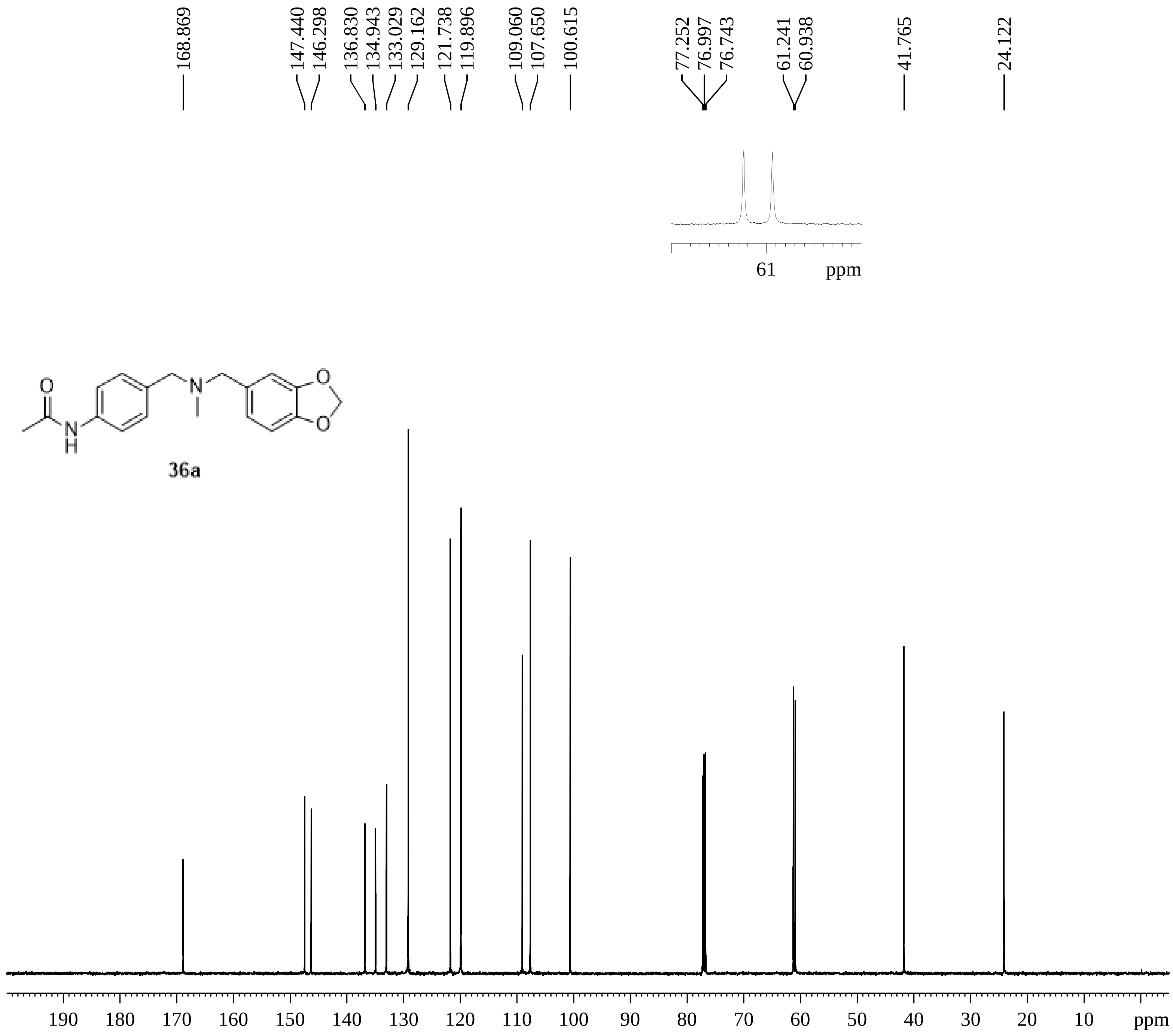

36a

Current Data Parameters
NAME 150033-LSS-3
EXPNO 2
PROCNO 1

F2 - Acquisition Parameters
Date_ 20150612
Time 7.47
INSTRUM spect
PROBHD 5 mm PABBO BB-
PULPROG zgpg30
TD 65536
SOLVENT CDCl3
NS 256
DS 4
SWH 34722.223 Hz
FIDRES 0.529819 Hz
AQ 0.9437184 sec
RG 198.55
DW 14.400 usec
DE 6.50 usec
TE 303.2 K
D1 2.00000000 sec
D11 0.03000000 sec
TD0 1

======== CHANNEL f1 =======
SFO1 125.7703637 MHz
NUC1 13C
P1 8.99 usec
PLW1 125.88999939 W

======== CHANNEL f2 =======
SFO2 500.1320005 MHz
NUC2 1H
CPDPRG[2 waltz16
PCPD2 80.00 usec
PLW2 19.95299911 W
PLW12 0.39528000 W
PLW13 0.25297999 W

F2 - Processing parameters
SI 32768
SF 125.7578151 MHz
WDW EM
SSB 0
LB 1.00 Hz
GB 0
PC 1.40

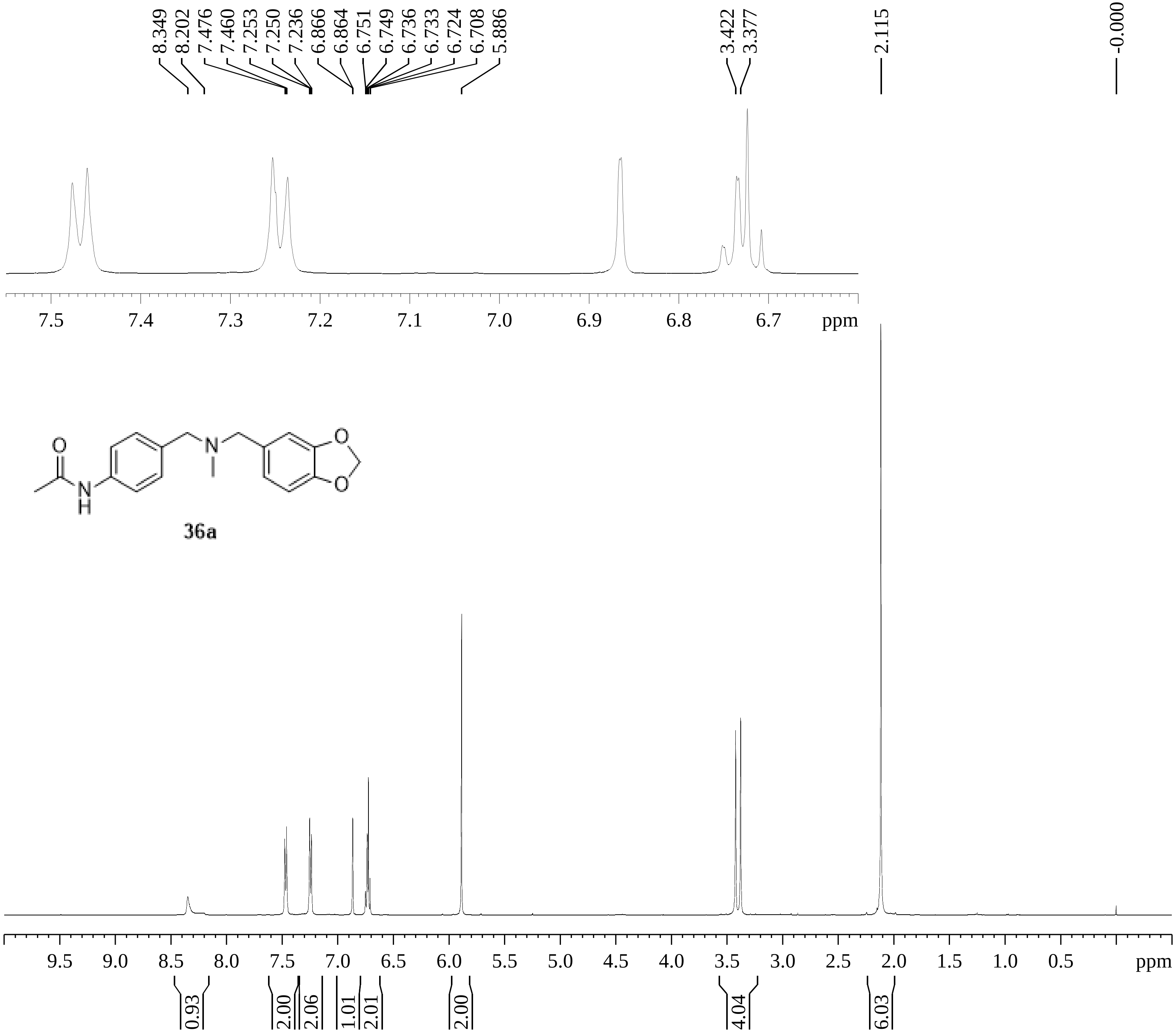

36a

Current Data Parameters
NAME 150033-LSS-3
EXPNO 1
PROCNO 1

F2 - Acquisition Parameters
Date_ 20150610
Time 11.13
INSTRUM spect
PROBHD 5 mm PABBO BB-
PULPROG zg30
TD 65536
SOLVENT CDCl3
NS 16
DS 0
SWH 10000.000 Hz
FIDRES 0.152588 Hz
AQ 3.2767999 sec
RG 20.1
DW 50.000 usec
DE 6.50 usec
TE 303.1 K
D1 1.00000000 sec
TD0 1

======== CHANNEL f1 =======
SFO1 500.1330885 MHz
NUC1 1H
P1 11.26 usec
PLW1 19.95299911 W

F2 - Processing parameters
SI 65536
SF 500.1300170 MHz
WDW EM
SSB 0
LB 0.30 Hz
GB 0
PC 1.00

FBZ

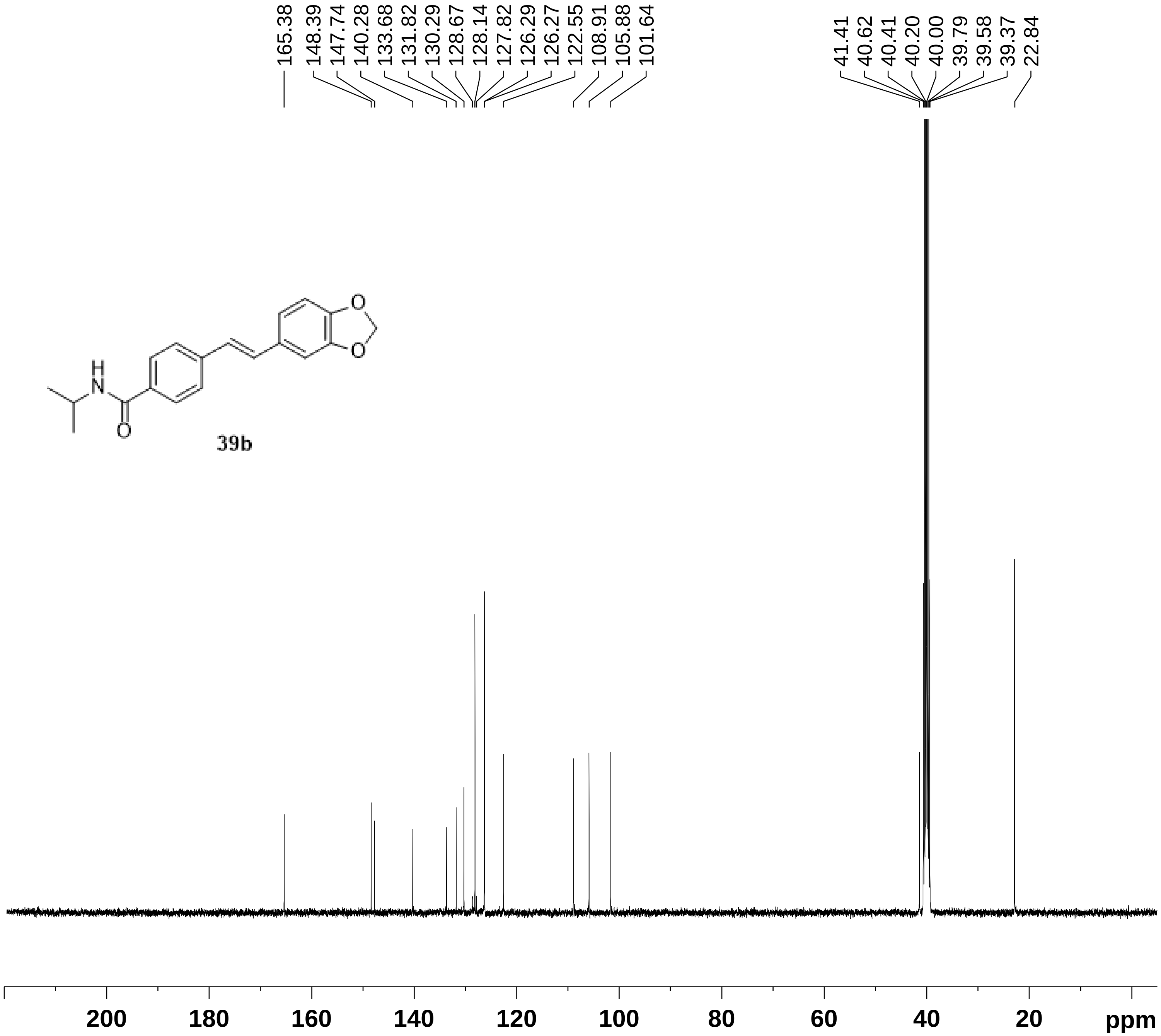

39b

Current Data Parameters
NAME MCX-1-1201-C
EXPNO 1
PROCNO 1

F2 - Acquisition Parameters
Date_ 20171201
Time 23.17 h
INSTRUM spect
PROBHD Z116098_0436 (
PULPROG zgpg30
TD 65536
SOLVENT DMSO
NS 2200
DS 4
SWH 24038.461 Hz
FIDRES 0.733596 Hz
AQ 1.3631488 sec
RG 202.1
DW 20.800 usec
DE 6.50 usec
TE 298.0 K
D1 2.00000000 sec
D11 0.03000000 sec
TD0 1
SFO1 100.6278593 MHz
NUC1 13C
P1 10.00 usec
PLW1 68.03199768 W
SFO2 400.1516006 MHz
NUC2 1H
CPDPRG[2 waltz16
PCPD2 80.00 usec
PLW2 15.00300026 W
PLW12 0.22284999 W
PLW13 0.11191000 W

F2 - Processing parameters
SI 32768
SF 100.6177975 MHz
WDW EM
SSB 0
LB 1.00 Hz
GB 0
PC 1.40

FBZ

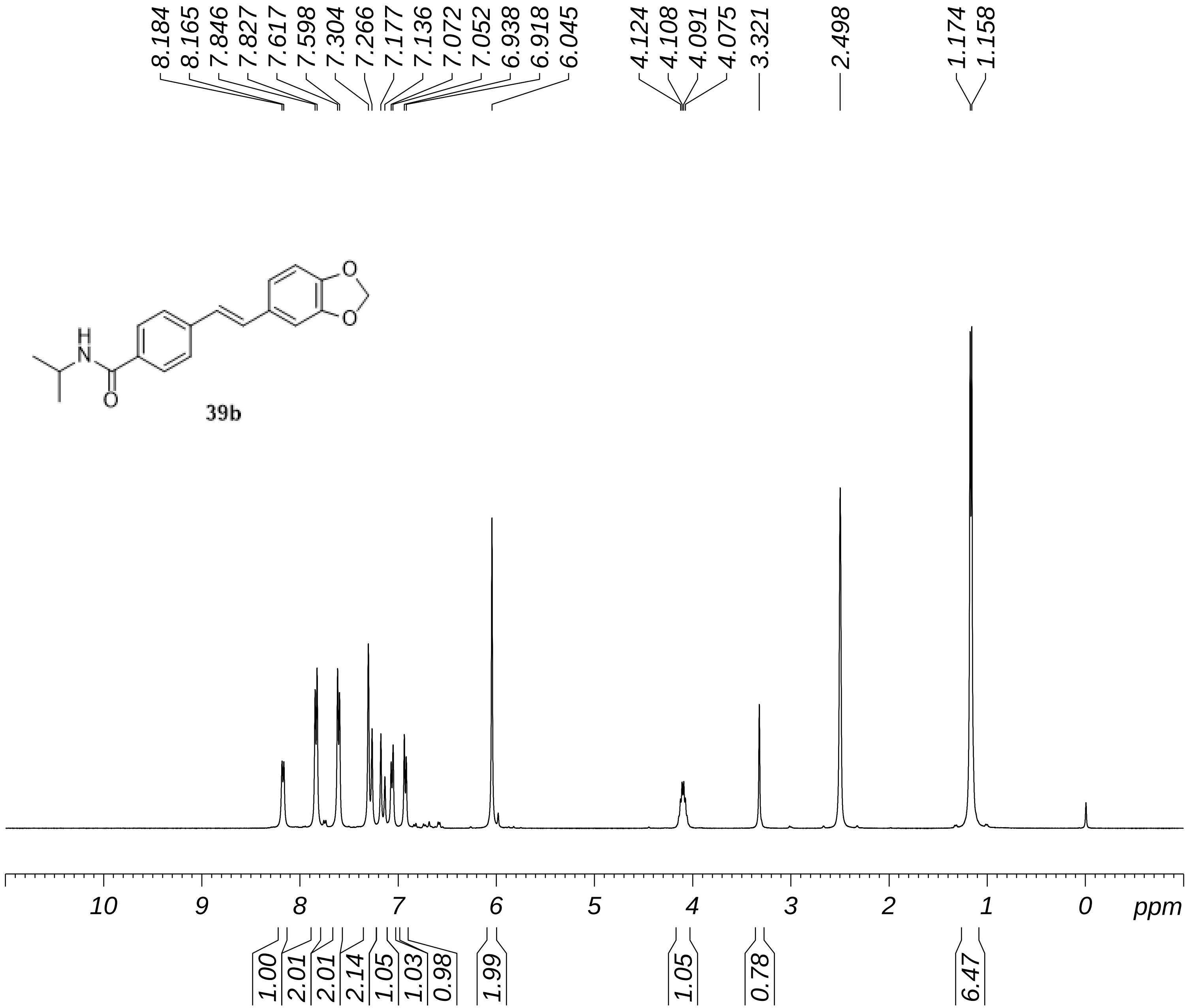

Current Data Parameters
NAME MCX-1-1201
EXPNO 1
PROCNO 1

F2 - Acquisition Parameters
Date_ 20171201
Time 11.12 h
INSTRUM spect
PROBHD Z116098_0436 (
PULPROG zg30
TD 65536
SOLVENT DMSO
NS 16
DS 2
SWH 8012.820 Hz
FIDRES 0.244532 Hz
AQ 4.0894465 sec
RG 98.25
DW 62.400 usec
DE 6.50 usec
TE 298.0 K
D1 1.00000000 sec
TD0 1
SFO1 400.1524709 MHz
NUC1 1H
P1 9.75 usec
PLW1 15.00300026 W

F2 - Processing parameters
SI 65536
SF 400.1500033 MHz
WDW EM
SSB 0
LB 0.30 Hz
GB 0
PC 1.00

MCX-2

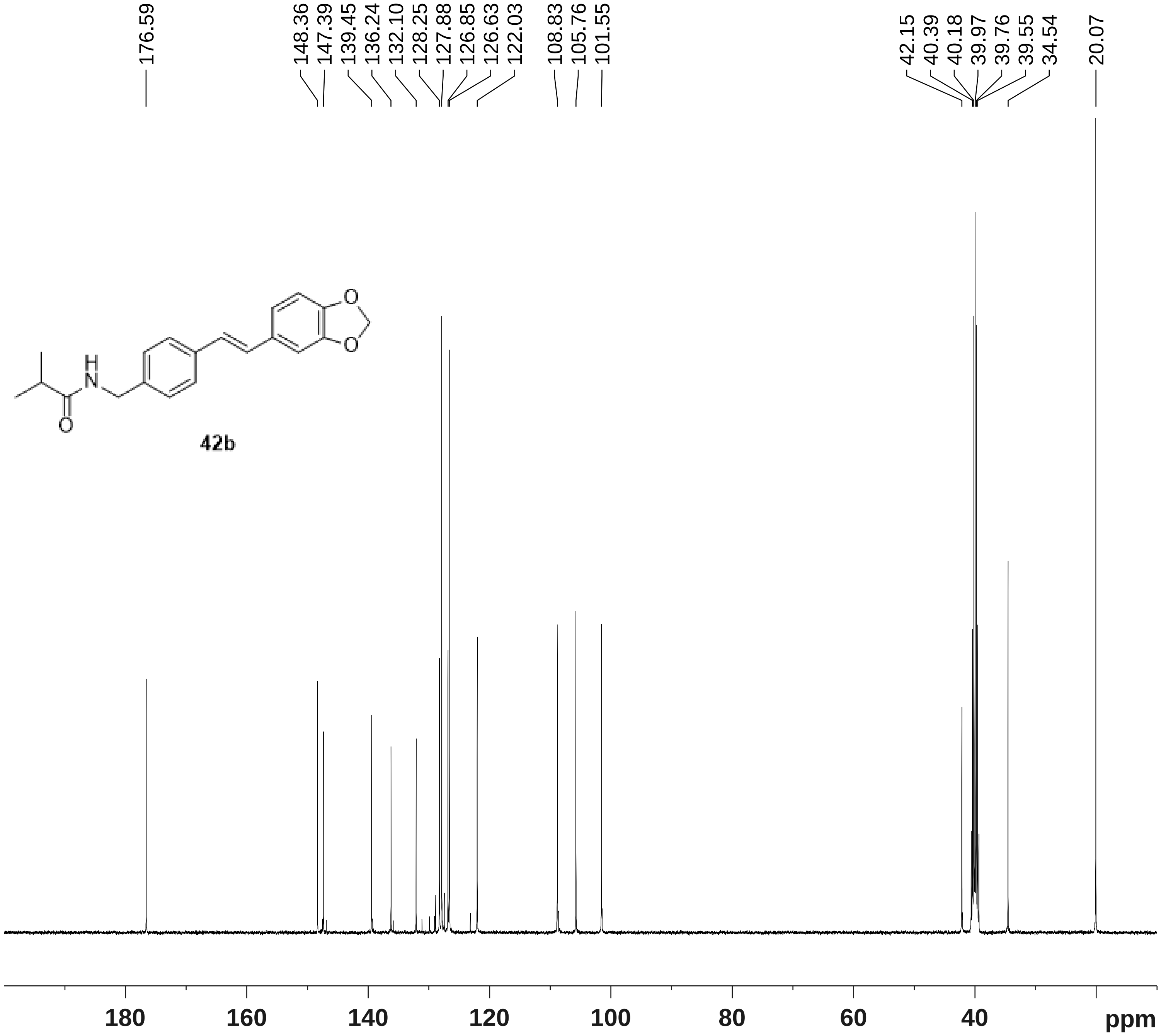

Current Data Parameters
NAME MCX-2-C
EXPNO 1
PROCNO 1

F2 - Acquisition Parameters
Date_ 20161215
Time 11.08 h
INSTRUM spect
PROBHD Z116098_0436 (
PULPROG zgpg30
TD 65536
SOLVENT DMSO
NS 1024
DS 4
SWH 24038.461 Hz
FIDRES 0.733596 Hz
AQ 1.3631488 sec
RG 202.1
DW 20.800 usec
DE 6.50 usec
TE 298.0 K
D1 2.00000000 sec
D11 0.03000000 sec
TD0 1
SFO1 100.6278593 MHz
NUC1 13C
P1 10.00 usec
PLW1 68.03199768 W
SFO2 400.1516006 MHz
NUC2 1H
CPDPRG[2 waltz16
PCPD2 80.00 usec
PLW2 15.00300026 W
PLW12 0.22284999 W
PLW13 0.11191000 W

F2 - Processing parameters
SI 32768
SF 100.6177975 MHz
WDW EM
SSB 0
LB 1.00 Hz
GB 0
PC 1.40

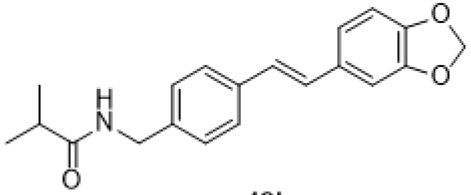

42b

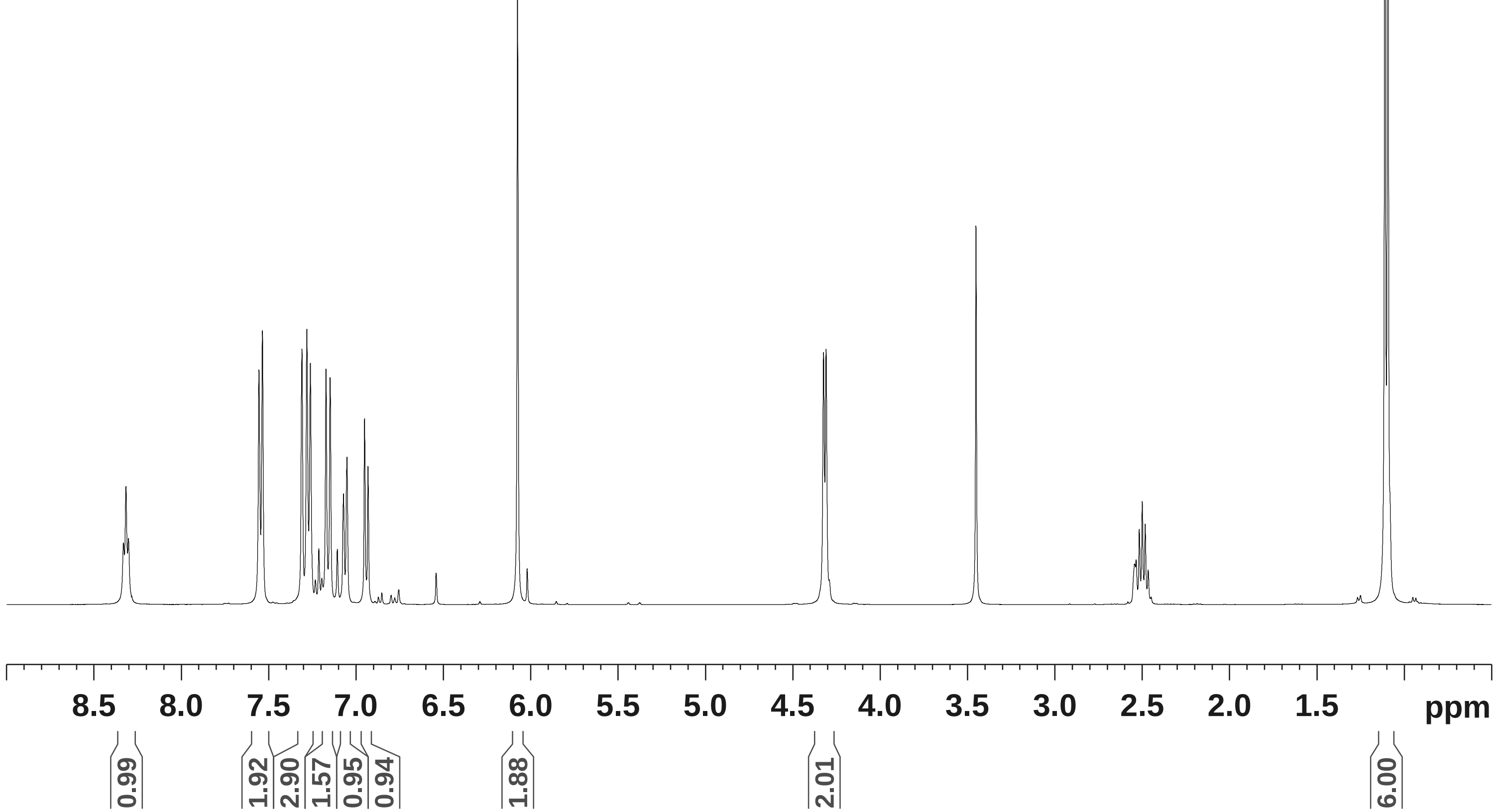

Current Data Parameters
NAME MCX-2
EXPNO 1
PROCNO 1

F2 - Acquisition Parameters
Date_ 20161214
Time 13.13 h
INSTRUM spect
PROBHD Z116098_0436 (
PULPROG zg30
TD 65536
SOLVENT DMSO
NS 16
DS 2
SWH 8012.820 Hz
FIDRES 0.244532 Hz
AQ 4.0894465 sec
RG 13.82
DW 62.400 usec
DE 6.50 usec
TE 298.0 K
D1 1.00000000 sec
TD0 1
SFO1 400.1524709 MHz
NUC1 1H
P1 9.75 usec
PLW1 15.00300026 W

F2 - Processing parameters
SI 65536
SF 400.1499852 MHz
WDW EM
SSB 0
LB 0.30 Hz
GB 0
PC 1.00

HRMS for the representative compounds.

8c

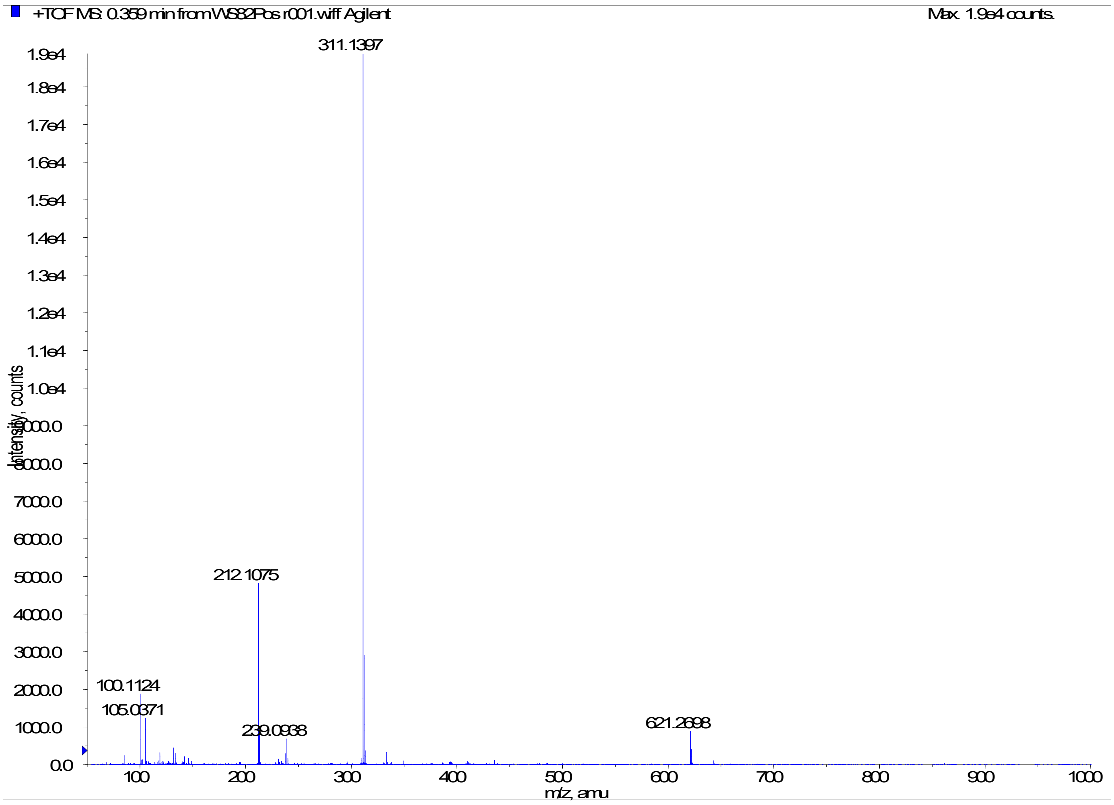

8d

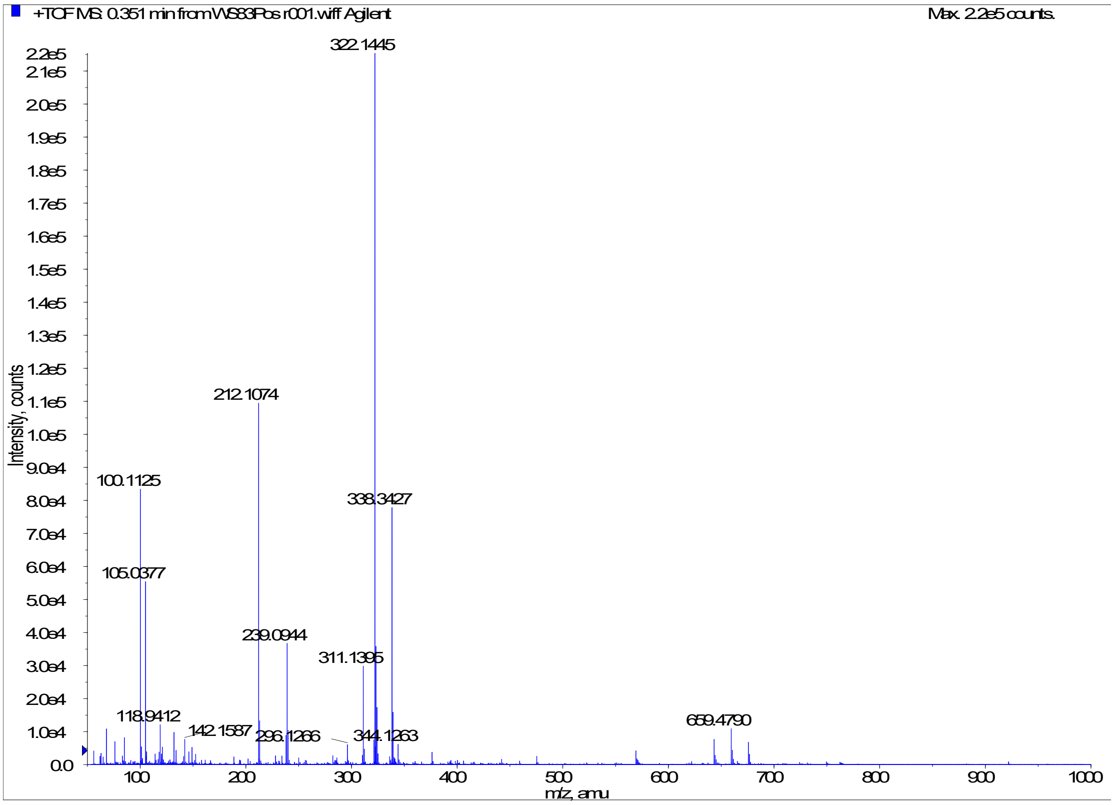

8e

Peking University Mass Spectrometry Sample Analysis Report

Analysis Info

Analysis Name	FTMS-17010083_Pos_20170117_000004.d	Acquisition Date	1/17/2017 3:18:35 PM
Sample	LSS-50	Instrument	Bruker Solarix XR FTMS
Comment		Operator	Peking University

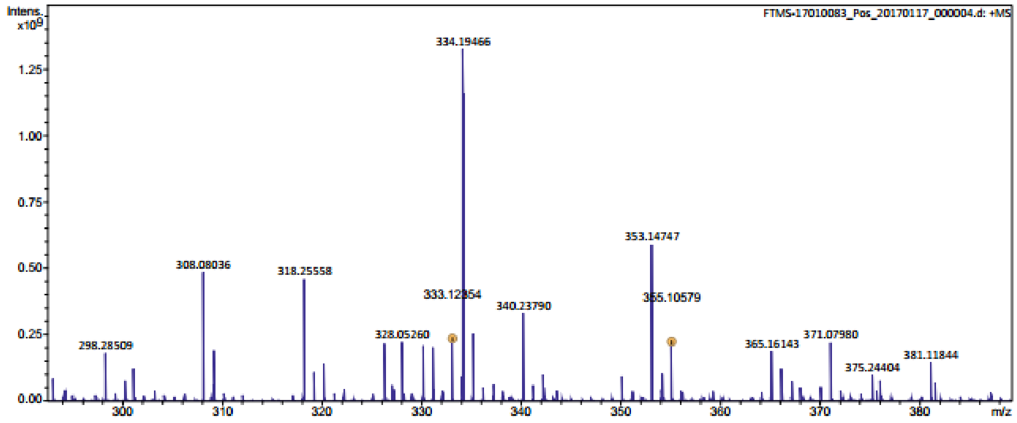

Meas. m/z	#	Ion Formula	Score	m/z	err [ppm]	Mean err [ppm]	mSigma	rdb	e⁻ Conf	N-Rule
333.123537	1	C20H17N2O3	100.00	333.123369	-0.5	-0.5	18.9	14.0	even	ok
355.105794	1	C20H16N2NaO3	100.00	355.105313	-1.4	-1.5	12.9	14.0	even	ok

8g

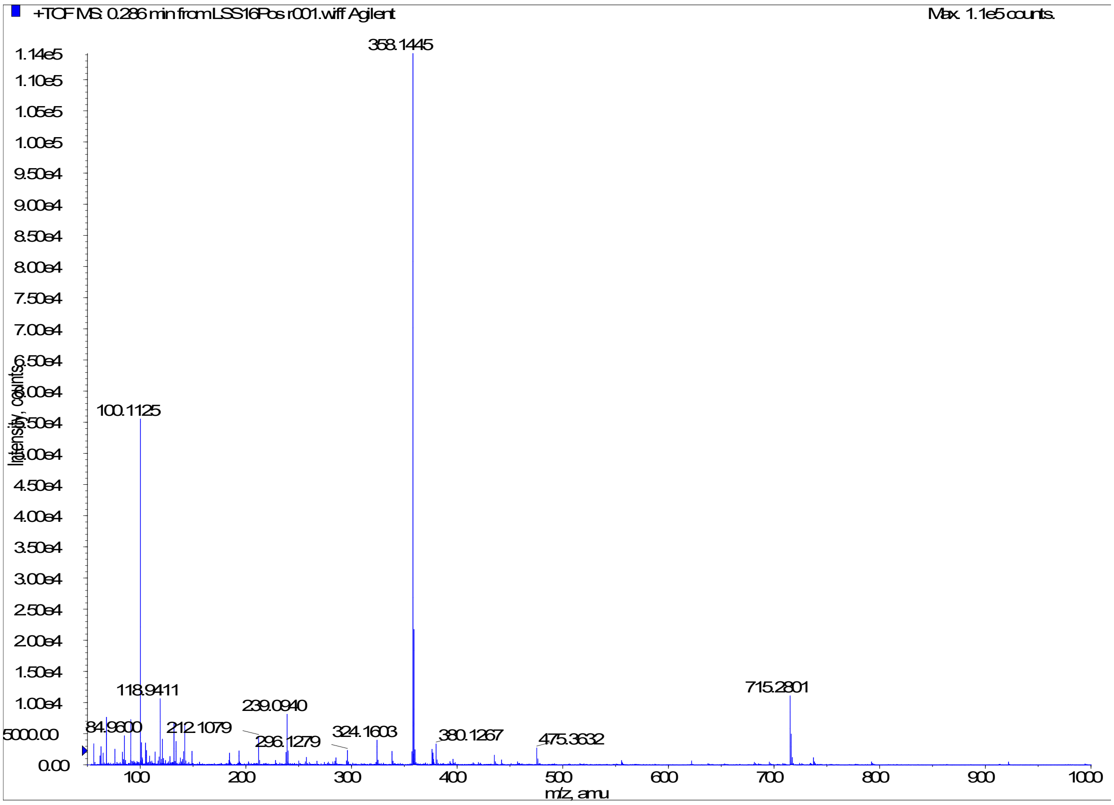

8h

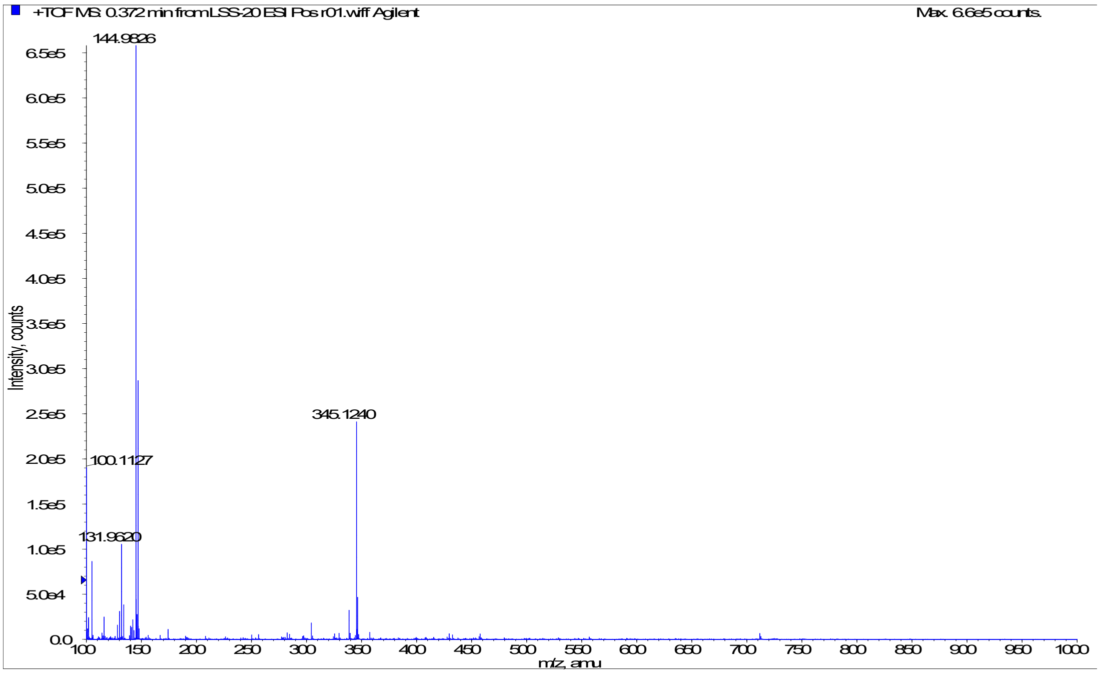

+TOF MS: 0.372 min from LSS-20 ESI Pos r01.wiff Agilent
Max. 6.6e5 counts.
144.9826
345.1240
100.1127
131.9620
Intensity, counts
m/z, amu

8j

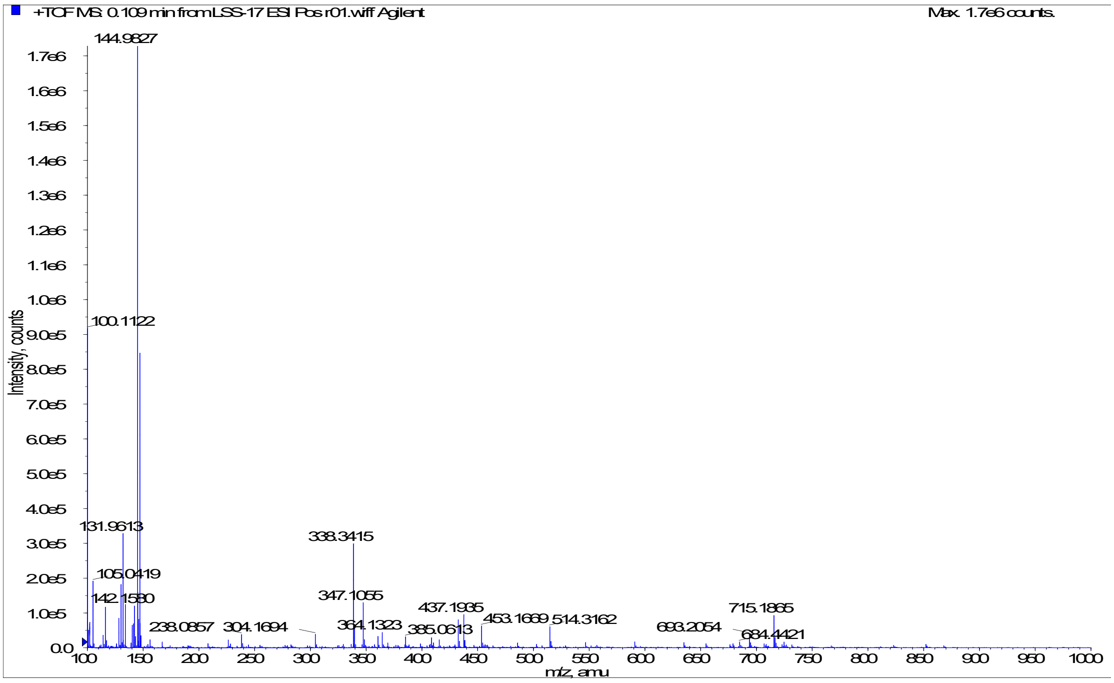

+TOF MS: 0.109 min from LSS-17 ESI Pos r01.wiff Agilent
Max. 1.7e6 counts.
144.9827
100.1122
131.9613
105.0419
142.1580
238.0857
304.1694
338.3415
347.1055
364.1323
385.0613
437.1935
453.1669
514.3162
693.2054
715.1865
684.4421
Intensity, counts
m/z, amu

8k

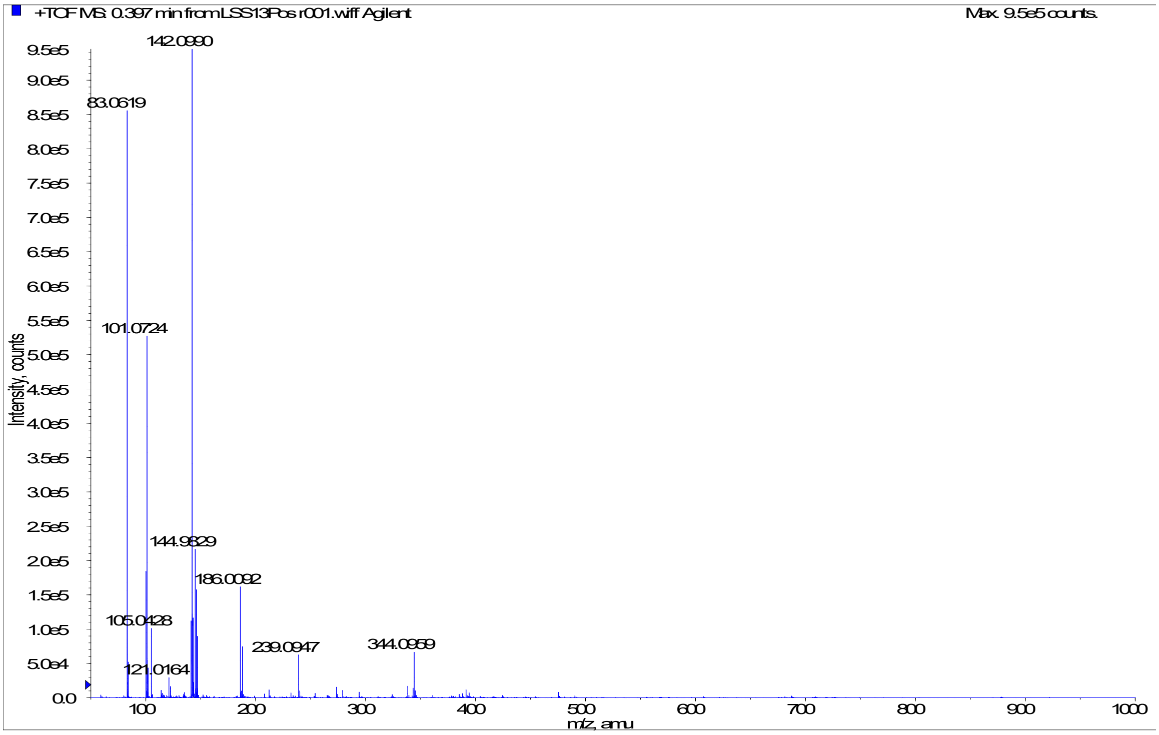
+TOF MS: 0.397 min from LSS13Pos r001.wiff Agilent
Max. 9.5e5 counts.
142.0990
83.0619
101.0724
144.9829
186.0092
105.0428
239.0947
344.0959
121.0164
Intensity, counts
m/z, amu

8l

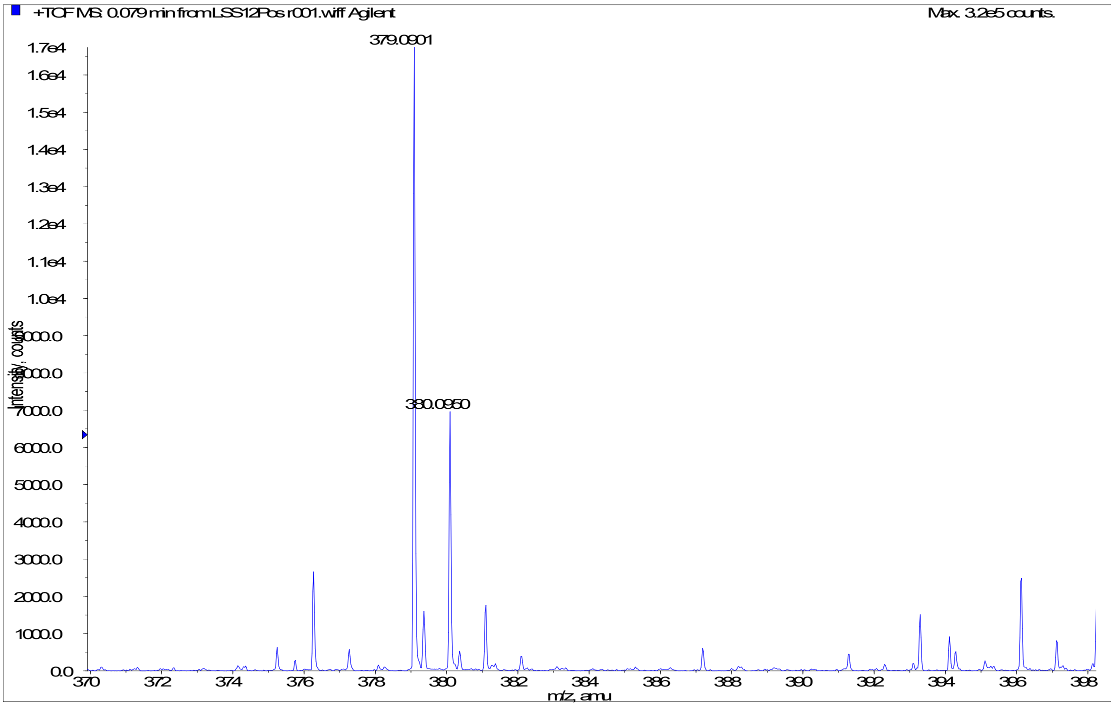
+TOF MS: 0.079 min from LSS12Pos r001.wiff Agilent
Max. 3.2e5 counts.
379.0901
380.0950
Intensity, counts
m/z, amu

9b

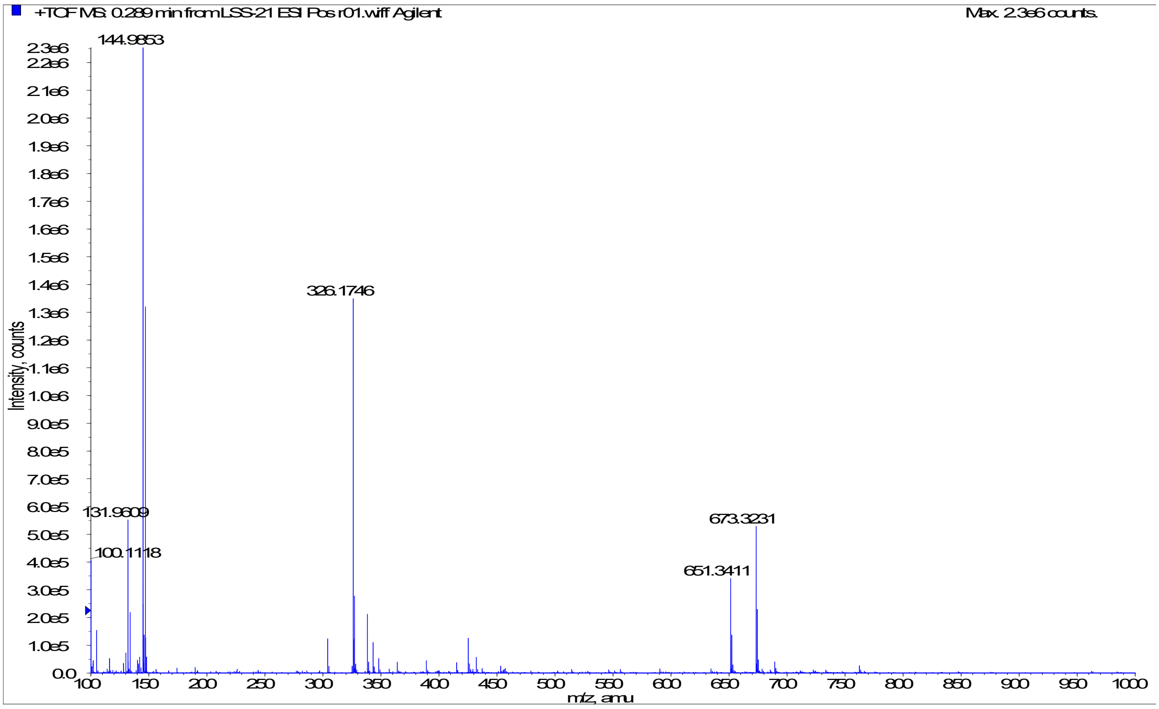
+TOF MS: 0.289 min from LSS-21 ESI Pos r01.wiff Agilent
Max. 2.3e6 counts.
Intensity, counts
m/z, amu
144.9853
326.1746
131.9609
100.1118
673.3231
651.3411

12f

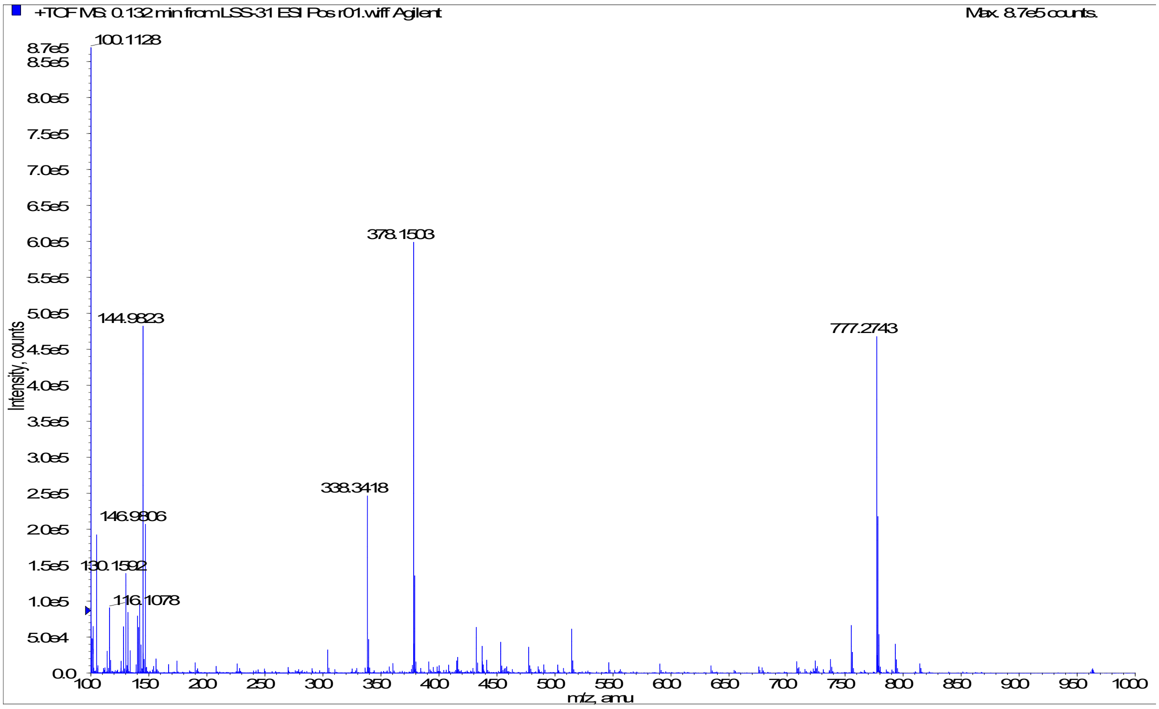
+TOF MS: 0.132 min from LSS-31 ESI Pos r01.wiff Agilent
Max. 8.7e5 counts.
Intensity, counts
m/z, amu
100.1128
378.1503
144.9823
777.2743
338.3418
146.9806
130.1592
116.1078

18b

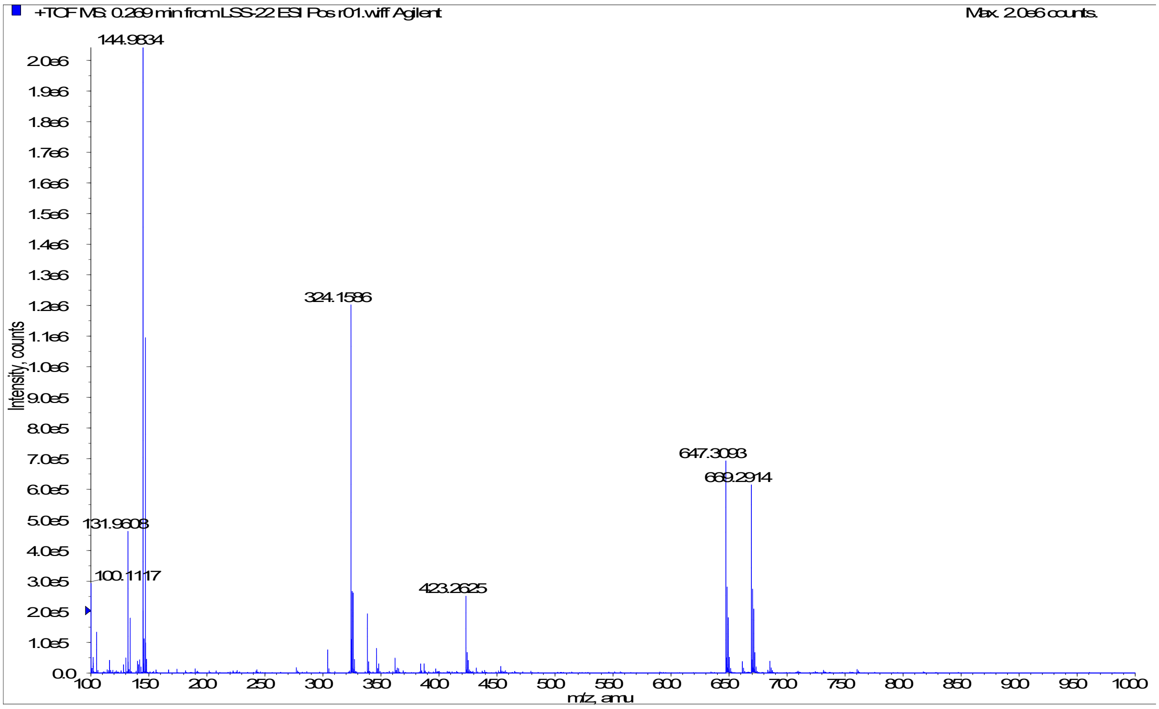
+TOF MS: 0.269 min from LSS-22 ESI Pos r01.wiff Agilent
Max. 2.0e6 counts.
144.9834
324.1586
647.3093
669.2914
131.9608
100.1117
423.2625
Intensity, counts
m/z, amu

23b

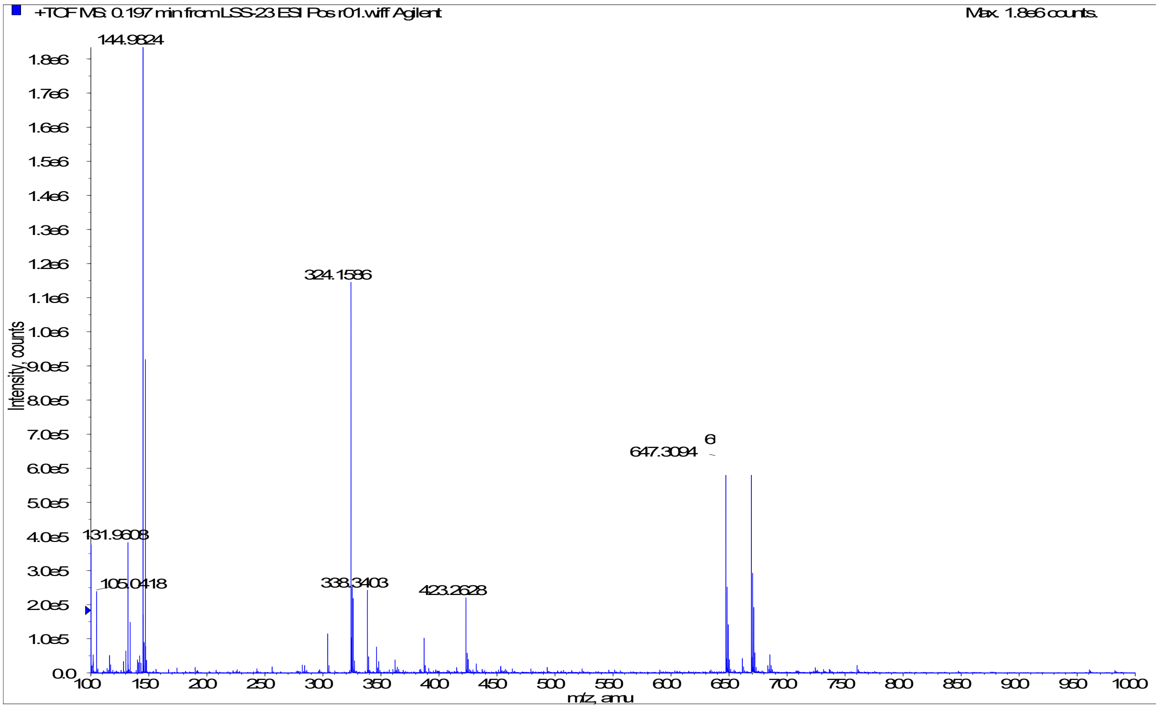
+TOF MS: 0.197 min from LSS-23 ESI Pos r01.wiff Agilent
Max. 1.8e6 counts.
144.9824
324.1586
647.3094
131.9608
105.0418
338.3403
423.2628
Intensity, counts
m/z, amu

23i

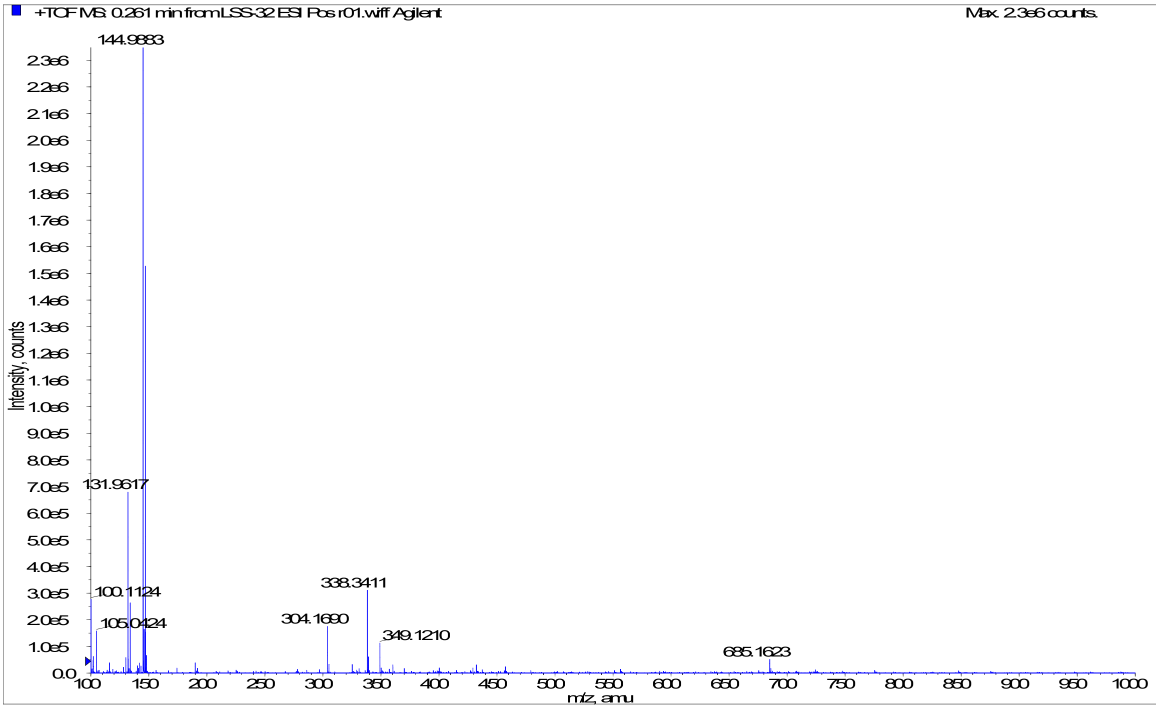
+TOF MS: 0.261 min from LSS-32 ESI Pos r01.wiff Agilent
Max. 2.3e6 counts.
144.9883
131.9617
100.1124
105.0424
304.1690
338.3411
349.1210
685.1623
Intensity, counts
m/z, amu

24b

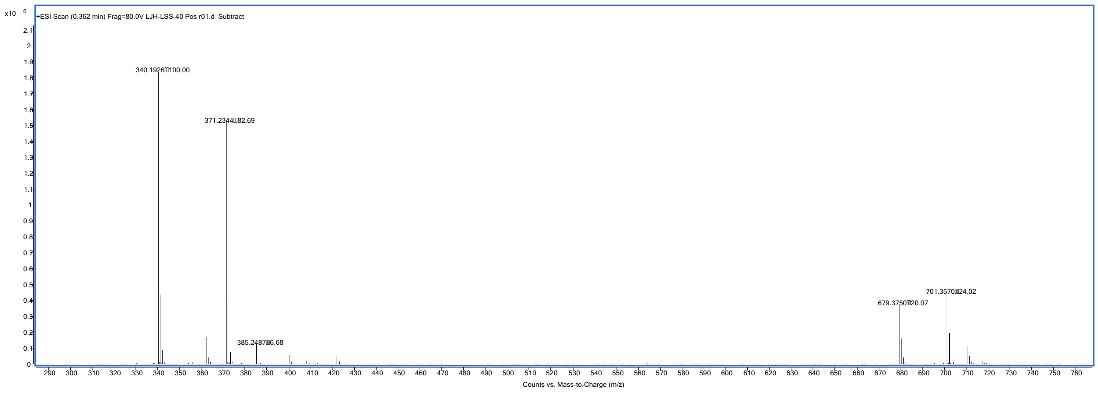
x10 6
+ESI Scan (0.362 min) Frag=80.0V LJH-LSS-40 Pos r01.d Subtract
340.1926
371.2344
385.2487
679.3750
701.3570
Counts vs. Mass-to-Charge (m/z)

29a

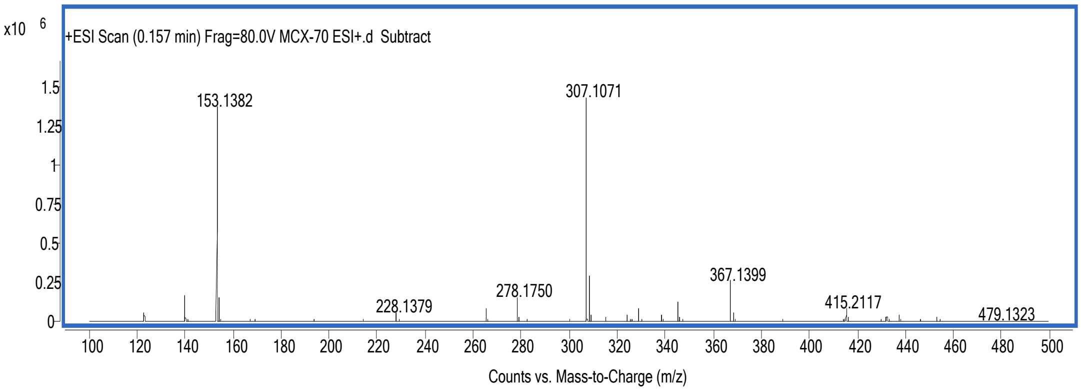
x10 6
+ESI Scan (0.157 min) Frag=80.0V MCX-70 ESI+.d Subtract
153.1382
307.1071
228.1379
278.1750
367.1399
415.2117
479.1323
Counts vs. Mass-to-Charge (m/z)

29b

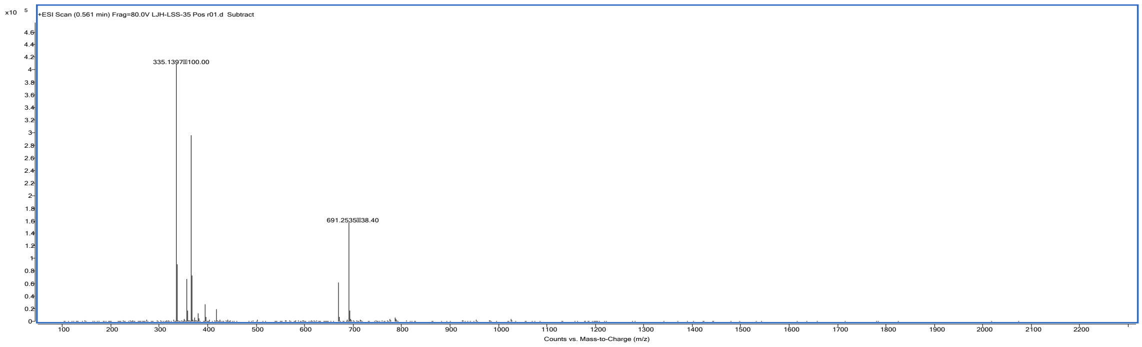

x10 5
+ESI Scan (0.561 min) Frag=80.0V LJH-LSS-35 Pos r01.d Subtract
335.1397 100.00
691.2535 38.40
Counts vs. Mass-to-Charge (m/z)

32a

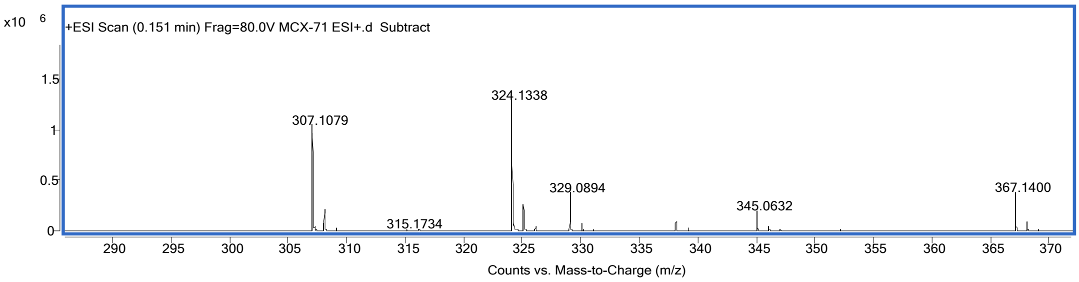

x10 6
+ESI Scan (0.151 min) Frag=80.0V MCX-71 ESI+.d Subtract
307.1079
324.1338
329.0894
315.1734
345.0632
367.1400
Counts vs. Mass-to-Charge (m/z)

32b

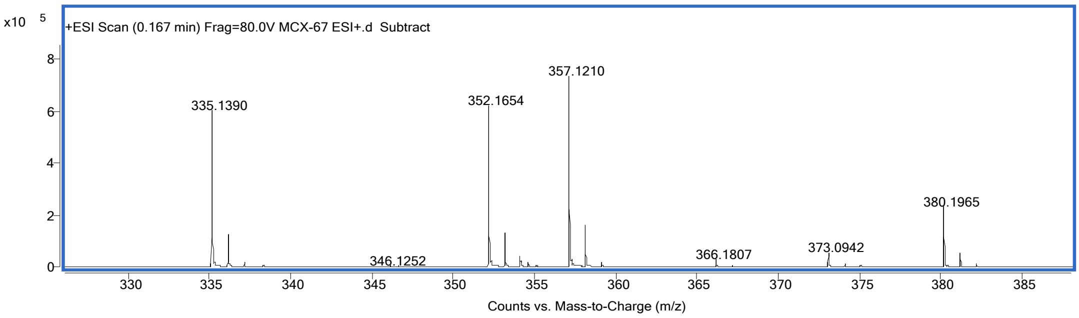

x10 5
+ESI Scan (0.167 min) Frag=80.0V MCX-67 ESI+.d Subtract
335.1390
352.1654
357.1210
346.1252
366.1807
373.0942
380.1965
Counts vs. Mass-to-Charge (m/z)

36a

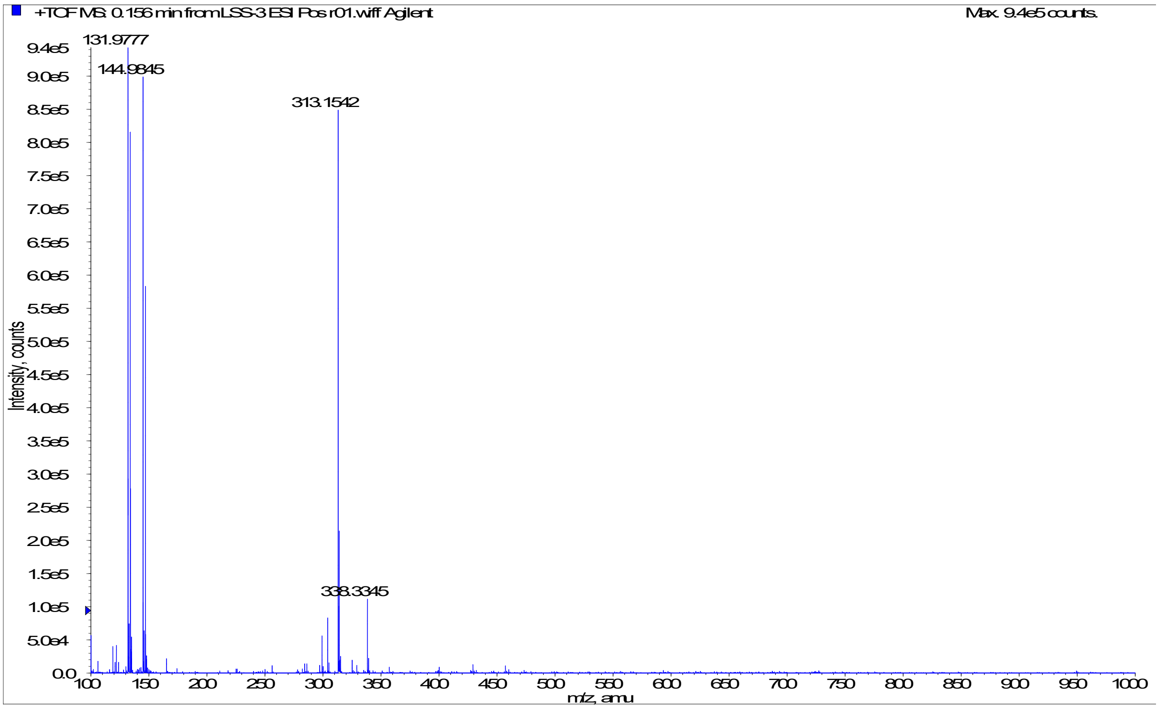

+TOF MS: 0.156 min from LSS-3 ESI Pos r01.wiff Agilent
Max. 9.4e5 counts.
131.9777
144.9845
313.1542
338.3345
Intensity, counts
m/z, amu

39b

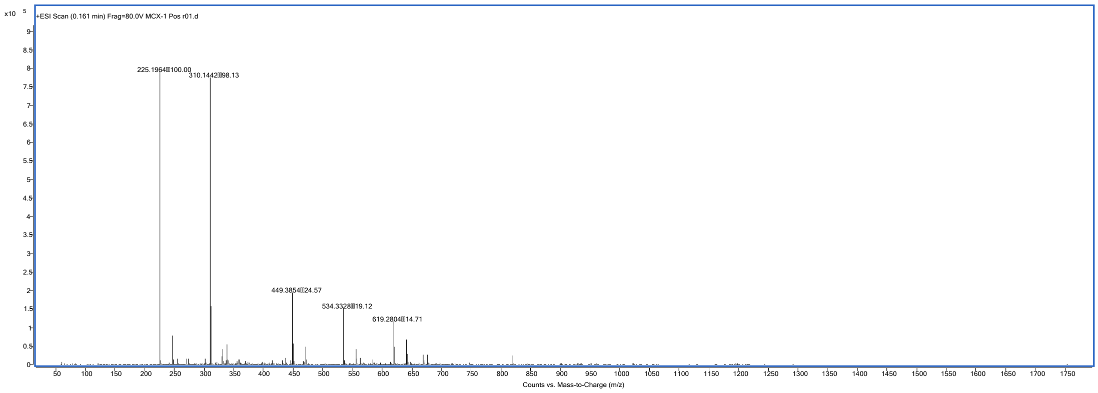

+ESI Scan (0.161 min) Frag=80.0V MCX-1 Pos r01.d
225.1964 100.00
310.1442 98.13
449.3854 24.57
534.3328 19.12
619.2804 14.71
Counts vs. Mass-to-Charge (m/z)

42b

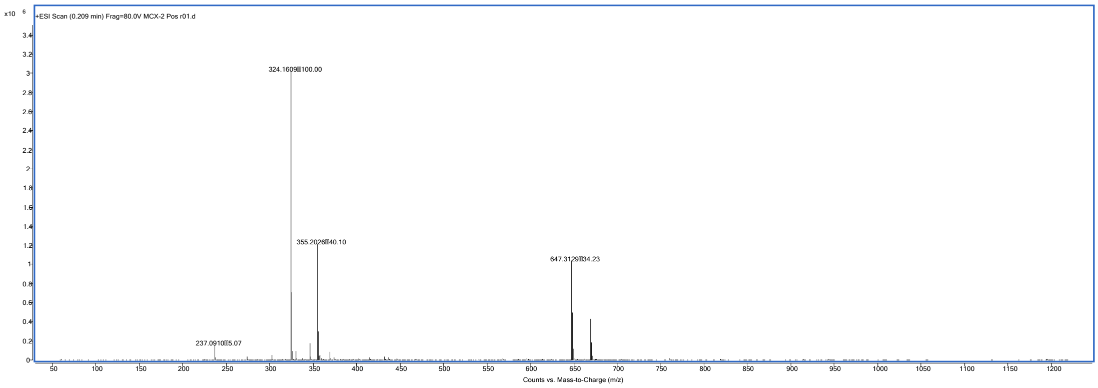

+ESI Scan (0.209 min) Frag=80.0V MCX-2 Pos r01.d
324.1609 100.00
355.2026 40.10
647.3129 34.23
237.0910 5.07
Counts vs. Mass-to-Charge (m/z)

Pharmacokinetic experimental conditions of compound **32b**.

LC-MS method: It was performed on a Thermo Scientific Q Exactive HF-X instrument, the column was an ACQUITY UPLC BEH C18 Column, 130 Å, 1.7 µm, 2.1 mm×100 mm, and the pre-column was an ACQUITY UPLC BEH C18 VanGuard Pre- column, 130Å, 1.7 µm, 2.1 mm×5 mm, and the liquid-phase was based on gradient elution with acetonitrile and 0.1% aqueous formic acid, and the mass spectrometry was performed in positive ion mode. The drug concentration in plasma samples was quantified by internal standard method and the pharmacokinetic parameters were calculated by phoenix winnonlin.

Mass spectrometry conditions: **32b** mass spectrometry acquisition mode is positive ion mode, the molecular weight of the precursor ion is $[M+H]^+$: 335.1384, the molecular weight of the daughter ion is: 265.0969.

Column Details:	ACQUITY UPLC BEH C18 Column (1.7 µm, 2.1 mm×100 mm)
Column Temperature:	30°C
Flow Rate:	0.3 mL/min
Injection volum:	3 µL

Solvent A: 0.1% formic acid in water; Solvent B: acetonitrile.

Time	Solvent A	Solvent B
0	60	40
2	60	40
3	2	98
10	2	98
11	60	40
13	60	40

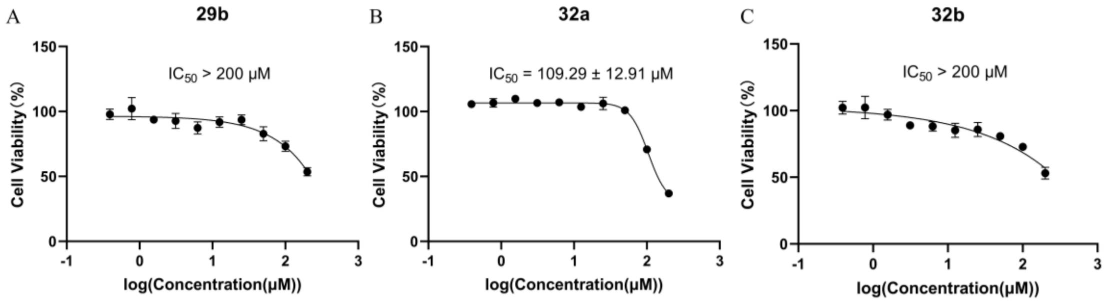

Figure S1. Results of dose-toxicity experiment for compounds **29b**, **32a**, and **32b**.
